# Eye on the horizon: The metabolic landscape of the RPE in aging and disease

**DOI:** 10.1016/j.preteyeres.2024.101306

**Published:** 2024-10-19

**Authors:** David S. Hansman, Jianhai Du, Robert J. Casson, Daniel J. Peet

**Affiliations:** a School of Biological Sciences, University of Adelaide, Adelaide, SA, Australia; b Department of Ophthalmology and Visual Sciences, Department of Biochemistry and Molecular Medicine, West Virginia University, Morgantown, WV 26506, USA; c Discipline of Ophthalmology and Visual Science, Adelaide Medical School, University of Adelaide, Adelaide, SA, Australia

**Keywords:** Age-related macular degeneration, Retinal pigment epithelium, Retina inflammation, Retina metabolism, RPE metabolism, RPE aging, Metabolic ecosystem

## Abstract

To meet the prodigious bioenergetic demands of the photoreceptors, glucose and other nutrients must traverse the retinal pigment epithelium (RPE), a polarised monolayer of cells that lie at the interface between the outer retina and the choroid, the principal vascular layer of the eye. Recent investigations have revealed a metabolic ecosystem in the outer retina where the photoreceptors and RPE engage in a complex exchange of sugars, amino acids, and other metabolites. Perturbation of this delicate metabolic balance has been identified in the aging retina, as well as in age-related macular degeneration (AMD), the leading cause of blindness in the Western world. Also common in the aging and diseased retina are elevated levels of cytokines, oxidative stress, advanced glycation end-products, increased growth factor signalling, and biomechanical stress – all of which have been associated with metabolic dysregulation in non-retinal cell types and tissues. Herein, we outline the role of these factors in retinal homeostasis, aging, and disease. We discuss their effects on glucose, mitochondrial, lipid, and amino acid metabolism in tissues and cell types outside the retina, highlighting the signalling pathways through which they induce these changes. Lastly, we discuss promising avenues for future research investigating the roles of these pathological conditions on retinal metabolism, potentially offering novel therapeutic approaches to combat age-related retinal disease.

## 1. Introduction

**Fig. 1.**
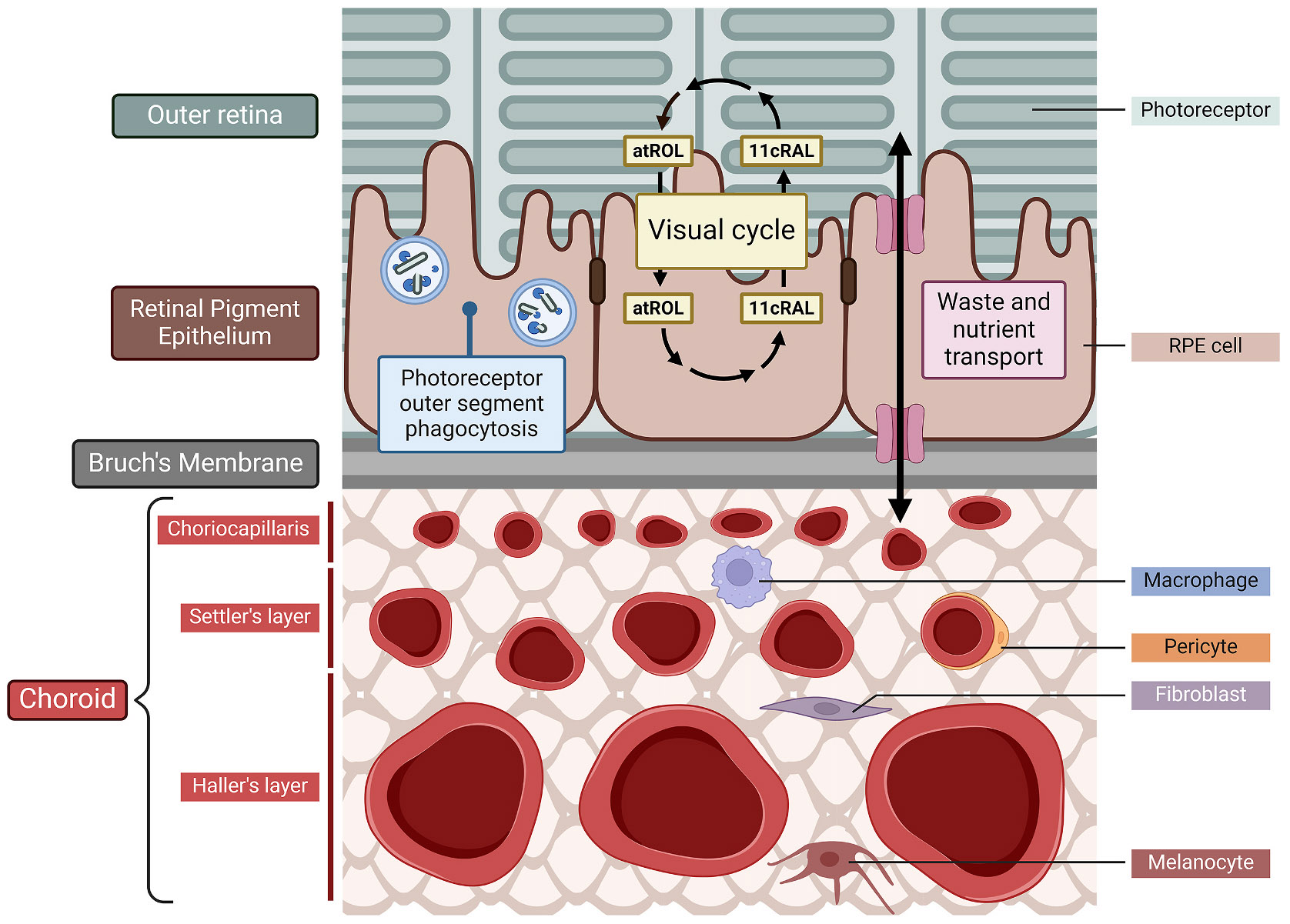
Figure 1. The roles of the RPE in maintaining retinal homeostasis. The RPE is a monolayer of cells that occupies an anatomical niche between the photoreceptors and the main vascular layer of the eye, called the choroid. The choroid consists of three main layers: the choriocapillaris, Sattler’s layer, and Haller’s layer, each containing progressively larger blood vessels. The choroid is home to several different cell types including melanocytes, fibroblasts, pericytes, and immune cells such as macrophages. The RPE fulfills several essential roles that maintain retinal homeostasis, including the phagocytosis of photoreceptor outer segments, participation in the visual cycle, and the bidirectional transport of nutrients and waste products between circulation and the outer retina. Cell types in the diagram are not drawn to scale. Abbreviations: atROL = all-trans retinol, 11cRAL = 11-cis retinal.

The retinal pigment epithelium (RPE) is a polarised monolayer of epithelial cells occupying the critical anatomical position between the photoreceptors of the outer retina and the choroid, the chief ocular blood supply (Fig. 1). The RPE adheres to Bruch’s membrane, a rigid, pentalaminar structure that provides structural support to the retina. Adjacent RPE cells form tight junctions, establishing a selective outer blood-retina barrier (BRB) that restricts passive solute diffusion (Naylor et al., 2019; Rizzolo, 2007; Rizzolo et al., 2011) and maintains the immune-privileged status of the retina (Streilein, 2003). Consequently, the RPE serves as the gatekeeper of the outer retina, a functional hub managing the bidirectional transport of nutrients and waste products between the blood supply and the photoreceptors (Lehmann et al., 2014; Strauss, 2005).

In addition to its barrier function, the RPE supports retina health and function through several other roles (Fig. 1). Photoreceptor outer segments (OSs) are comprised of stacks of membranous disks containing light-sensitive visual pigments such as rhodopsin. These disks are shed daily following a circadian rhythm, and are phagocytosed by RPE which recycles their contents (Lakkaraju et al., 2020; Young, 1967). The RPE also helps prevent light scattering after it passes through the photoreceptor layer by absorbing visible light using melanin and lipofuscin granules (Strauss, 2005; Young, 1988). Moreover, the RPE facilitates the visual cycle by regenerating 11-cis retinal (Fig. 1). In rods and cones, incoming light converts opsin-bound 11-cis retinal to all-trans retinal, triggering a biochemical cascade that results in phototransduction. All-trans retinal is then released from opsin, reduced to all-trans retinol, secreted from the photoreceptors and is taken up by the RPE, where the enzyme retinal pigment epithelium-specific 65 kDa protein (RPE65) regenerates 11-cis retinal for re-use by the photoreceptors (Tsin et al., 2018).

The choroid, which accounts for 85% of the total ocular blood supply (Bill, 1975), provides most of the glucose, oxygen, and other nutrients needed for the outer retina. Thus, glucose must traverse both Bruch’s membrane and the RPE to reach the photoreceptors which, as elaborated below, have prodigious glucose demands. The lactate released as a waste product of photoreceptor metabolism is thought to suppress glycolysis and fuel oxidative phosphorylation (OXPHOS) in the RPE for ATP production, allowing safe passage of glucose through to the photoreceptors (Kanow et al., 2017).

Our understanding of the RPE-photoreceptor metabolic relationship is constantly evolving, with these cells now believed to exchange an array of metabolites that extends beyond glucose and lactate, including amino acids, lipids, and tricarboxylic acid (TCA) cycle intermediates (Chao et al., 2017; Hass et al., 2022; Pan et al., 2021; Sinha et al., 2020b; Viegas and Neuhauss, 2021; Yam et al., 2019; Zhang et al., 2021). Disentangling the retina’s metabolic environment may have significant clinical implications as retinal metabolic dysregulation has emerged as a key factor in the pathogenesis of several eye diseases, including age-related macular degeneration (AMD) (Cheng et al., 2020; Ferrington et al., 2017; Fisher and Ferrington, 2018; Golestaneh et al., 2017; Léveillard et al., 2019; Zhang et al., 2020).

### 1.1. The RPE and aging

Throughout the aging process, the RPE and underlying Bruch’s membrane undergo numerous physiological changes, many of which are also associated with diseases such as AMD. Characteristics of normal retinal aging and AMD exist on a continuum, with an ambiguous boundary separating both (Sura et al., 2020). One such characteristic is the formation of basal deposits and drusen, extracellular lipid and protein deposits that accumulate to the basal side of the RPE (Curcio and Johnson, 2012). Drusen in particular serve as inflammatory nodes, providing a chronic low-grade inflammatory stimulus (Anderson et al., 2002). Components of basal deposits and drusen, cellular senescence, and damage-associated molecular patterns (DAMPs) all stimulate the release of an ensemble of cytokines, most prominently tumour necrosis factor alpha (TNFα), transforming growth factor beta (TGF-β), interleukin 6 (IL-6) and interleukin 1 beta (IL-1β), from both infiltrating immune cells and the RPE itself (Lee et al., 2021).

Phagocytosis of photoreceptor OSs, a heavy reliance on OXPHOS for energy production, and high NADPH oxidase activity, expose the RPE to oxidative stress, even under benign, physiological conditions. This stress intensifies with age due to declining antioxidant enzyme activity, mitochondrial dysfunction, and the accumulation of peroxidised lipids and lipofuscin granules (Castorina et al., 1992; Hyttinen et al., 2018; Liles et al., 1991; Winkler et al., 1999). Oxidative stress is not only considered a key driver of the aging process in general but is also emerging as a key factor in AMD aetiology (Datta et al., 2017; Kregel and Zhang, 2007).

Advanced glycation end-products (AGEs) accumulate on collagen and other Bruch’s membrane components with age, interacting with the receptor for advanced glycation end-products (RAGE) on the RPE surface (Farboud et al., 1999; Glenn et al., 2009; Howes et al., 2004). Furthermore, growth factors such as epidermal growth factor (EGF), fibroblast growth factor (FGF), and insulin-like growth factor (IGF) may be dysregulated in the aged retina and are elevated in various ocular diseases, including AMD (Amin et al., 1994; Lambooij et al., 2003; Rosenthal et al., 2004). Lastly, Bruch’s membrane increases in stiffness with age, resulting in rising biomechanical stress in the RPE (Tarau et al., 2019; Ugarte et al., 2006).

These aging and disease-related pathological changes may also function as drivers of dysregulated RPE metabolism. Factors such as biomechanical and oxidative stress, AGEs, growth factors, and cytokines activate intracellular signalling networks within the RPE. As elaborated below, these pathways have been shown to instigate metabolic dysregulation in numerous non-retinal cell types and similarly may disturb the fragile equilibrium of the outer retina. In this review we sequentially evaluate these factors, exploring the signalling pathways they activate, their influence on metabolism in other cells and tissues, their connections to retinal aging and disease, and their potential impact on RPE metabolism.

## 2. Metabolism in the retina

### 2.1. Metabolism in the photoreceptors

**Fig. 2.**
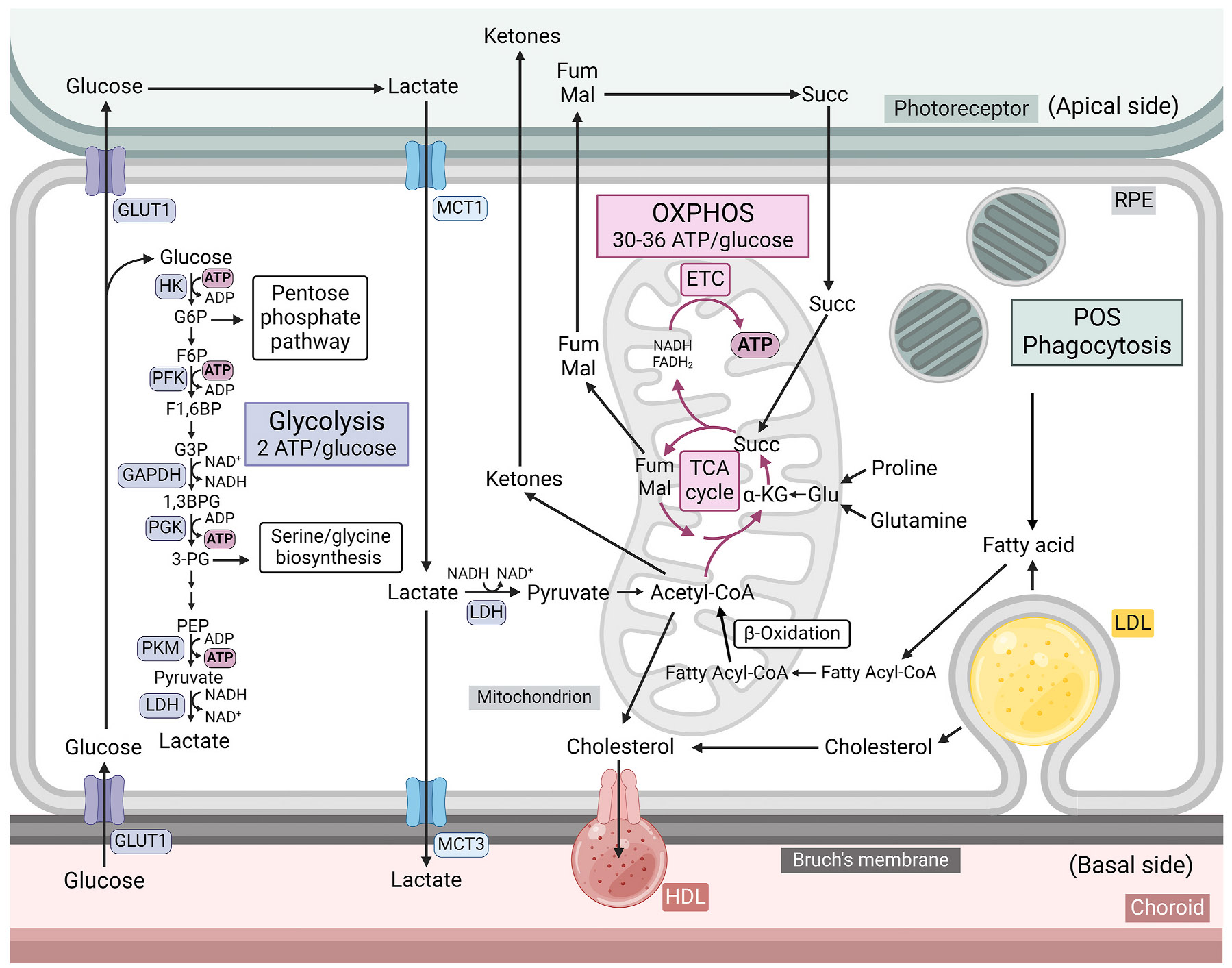
Figure 2. Metabolism in the RPE. Most of the glucose taken up by the RPE from the choroid is transported to the apical side of the RPE monolayer to fuel the photoreceptors. However, some of the glucose undergoes glycolysis in the RPE to provide precursors for the pentose phosphate pathway and serine/glycine biosynthesis. Lactate produced as a byproduct of photoreceptor metabolism is imported into the RPE through MCT1 transporters and can either be exported to the basal side through MCT3 transporters or be converted to pyruvate to provide a substrate for OXPHOS. OXPHOS is a highly efficient means of energy production, generating 30-36 moles of ATP per mole of glucose, compared to 2 moles of ATP generated by glycolysis. Another important fuel for RPE cells are fatty acids which can originate from endocytosed LDL particles or phagocytosed photoreceptor outer segments. Fatty acids undergo β-oxidation in the RPE to generate acetyl-CoA which can enter the TCA cycle or be used for the synthesis of ketone bodies or cholesterol. Ketone bodies can be exported to the apical side of the RPE to support photoreceptor metabolism, while cholesterol can be exported via the formation of HDL-like particles. Proline and glutamine may also be consumed by the RPE, providing carbon and nitrogen for the synthesis of TCA intermediates and other amino acids. Fumarate and malate may be exported from the RPE to the photoreceptors where they are converted into succinate. The succinate is then taken up by the RPE, shuttling reducing power from the oxygen-poor retina to the oxygen-rich RPE. Abbreviations: α-KG, alpha ketoglutarate; ADP, adenosine diphosphate; ATP, adenosine triphosphate; ETC, electron transport chain, FADH_2_, flavin adenine dinucleotide (reduced); Fum, fumarate; GAPDH, glyceraldehyde 3-phosphate dehydrogenase; Glu, glutamate; GLUT1, glucose transporter 1; HDL, high-density lipoprotein; HK, hexokinase; LDH, lactate dehydrogenase; LDL, low-density lipoprotein; Mal, malate; MCT1, monocarboxylate transporter 1; MCT3, monocarboxylate transporter 3; NAD^+^, nicotinamide adenine dinucleotide (oxidised); NADH, nicotinamide adenine dinucleotide (reduced); OXPHOS, oxidative phosphorylation; PFK, phosphofructokinase; PGK, phosphoglycerate kinase; PKM, pyruvate kinase muscle isotype; POS, photoreceptor outer segment; RPE, retinal pigment epithelium; Succ, succinate; TCA, tricarboxylic acid.

The energy demands of the photoreceptors are among the highest of any cells in the body (Ames, 2000). ATP requirements are particularly elevated in the dark, when constant depolarisation is needed to sustain the ‘dark current’, with each rod and cone cell estimated to consume 8 × 10^7^ and 1.5 × 10^8^ molecules of ATP per second, respectively (Okawa et al., 2008). These immense demands may partially explain why the mammalian retina exhibits aerobic glycolysis, or the ‘Warburg effect’ – an unusual metabolic phenomenon where, despite sufficient oxygen, most consumed glucose is converted to lactate (Haydinger et al., 2020; Hurley et al., 2015; Ng et al., 2015; Vander Heiden et al., 2009). Typically associated with cancer and other proliferating cells, this mode of glucose utilisation could be an adaptive mechanism to support photoreceptor function. Although glycolysis yields less ATP per glucose molecule (Fig. 2), it can generate ATP up to 100 times faster than OXPHOS (Hurley et al., 2015; Okawa et al., 2008; Pfeiffer et al., 2001; Vander Heiden et al., 2009), which may be necessary for optimal photoreceptor performance.

In addition to glycolysis, photoreceptors rely heavily on OXPHOS, with the retina having among the highest O_2_ consumption rates of any tissue in the body, exceeding even the brain (Damsgaard and Country, 2022; Wong-Riley, 2010). O_2_ consumption increases in the dark when ATP demands are highest (Linsenmeier, 1986; Medrano and Fox, 1995), suggesting OXPHOS contributes to maintaining the dark current. Analysis of the O_2_ consumption rate suggests that this tissue operates near its oxidative capacity (Kooragayala et al., 2015), which could necessitate further glycolysis for additional ATP production. While pyruvate oxidation is essential for photoreceptor survival and visual function (Grenell et al., 2019), photoreceptors also utilise β-oxidation of fatty acids and ketone bodies to satisfy their ATP demands, a process that likewise requires OXPHOS (Fu et al., 2019; Joyal et al., 2016). It is likely that ATP generation employs a combination of sources, as photoreceptors must be malleable enough to adapt to rapid changes in ATP demand, which can vary several-fold between dark and light conditions (Okawa et al., 2008).

Aerobic glycolysis may also supply metabolic intermediates that help support the biosynthetic demands of the photoreceptors (Vander Heiden et al., 2009). Each photoreceptor OS, complete with hundreds of stacked membranous disks with light-sensitive opsin proteins, undergoes complete renewal approximately every 10 days (Young, 1967) – imposing a considerable biosynthetic burden on these cells. Glycolytic intermediates can be shunted into biosynthetic pathways, providing carbons for the glycerol backbone of membrane phospholipids (Baenke et al., 2013), and the glycosylation of proteins such as rhodopsin (Murray et al., 2009; Wellen et al., 2010). Glycolytic intermediates are also used to synthesise several amino acids, such as serine, glycine, and alanine (Lunt and Vander Heiden, 2011; Yang and Vousden, 2016). Supporting the view that photoreceptors utilise aerobic glycolysis to fulfil biosynthetic demands, genetic ablation of lactate dehydrogenase A (LDHA) disrupts photoreceptor OS biogenesis, while its inhibition in retinal explants does not affect ATP production (Chinchore et al., 2017).

### 2.2. RPE metabolism

#### 2.2.1. The RPE exhibits low glycolysis

In comparison to the photoreceptors which favour aerobic glycolysis, native RPE cells are believed to exhibit comparatively low rates of glycolysis (Fig. 2). *Ex vivo* murine RPE/choroid tissue and cultured human foetal RPE (hfRPE) cells export lactate at a rate approximately 20-fold slower than retinal tissue (Kanow et al., 2017). Expression of glycolytic genes is lower in human RPE/choroid tissue compared to the neural retina (B. Li et al., 2020a) and *in vivo* deletion of the glucose transporter GLUT1 in mouse RPE cells does not affect RPE differentiation, polarity, or function (Swarup et al., 2019). Instead, the RPE is believed to support its energy demands primarily through mitochondrial OXPHOS. Mitochondria are abundant in RPE cells, representing 11.5% of total cytoplasmic volume (Keeling et al., 2020). Cultured RPE cells produce the majority of their ATP through OXPHOS compared to glycolysis (Hazim et al., 2022) and genetic ablation of OXPHOS in mouse RPE cells causes dedifferentiation and cellular hypertrophy (C. Zhao et al., 2011a).

#### 2.2.2. The RPE uses fatty acids for β-oxidation

As elaborated below, the RPE must exhibit flexibility in order to adapt to the changing metabolic conditions of the outer retina. β-oxidation of fatty acids provides substrates for mitochondrial OXPHOS in the form of NADH and FADH_2_, as well as acetyl-CoA which can enter the TCA cycle (Houten et al., 2016). Mouse RPE/choroid tissue exhibits more abundant fatty acid metabolites and elevated β-oxidation gene expression compared to the neural retina (Sinha et al., 2020b). Moreover, cultured porcine RPE cells express the β-oxidation enzyme complex, mitochondrial trifunctional protein (MTP), and are capable of the uptake and metabolism of palmitic acid (Tyni et al., 2002). MTP, as well as carnitine palmitoyltransferase 1 (CPT1) protein are also detectable in cultured human RPE cells (Roomets and Kivela, 2008). ^13^C-labelled palmitate carbons are incorporated into TCA intermediates in cultured hfRPE cells, while increasing the oxygen consumption rate (OCR), consistent with palmitate being used to fuel the TCA cycle and OXPHOS (Adijanto et al., 2014). Genes encoding β-oxidation enzymes and mitochondrial respiration components in cultured human RPE cells are induced with overexpression of peroxisome proliferator-activated receptor-gamma coactivator 1 alpha (PGC-1α), a master regulator of mitochondrial function and oxidative metabolism (Handschin and Spiegelman, 2006; Iacovelli et al., 2016).

#### 2.2.3. The RPE uses lactate to fuel OXPHOS

In addition to fatty acids, mitochondrial OXPHOS in the RPE may also be fuelled by lactate. Monocarboxylate transporter 1 (MCT1), which has a high affinity for lactate, is expressed on the apical surface of RPE cells (Adijanto and Philp, 2012), allowing efficient import from the neural retina. The lactate dehydrogenase subunit LDHB, which favours the conversion of lactate to pyruvate (van Wilpe et al., 2021), exhibits elevated expression relative to LDHA in the RPE (Rajala et al., 2023). The dominance of the LDHB isoform over LDHA indicates that the RPE consumes lactate, converting it to pyruvate to fuel mitochondrial OXPHOS. Indeed, treatment of cultured hfRPE cells with ^13^C-labelled lactate shows that lactate carbons are incorporated into TCA cycle intermediates (Kanow et al., 2017). Moreover, lactate treatment substantially elevates the OCR in *ex vivo* murine RPE/choroid tissue (Bisbach et al., 2020), supporting the view that it is used as an oxidative substrate by the RPE.

#### 2.2.4. The RPE synthesises and catabolises amino acids

Genes involved in the serine biosynthesis pathway, as well as serine biosynthesis intermediates, are upregulated in RPE/choroid tissue compared to the neural retina (B. Li et al., 2020b; Sinha et al., 2020b). In addition to its role in the biosynthesis of other amino acids such as glycine, cysteine, and taurine (Locasale, 2013), serine is a precursor for the synthesis of several other components vital to RPE homeostasis and function, including phosphatidylserine (Sinha et al., 2020a), sphingolipids (Sinha et al., 2020a), and the antioxidant glutathione (Locasale, 2013; Sun et al., 2018). Moreover, serine synthesised from glucose in the RPE is transported to the apical side of the cell, presumably for photoreceptor use (Chao et al., 2017; B. Li et al., 2020b).

Proline is consumed more than any other metabolite in the medium of hfRPE cells (Chao et al., 2017). It is imported into the RPE through the transporter SLC6A20 (Yam et al., 2019) where it is converted to α-ketoglutarate (α-KG) and enters the TCA cycle where it undergoes reductive carboxylation (Yam et al., 2019). Interestingly, proline treatment stimulates the synthesis of alanine, serine, and glycine, and inhibition of proline catabolism impairs glucose utilisation in cultured human RPE cells (Yam et al., 2019). As with proline, cultured RPE cells also consume glutamine and metabolise it through mitochondrial reductive carboxylation (Du et al., 2016). On the other hand, *ex vivo* RPE/choroid tissue can synthesise glutamine when treated with high concentrations of NH_4_Cl (Xu et al., 2020). Metabolite tracing experiments using ^15^N-labelled proline, glutamine, aspartate, and alanine indicate that in *ex vivo* RPE/choroid tissue these amino acids can be used to synthesise each other, other non-essential amino acids, as well as branched-chain amino acids (BCAAs) (Xu et al., 2020; Zhu et al., 2023).

#### 2.2.5. The RPE imports, synthesises, and exports cholesterol

The RPE can both synthesise cholesterol *de novo* through the mevalonate pathway or take up cholesterol from circulation through low-density lipoprotein receptor (LDLR)-mediated endocytosis of LDL (Gordiyenko et al., 2004; Ramachandra Rao and Fliesler, 2021; Tserentsoodol et al., 2006). The RPE expresses genes involved in cholesterol homeostasis such as sterol regulatory element binding proteins 1 and 2 (*SREBP1* and *SREBP2*), and cholesterol synthesis such as HMG-CoA reductase (*HMGCR*) (Zheng et al., 2012). The RPE also participates in reverse cholesterol transport, exporting unesterified cholesterol to the apical and basal side through cholesterol transporters such as ATP-binding cassette transporter A1 (ABCA1), ATP-binding cassette transporter G1 (ABCG1), and scavenger receptor class B type I (SR-BI) (Duncan et al., 2009; Lyssenko et al., 2018). Cholesterol is exported to high-density lipoprotein (HDL)-like particles which contain the apolipoproteins ApoA1 and ApoE (Ishida et al., 2004, 2006). Treatment of cultured RPE cells with HDLs or purified ApoA1 protein is sufficient to stimulate efflux of cholesterol and other lipids (Duncan et al., 2009; Ishida et al., 2006; Storti et al., 2017). Cholesterol metabolism and export from the RPE is disrupted during aging and contributes to the pathogenesis of AMD, which is discussed in more detail in later sections.

#### 2.2.6. The RPE exhibits metabolic plasticity

Although it is believed to rely primarily on the oxidation of lactate and fatty acids to satisfy its energy demands, RPE cells also consume a portion of the glucose that they import from circulation. The removal of glucose from the media of cultured rat RPE cells decreases viability by approximately 30% (Wood et al., 2004). This effect is not rescued by the addition of lactate or pyruvate which indicates that RPE cells require glucose for survival. The RPE synthesises and stores glycogen from glucose, which has been suggested to function as a glucose buffer for the outer retina (Kanow et al., 2017; Senanayake et al., 2006). Interestingly, murine RPE/choroid and neural retina tissues exhibit comparable levels of some glycolytic intermediates such as fructose 1,6-bisphosphate, 3-phosphoglycerate, and phosphoenolpyruvate (Sinha et al., 2020b). The glycolytic reserve capacity of primary human RPE cells has been variously reported as between 10% and 50% higher than the basal glycolytic rate (Ferrington et al., 2017; Shu et al., 2022; Zhang et al., 2021), suggesting these cells are able to handle increased glycolytic flux in response to changing conditions. Several metabolic pathways which branch from glycolytic intermediates are upregulated in the RPE compared to the neural retina, including the serine biosynthesis pathway as previously mentioned. Another such pathway is the pentose phosphate pathway (PPP) which is fed by glucose 6-phosphate (Sinha et al., 2020b). As well as supplying carbons for nucleotide biosynthesis, the PPP is one of the principle sources of cellular NADPH (Fan et al., 2014; Ge et al., 2020), an essential cofactor for the glutathione and thioredoxin antioxidant systems (Ju et al., 2020). Treatment of cultured human RPE cells with ^13^C-labelled glucose shows a 20% flux through the PPP (Miceli et al., 1990). The drastically changing metabolic conditions likely necessitates metabolic flexibility in the RPE in order to maintain the retinal metabolic ecosystem.

### 2.3. The retinal metabolic ecosystem

#### 2.3.1. Lactate and glucose exchange

The past decade has seen the establishment of a ‘metabolic ecosystem’ model describing metabolic symbiosis between the RPE and photoreceptors (Chao et al., 2017; Fisher and Ferrington, 2018; Jaroszynska et al., 2021; Kanow et al., 2017; Léveillard et al., 2019; Nolan et al., 2022; Pan et al., 2021; Viegas and Neuhauss, 2021) (Fig. 2). This model proposes that glycolysis in the RPE is suppressed, enabling glucose from the choroid to traverse the RPE monolayer. The highly glycolytic photoreceptors rapidly consume this glucose and convert a large proportion to lactate, which is then taken up by the RPE (Philp et al., 1998). The lactate can either be converted to pyruvate in the RPE by LDHB (Rajala et al., 2023; van Wilpe et al., 2021) to fuel OXPHOS, or be exported to the basal side of the RPE through MCT3 for clearance into circulation (Philp et al., 1998). Lactate clearance into circulation is critical as *Mct3*^*−/−*^ mice show a buildup of retinal lactate and diminished scotopic electroretinogram (ERG) responses (Daniele et al., 2008). In support of the metabolic ecosystem hypothesis, addition of extracellular lactate to the apical side suppresses glycolysis in hfRPE cells, allowing glucose to traverse the monolayer (Kanow et al., 2017). Moreover, increasing glycolysis in the RPE through loss of OXPHOS or activation of the glycolytic transcription factor hypoxic inducible factor 1α (HIF-1α) causes photoreceptor degeneration in mice (Kurihara et al., 2016; C. Zhao et al., 2011b). This suggests that increasing RPE glycolysis may restrict glucose availability for the photoreceptors or render RPE cells unable to generate sufficient ATP from glycolysis alone, impairing their ability to support the photoreceptors.

#### 2.3.2. Succinate and malate shuttling

Recent investigations have revealed that the scope of the metabolic ecosystem extends beyond the exchange of glucose and lactate. For example, *ex vivo* murine retinas and eyecups were used to uncover a metabolite shuttle whereby succinate is used to transfer reducing power from the oxygen-poor retina to the oxygen-rich RPE (Bisbach et al., 2020, 2022; Hass et al., 2022). Succinate is exported from the retina through MCT1 (Bisbach et al., 2022) and taken up by the RPE, before being converted to fumarate and malate in a process seemingly uncoupled from ATP synthesis (Hass et al., 2022). The RPE then releases fumarate and malate, which is consumed in the retina by a reverse succinate dehydrogenase reaction (Bisbach et al., 2020).

#### 2.3.3. Outer segment phagocytosis and ketogenesis

Outer segments are shed daily by the photoreceptors with phagocytosis by the RPE peaking just after light onset (Lakkaraju et al., 2020; LaVail, 1976). As a result, large quantities of lipid and protein components are deposited in the RPE following a day/night cycle (Fliesler and Anderson, 1983). Activation of AKT signalling in the RPE by phosphatidylserine on outer segment tips promotes GLUT1 trafficking to the apical surface of the RPE, linking OS phagocytosis to glucose transport (W. Wang et al., 2019a). The RPE exhibits considerable lysosomal acid lipase activity enabling the efficient hydrolysis of outer segment fats into free fatty acids (FFAs) (Elner, 2002; Hayasaka et al., 1977). Transcriptional regulation of fatty acid oxidation (FAO) may be regulated by peroxisome proliferator-activated receptor gamma (PPARγ) in RPE cells, which is induced by photoreceptor OS phagocytosis (Ershov and Bazan, 2000).

In addition to using fatty acids to fuel OXPHOS, the RPE may use fatty acids to synthesise ketone bodies. Cultured human RPE cells express high levels of HMG-CoA synthase 2, the rate-limiting enzyme for ketogenesis (Adijanto et al., 2014). hfRPE cells use palmitate to synthesise the ketone body β-hydroxybutyrate (β-HB), which is exported preferentially to the apical side (Adijanto et al., 2014). Moreover, the RPE cell line ARPE-19 releases β-HB in a dose-dependent manner when treated with purified photoreceptor OSs (Reyes-Reveles et al., 2017). The retina imports β-HB through MCT7 and express high levels of the ketone catabolism enzyme 3-oxoacid CoA transferase 1 (OXCT1) (Adijanto et al., 2014). Treatment of explanted mouse retinas with ^13^C-labelled β-HB revealed that the retina uses this ketone body to synthesise several TCA cycle intermediates, as well as the amino acids glutamate and aspartate (Adijanto et al., 2014; Grenell et al., 2019). These findings highlight how the fatty acids derived from photoreceptor outer segments are used to synthesise ketone bodies in the RPE, which are in turn used to support photoreceptor metabolism.

#### 2.3.4. Proline metabolism

As mentioned previously, cultured hfRPE cells consume more proline than any other nutrient present in the cell culture medium (Chao et al., 2017). Tracing of ^13^C-labelled proline reveals that this amino acid is used to synthesise citrate, α-KG, glutamate, and other metabolites which are then exported to the apical side, suggesting they are synthesised to support the photoreceptors (Chao et al., 2017). Treatment of cultured human RPE cells and *ex vivo* murine RPE/choroid tissue with ^15^N-labelled proline indicates that proline is also used as a nitrogen source for the synthesis of a number of amino acids including glutamate, aspartate, glutamine, alanine, and serine (Zhu et al., 2023). Treatment of *ex vivo* murine RPE/choroid and retina tissue with both ^13^C- and ^15^N-labelled proline suggests that in comparison to the RPE/choroid, the retina takes up very little proline directly (Yam et al., 2019; Zhu et al., 2023). Indeed, analysis of these tissues following bolus injection of ^15^N-proline in mice shows ^15^N labelling of glutamate, glutamine, alanine, aspartate, and serine in both tissues, however the RPE/choroid exhibited peak labelling of these metabolites at 15 min post-injection before declining, while the neural retina labelling peaked at 30 min (Zhu et al., 2023). Taken together, these data suggest that the RPE catabolises proline into amino acids and TCA cycle intermediates which are subsequently exported for use by the photoreceptors.

#### 2.3.5. RPE mitochondria mediate metabolic communication

Recent investigations have highlighted the crucial role of RPE mitochondria in mediating metabolic exchange between the RPE and photoreceptors. As previously mentioned, *in vitro* labelling studies indicate that RPE cells use glutamine and proline to synthesise TCA cycle metabolites and amino acids to support the outer retina. Moreover, ^13^C-labelled glucose tracing reveals that glucose-derived TCA cycle intermediates, such as citrate and malate, as well as glutamate and glutamine, are exported into medium of hfRPE and primary human RPE cells (Chao et al., 2017; Zhang et al., 2021). Inhibition of mitochondrial respiratory complex I prevents the export of citrate, glutamate, pyruvate, and other metabolites to the apical side of hfRPE monolayers (Chao et al., 2017). Inhibition of respiratory complexes I, III, and V elicits similar effects on the intracellular and extracellular metabolites of cultured primary human RPE cells, impacting most strongly those involved in glucose and amino acid metabolism (Zhang et al., 2021). The effects of mitochondrial inhibition on these metabolites are mixed, leading to decreased consumption of proline, leucine, isoleucine, and valine, while increasing the consumption of glutamine, alanine, and hypotaurine (Zhang et al., 2021). Treatment of mitochondrial inhibitors also increases glucose consumption and lactate production, while inhibiting the export of citrate, isocitrate, and β-HB (Zhang et al., 2021). These results highlight the importance of mitochondrial metabolism in facilitating metabolic exchange between the RPE and outer retina.

## 3. Metabolic dysregulation in retinal aging and disease

The retina undergoes significant functional decline throughout the aging process. As age advances, there is a decrease in contrast sensitivity (Skalka, 1980), visual acuity (Gittings and Fozard, 1986), visual field sensitivity (Spry and Johnson, 2001), dark adaptation (Jackson et al., 1999), and ERG response (Birch and Anderson, 1992). In parallel with deteriorating visual function, metabolic dysregulation emerges in the RPE and neural retina. In mice, both the retina and RPE/choroid undergo dramatic changes in the concentration of various sugars, amino acids, and other metabolites with aging (Y. Wang et al., 2018a). Cytochrome *c* oxidase deficiency and mitochondrial DNA deletions increase in aging human photoreceptors (Barron et al., 2001). Mitochondria in aged RPE are less abundant, irregular in shape and size, have less distinct cristae, exhibit reduced glycolytic and mitochondrial capacity (Gouras et al., 2016; He et al., 2010; He and Tombran-Tink, 2010; Rohrer et al., 2016), and exhibit increased mitochondrial DNA (mtDNA) damage (Lin et al., 2011). Levels of NAD^+^ and the NAD^+^ salvage enzyme nicotinamide phosphoribosyltransferase (NAMPT) also decline in the RPE with age (Jadeja et al., 2018; Y. Wang et al., 2018b), potentially impacting ATP production as NAD^+^ is a required cofactor for glycolysis, the LDHB-mediated conversion of lactate to pyruvate, as well as several TCA cycle enzymes. Lipid metabolism is also dysregulated in the aging RPE. Lipid droplets accumulate in the RPE/choroid of aged mice (Yako et al., 2022), and both esterified and unesterified cholesterol accumulates in Bruch’s membrane regions overlying the macula with age (Curcio et al., 2001). Metabolic perturbations are even more pronounced in eye diseases such as AMD.

### 3.1. Age-related macular degeneration

AMD is a progressive retinal disorder characterized by the degeneration of the macula leading to central vision loss, and is estimated to afflict approximately 200 million people globally (Fleckenstein et al., 2021; Jonas et al., 2017). AMD is categorised into two primary forms: non-exudative (dry/atrophic) and exudative (neovascular/wet). The hallmark of early non-exudative AMD is the appearance of sub-RPE deposits (drusen) at the macula. Disease progression is characterized by atrophic changes, with degeneration of the RPE, photoreceptors and choriocapillaris. (Campagne et al., 2014; Rozing et al., 2020). Transition to exudative AMD occurs in 10–15% of affected individuals and is characterised by neovascularisation from the choroid or from within the retina. (Rozing et al., 2020). Although a combination of genetic, life-style, and age-related factors contribute to its aetiology (Fleckenstein et al., 2024), dysregulated retina and RPE metabolism is now also considered an important aspect of AMD pathology (Ferrington et al., 2020; Fisher and Ferrington, 2018; Léveillard et al., 2019; Tan et al., 2020).

#### 3.1.1. Mitochondrial dysfunction

**Fig. 3.**
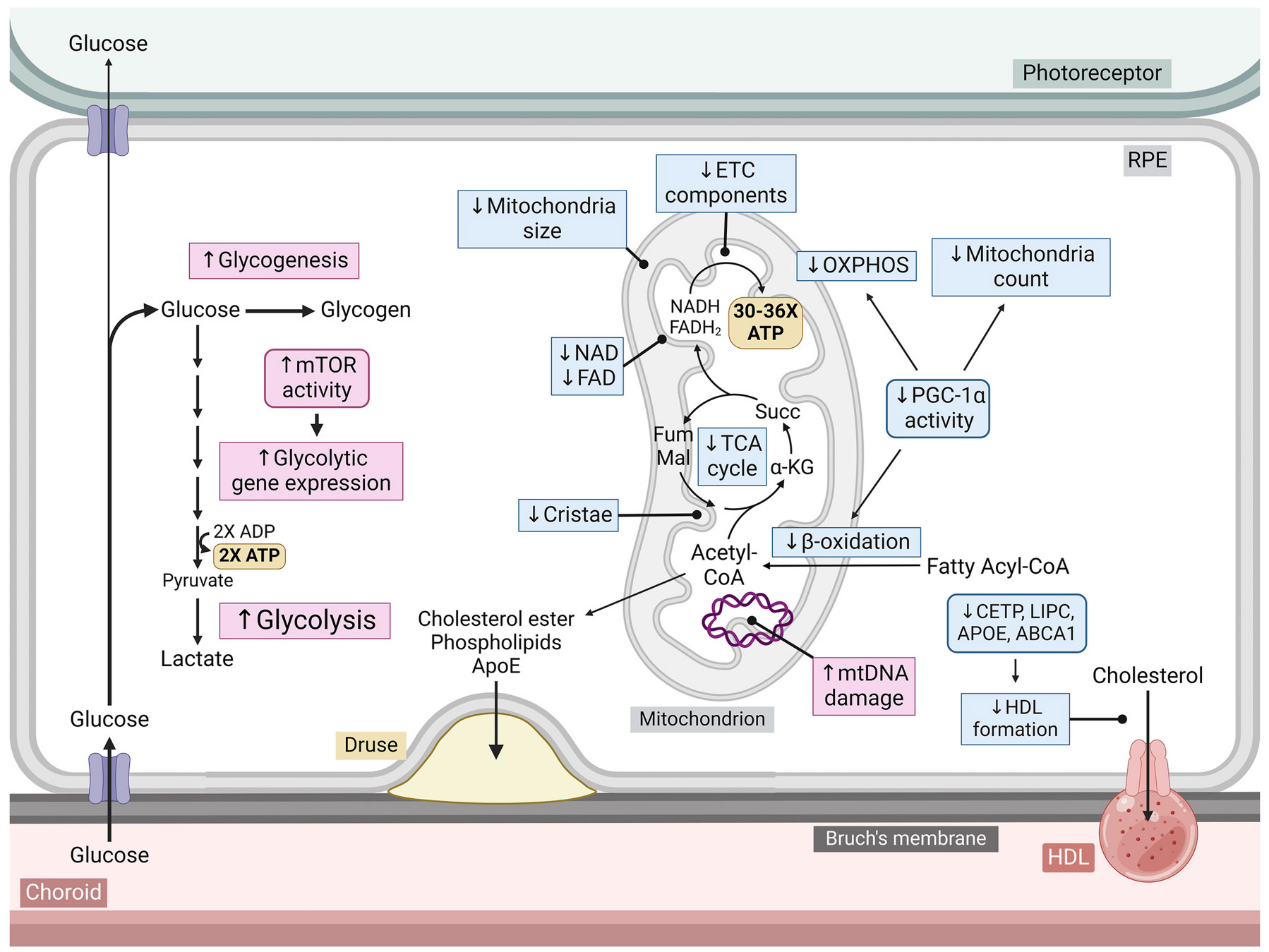
Figure 3. RPE metabolic dysregulation in AMD. Blue boxes indicate decreases and pink boxes indicate increases. Decreased fatty acid oxidation and OXPHOS arise from mtDNA damage, mitochondrial dysfunction, and decreased PGC-1α activity. This causes bioenergetic stress, forcing RPE cells to compensate by increasing glycolysis, potentially restricting glucose supply to the photoreceptors. AMD risk genes contribute to dysregulated cholesterol metabolism, decreasing cholesterol efflux through HDL-like particles and promoting drusen formation. Abbreviations: ABCA1, ATP-binding cassette A1; ADP, adenosine diphosphate; APOE, apolipo-protein E; ATP, adenosine triphosphate; CETP, cholesterol ester transfer protein; ETC, electron transport chain; FADH_2_, flavin adenine dinucleotide; Fum, fumarate; HDL, high-density lipoprotein; LDL, low-density lipoprotein; LIPC, hepatic lipase; Mal, malate; mtDNA, mitochondrial DNA; mTOR, mammalian target of rapamycin; NADH, nicotinamide adenine dinucleotide (reduced); OXPHOS, oxidative phosphorylation; PGC-1α, peroxisome proliferator-activated receptor gamma coactivator 1-alpha; RPE, retinal pigment epithelium; Succ, succinate; TCA, tricarboxylic acid.

Compromised mitochondria accompanied by increased glycolysis in the RPE has been proposed to restrict glucose availability for the photoreceptors, upsetting the metabolic ecosystem and contributing to AMD pathology (Fisher and Ferrington, 2018) (Fig. 3). Electron microscopy of retina and choroid sections shows that RPE mitochondria in AMD patients are fewer in number, have fewer cristae, and have less total area than age-matched controls (Feher et al., 2006). Similarly, cultured RPE cells from donors with AMD exhibited higher numbers of damaged mitochondria than age-matched controls (Golestaneh et al., 2017). Mitochondrial abnormalities are also observed in photoreceptors; in AMD patients, mitochondria shrink and migrate towards the nuclei of cone cells (Litts et al., 2015).

mtDNA damage in the RPE is common in AMD and increases with disease progression (Karunadharma et al., 2010; Lin et al., 2011; Terluk et al., 2015). Notably, RPE mtDNA damage preferentially occurs in regions coding for electron transport chain (ETC) proteins (Terluk et al., 2015). Analysis of the RPE mitochondrial proteome reveals decreased expression of COX VIb and ATP synthase subunits with AMD progression (Nordgaard et al., 2008). Conversely, the transcriptomic and proteomic profile of iPSC-derived RPE cells from donors with geographic atrophy exhibit increased expression of several components involved in the ETC (Senabouth et al., 2022). However, these cells also exhibited impaired maximal respiration, indicating a complex remodelling of mitochondrial OXPHOS.

RPE cells isolated from AMD donors likewise exhibit reduced basal respiration and mitochondrial reserve capacity (Ferrington et al., 2017). Treatment with the autophagy-inducing molecule rapamycin and pyrroloquinoline quinone, a redox factor that induces mitochondrial biogenesis, increases basal and maximal respiration in AMD donor RPE but not those from age-matched controls (Ebeling et al., 2020). Although AMD donor RPE cells exhibit elevated expression of the mitochondrial biogenesis-promoting cofactor PGC-1α (Ferrington et al., 2017), the inactive acetylated form of this protein was recently found to be far more abundant (Zhang et al., 2020). Decreased PGC-1α activity may have pernicious effects as *Pgc-1α+/−* mice exhibit reduced retina/RPE mitochondrial complex I activity, as well as AMD-like phenotypes including a thickened Bruch’s membrane, drusen, oxidative stress, and both RPE and photoreceptor degeneration (Zhang et al., 2018; Zhou et al., 2024).

#### 3.1.2. Glucose metabolism

Compromised ATP production due to mitochondrial dysfunction in AMD may compel the RPE to compensate by upregulating glycolysis. RPE cells isolated from donors with AMD show increased glycolytic ATP production and accumulation of cytoplasmic glycogen (Golestaneh et al., 2017). AMD donor RPE cells also exhibit increased activity of the pro-glycolytic kinase, mammalian target of rapamycin (mTOR) (Duvel et al., 2010; Zhang et al., 2020). Analysis of the RPE proteome suggests that expression of the glycolytic enzyme pyruvate kinase increases with AMD progression (Nordgaard et al., 2006). Similarly, the RPE/choroid tissue of laser-induced CNV mice exhibit upregulated glycolytic gene expression (Liu et al., 2022). iPSC-derived RPE cells from donors with geographic atrophy also show increased expression of glycolytic genes (Senabouth et al., 2022). Contrary to these data, one study found that RPE from AMD donors exhibited decreased basal glycolysis and glycolytic capacity (Ferrington et al., 2017).

#### 3.1.3. NAD and FAD metabolism

Dysregulated metabolism of the metabolic cofactors NAD and FAD may also be involved in AMD. RPE cells from AMD donors contain less total NAD (Zhang et al., 2020), which may contribute to disrupted RPE metabolism as inhibition of the NAD^+^ salvage enzyme NAMPT increases the glycolytic rate in cultured human RPE cells (Jadeja et al., 2020). Moreover, treatment with the NAD^+^ precursor nicotinamide mononucleotide (NMN) increases mitochondrial respiration in RPE cells isolated from some AMD donors but not controls (Ebeling et al., 2020). Treatment with the NAD^+^ precursor, nicotinamide, reduces the expression of inflammatory cytokines and complement factors in a human iPSC model of AMD (Saini et al., 2017), suggesting that NAD^+^ deficiency contributes to AMD pathology. Decreased levels of FAD and its precursor flavin mononucleotide (FMN) are also found in the retinas of several mouse models of retinal degeneration (Sinha et al., 2018). Like NAD^+^, FAD is an essential cofactor in the TCA cycle and the ETC, and its deficiency may contribute to diminished OXPHOS in AMD RPE cells. Moreover, FAD serves as a cofactor in both the proline catabolism and fatty acid oxidation pathways, which are both crucial for maintaining RPE physiology as previously discussed.

#### 3.1.4. Lipid metabolism and drusen

Drusen are dome-shaped deposits of extracellular material that form between the RPE basement membrane and the inner collagenous layer of Bruch’s membrane and are considered a classic hallmark of AMD (Hageman et al., 2001). While they are comprised primarily of protein, lipid components such as esterified cholesterol and phosphatidylcholine account for approximately 40% of druse volume (Wang et al., 2010). The source of drusen components is ambiguous and is likely to originate from a combination of RPE cells, the choroid, and circulation (Bergen et al., 2019; Booij et al., 2010). Nevertheless, the abundant phospholipid and cholesterol components of drusen are highly suggestive of dysregulated lipid metabolism in the RPE.

Polymorphisms in several genes involved in HDL and cholesterol metabolism, including *LIPC, CETP, APOE,* and *ABCA1* have a strong association with AMD (Fritsche et al., 2016). Hepatic lipase (*LIPC)* and cholesterol ester transfer protein (*CETP*) are involved in HDL remodelling and cholesterol transport (Tan et al., 2020; van Leeuwen et al., 2018). *ABC1A* encodes an ATP-binding cassette transporter which transports cholesterol and phospholipids from cells, generating HDL (Liu and Tang, 2012; Storti et al., 2019). RPE-specific knockout of *Abc1a* and its partner *Abcg1* in mice causes RPE lipid accumulation, decreased retinal function, and degeneration of the RPE and photoreceptors (Storti et al., 2019). APOE is primarily a component of lipoproteins, mediating their recognition by cell surface receptors (Huang and Mahley, 2014). APOE is secreted apically and basally by the RPE and is a major protein component of drusen (Ishida et al., 2004; Wang et al., 2010). APOE deficient mice exhibit abnormalities associated with AMD such as a thickened Bruch’s membrane, irregular ERG responses and diminished retinal cell count (Ong et al., 2001). Thus, the composition of drusen, as well as the function of AMD risk genes, highlight the important role of dysregulated cholesterol and lipoprotein metabolism in AMD pathology.

## 4. Inflammation and RPE metabolism

Chronic, non-resolving inflammation is a pervasive feature of aging tissues and a fundamental element of many diseases including atherosclerosis, rheumatoid arthritis and pulmonary fibrosis (Nathan and Ding, 2010). ‘Inflammaging’ is thought to stem from diverse causes, from damage and pathogen associated molecular patterns (DAMPs and PAMPs) through to gut microbiota (Franceschi et al., 2018). Importantly, chronic inflammation is also a common feature of the aged retina. For example, the transcriptional profile of both the retina and RPE/choroid change with age, favouring the expression of complement components, immune modulators, and cytokines - key drivers of inflammation (M. Chen et al., 2010a; Ida et al., 2004). Chronic inflammation in the retina is also intimately linked with diseases such as AMD (Ambati et al., 2013). This section will outline the features of the aging RPE that contribute to chronic local inflammation and discuss key cytokines involved in this process, with a focus on their predicted impact on RPE metabolism.

### 4.1. Sources of retinal inflammation during aging and disease

#### 4.1.1. Damage-associated molecular patterns

DAMPs are molecules commonly released by cells into the extracellular space in response to stress or injury, including proteins, DNA, and metabolites that activate innate immune cells and trigger sterile inflammation (Chen and Nuñez, 2010). Most DAMPs are found ubiquitously across cell types and include the protein high mobility group box 1 (HMGB1), S100/calgranulin peptides, heat shock proteins (HSPs), DNA, uric acid, and ATP (Mahaling et al., 2022). DAMPs are also formed through the modification of extracellular matrix components including hyaluronan, collagen, and heparan sulphate (Qin and Rodrigues, 2008). DAMPs in the retina and RPE are associated with aging, as well as with retinal disorders such as AMD (Mahaling et al., 2022). The RPE expresses toll-like receptors (TLRs) (Kumar et al., 2004) and RAGE which recognise and respond to DAMPs, triggering a proinflammatory signalling cascade (Ott et al., 2014; Tsan and Gao, 2004).

#### 4.1.2. Basal deposits and drusen

Basal laminar deposits are extracellular deposits that form between the RPE and the RPE basement membrane (Sura et al., 2020), consisting primarily of excess basement membrane components such as type IV collagen, heparin sulphate proteoglycan (HSPG), and laminin (Van der Schaft et al., 1994). AGEs are also found in basal laminar deposits and their levels increase with age (Farboud et al., 1999). Significantly more basal laminar deposits are found in AMD patients compared to age-matched controls (Spraul et al., 1996), and have been proposed to define the threshold for the disease (Sarks et al., 2007). Matrix metalloproteinases (MMPs) are found in the basal laminar deposits of CNV patients (Lommatzsch et al., 2008), as well as the complement factors C3 and C5b-9, suggesting they may contribute to inflammation in AMD.

Drusen also act as inflammatory nodes, home to inflammatory mediators such as vitronectin, amyloid P component, C-reactive protein (CRP), α1-antichymotrypsin, fibrinogen, and a range of complement components (Anderson et al., 2002; Mullins et al., 2000; Wang et al., 2010). Moreover, amyloid-β, another major constituent of drusen, stimulates the expression of cytokines such as TNFα and IL-1β from cultured macrophages (Wang et al., 2009) and the RPE/choroid *in vivo* (Liu et al., 2013).

#### 4.1.3. Immune cell recruitment

In a healthy eye, the outer retina and sub-retinal space is immune privileged and devoid of immune cells except for inactive microglia (Murenu et al., 2022; Streilein, 2003). In contrast, the choroid is populated by resident immune cells such as dendritic cells and macrophages (Kumar et al., 2014). During aging, increased drusen and basal laminar deposits are associated with recruitment and changes in the immunophenotype of resident choroidal macrophages (Cherepanoff et al., 2010). Aged mouse RPE/choroid tissues exhibit upregulated expression of genes involved in leukocyte extravasation signalling such as *Ccl2,* which encodes monocyte chemoattractant protein 1 (MCP1) (Chen et al., 2008). Immune cell recruitment to the RPE/choroid also becomes more pronounced in retinal disease, with activated microglia and macrophages accumulating in the subretinal space in both AMD and the hereditary retinal dystrophy, retinitis pigmentosa (Combadière et al., 2007; Eandi et al., 2016; Gupta et al., 2003; Lad et al., 2015). These activated microglia and macrophages secrete key cytokines including TNFα, IL-6, and IL-1β, further perpetuating inflammatory conditions (Smith et al., 2012; Wynn et al., 2013).

#### 4.1.4. RPE senescence

Thought to be a tumour-supressing mechanism, some mitotic and post-mitotic cells acquire a senescence phenotype in response to DNA damage, chromatin perturbations, abnormal mitogenic signals, and other stressors (Campisi and D’Adda Di Fagagna, 2007; Lee et al., 2021). Cellular senescence is characterised by apoptosis resistance, growth arrest, altered gene expression profiles, and a senescence-associated secretory phenotype (SASP) (Campisi and D’Adda Di Fagagna, 2007). The SASP involves the secretion of a range of cytokines, chemokines, and other factors including MMPs, growth factors, and IGF binding proteins (IGFBPs) (Davalos et al., 2010). During aging and in AMD, RPE cells acquire a SASP (Chaum et al., 2015; Lee et al., 2021), driven by drusen components (Cao et al., 2013), oxidative stress (Zhu et al., 2009), metabolic dysregulation (Y. Chen et al., 2010b), and other factors (Lee et al., 2021). Senescent RPE cells secrete numerous inflammatory cytokines and other proteins, including MMP-9, MCP-1, IL-6, TNFα, and TGF-β (Lee et al., 2021).

### 4.2. Inflammatory cytokines

Chronic inflammatory conditions in the aging and diseased retina lead to the secretion of an ensemble of cytokines from infiltrating immune cells and the RPE itself. This section will focus on the four most common and well characterised cytokines associated with age-related inflammation and disease, TNFα, TGF-β, IL-6 and IL-1β. These cytokines bind specific receptors and activate both shared and distinct signalling pathways in the RPE. Although these pathways are mostly well characterised, emerging evidence from diverse *in vitro* models have implicated these cytokines in mediating broad cellular responses including metabolic regulation. Given their broad effects on glucose, mitochondrial, lipid, and amino acid metabolism in other tissues, it is likely that all four of these cytokines also mediate significant metabolic changes in the RPE. This supports the notion that inflammation can disrupt the delicate metabolic ecosystem of the retina by influencing RPE metabolism in aging and disease.

#### 4.2.1. Tumour necrosis factor alpha (TNFα)

Originally identified for its anti-tumour properties, TNFα has become one of the most extensively studied cytokines, with roles ranging from mediating inflammation to regulating cell survival and differentiation (Brenner et al., 2015). Produced predominantly by macrophages, TNFα is also expressed in a variety of other cells including neurons, fibroblasts, and endothelial cells (Wajant et al., 2003). Both membrane-bound and soluble TNFα typically exert their biological functions through the receptors TNF-R1 and TNF-R2 (Brenner et al., 2015; Wajant et al., 2003). TNF-R2 expression is tightly regulated and mostly restricted to the cells of the immune system, while TNF-R1 is ubiquitously expressed across tissues (Aggarwal, 2003; Wajant et al., 2003). Notably, the RPE expresses both TNF-R1 and TNF-R2 (Kociok et al., 1998; Shi et al., 2008).

##### 4.2.1.1. TNFα signalling in the eye.

TNFα is a common marker of chronic inflammation in aging tissues (BRUUNSGAARD et al., 2003; Singh and Newman, 2011) and also increases with age in the retina (Xu et al., 2009). It is elevated in a range of eye diseases, including proliferative diabetic retinopathy (PDR) (Demircan et al., 2006; Zhou et al., 2012), glaucoma (Yuan and Neufeld, 2000), as well as wet AMD (Cousins et al., 2004; Oh et al., 1999). Amyloid-β, a common component of drusen, stimulates expression of TNFα in the RPE/choroid (Liu et al., 2013), providing a potential driver of TNFα expression in AMD. PGC-1α suppresses TNFα expression but, as previously discussed, is largely inactive in the RPE of AMD donors (Eisele et al., 2013; Zhang et al., 2020). TNFα elicits transcriptional responses in numerous RPE models, including ARPE-19 cells (Korthagen et al., 2015), hfRPE cells (Shu et al., 2022), and human primary RPE cells (Yang et al., 2005), where it predominantly upregulates genes involved in cell survival, signal transduction, and immune response.

##### 4.2.1.2. TNFα and metabolism

###### 4.2.1.2.1. TNFα and glucose metabolism.

The role of TNFα as a mediator of insulin resistance (IR) is well established and is linked to several diseases (Sethi and Hotamisligil, 1999). TNFα primarily contributes to IR in skeletal muscle cells (Steinberg et al., 2006), and adipocytes (Cawthorn and Sethi, 2008), where it inhibits insulin receptor signalling and expression of the glucose transporter, GLUT4 (Hotamisligil et al., 1994; Rotter et al., 2003; Stephens and Pekala, 1991). However, TNFα administration also been reported to cause a rapid decrease in blood glucose and hepatic glycogen in mice (Mahony and Tisdale, 1990).

**Fig. 4.**
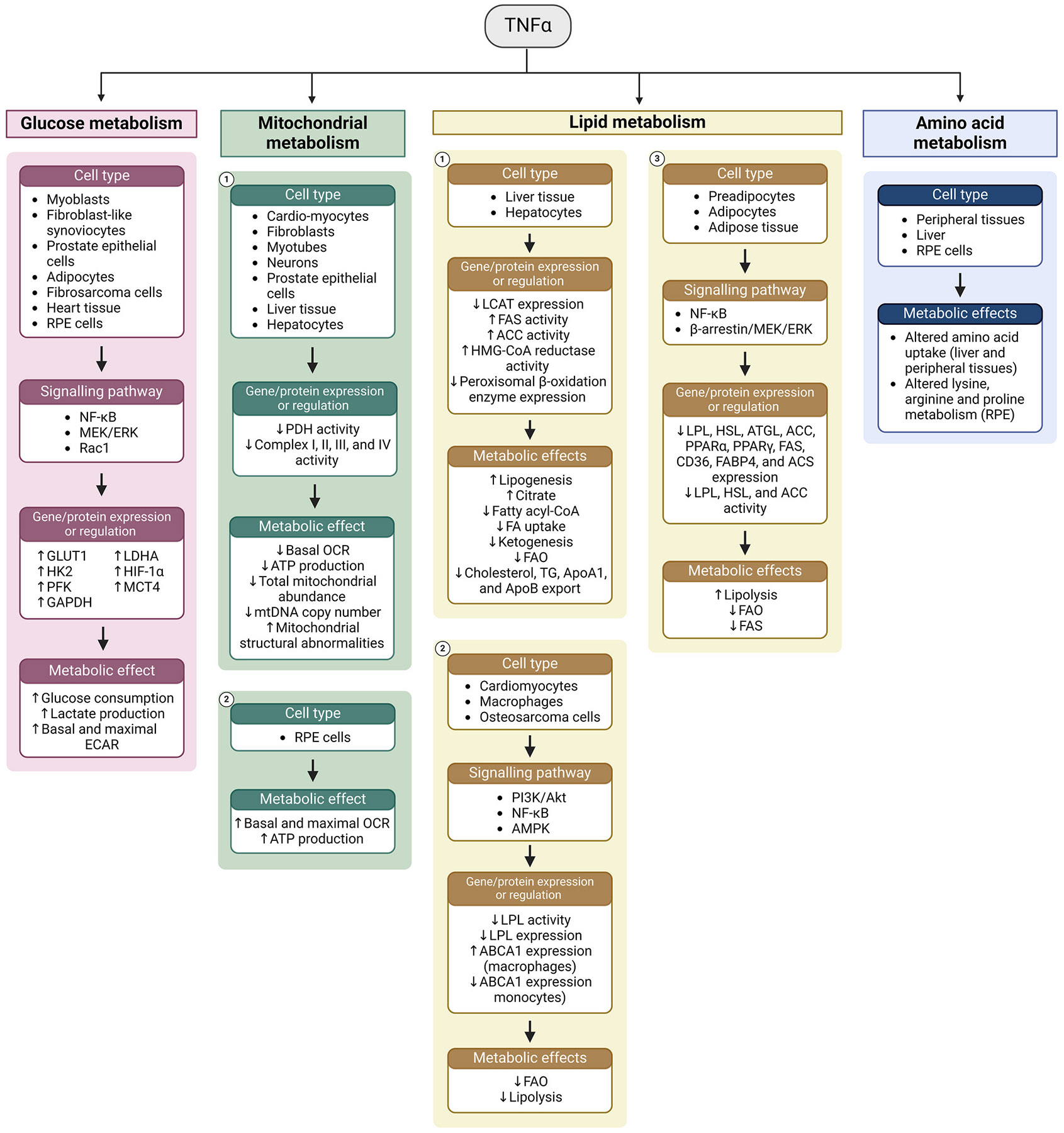
Figure 4. The effects of TNFα on metabolism. Summary of the effects of TNFα on glucose (purple), mitochondrial (green), lipid (beige), and amino acid (blue) metabolism in various cell types. Numbers in top left corner of boxes indicate differing effects depending on cell type. ↑ indicates an increase and ↓ indicates a decrease.

TNFα promotes glycolysis and induces glycolytic gene expression in a range of cell types (Fig. 4, Table 1). TNFα treatment increases the expression of *Slc2a1* (GLUT1), *Slc16a3* (MCT4), *Hk2* (hexokinase 2), *Pfk* (phosphofructokinase), *Ldha,* and the glycolytic transcription factor *Hif1a* in mouse myoblasts (Remels et al., 2015). Similarly, TNFα induces *SLC2A1,* the PFK liver isoform (*PFKL*), and *HIF1A* expression in human fibroblast-like synoviocytes (Koedderitzsch et al., 2021), *Ldha* in porcine Sertoli cells (Boussouar and Benahmen, 1999), and GLUT1 protein expression in human prostate epithelial cells (Vaughan et al., 2013). In addition to increasing *Pfk* and glyceraldehyde 3-phosphate dehydrogenase (*Gapdh*) expression, TNFα promotes glucose consumption and lactate production in mouse fibrosarcoma cells (Sánchez-Alcázar et al., 2003), myoblasts (Remels et al., 2015), and pancreatic cancer cells (M. Liu et al., 2016b). It also promotes lactate production in primary rat adipocytes (Porter et al., 2002), and isolated perfused rat hearts (Hülsmann and Dubelaar, 1988), while increasing basal and maximal extracellular acidification rate (ECAR), a measure of glycolysis, in fibroblast-like synoviocytes (Koedderitzsch et al., 2021).

The transcription factor nuclear factor kappa-light-chain-enhancer of activated B cells (NF-κB) is perhaps the most potent and well characterised downstream target of TNFα, orchestrating the expression of genes involved in inflammation, adhesion, cell cycle regulation, and survival (Kageyama et al., 2018). NF-κB binds to the *MMP-1, -3*, and *-9* promoters which, as discussed below, can regulate metabolism through growth factor signalling pathways (Fanjul-Fernández et al., 2010). NF-κB also regulates glycolysis directly by upregulating the glycolytic enzymes HK2 (Londhe et al., 2018) and GLUT3 (Kawauchi et al., 2008; Zha et al., 2015). Transcription of the glycolytic transcription factor HIF-1α is also upregulated by NF-κB (T. Yoshida et al., 2013a) which mediates the effects of TNFα on mouse myoblast glucose metabolism (Remels et al., 2015). Moreover, NF-κB induction promotes glycolytic ATP production, glycogen synthesis, and suppression of PPARγ in ARPE-19 cells (Guo et al., 2022). The effects of TNFα on glucose metabolism are abolished with NF-κB inhibition in hfRPE cells (Shu et al., 2022), but not in ARPE-19 cells (Hansman et al., 2024). Thus, the role of NF-κB in regulating RPE metabolism *in vivo* remains unclear.

TNF-R1 activation by TNFα also activates mitogen-activated protein kinase (MAPK) pathways such as MEK/ERK and c-Jun N-terminal kinase (JNK) (Sabio and Davis, 2014), both of which regulate glucose metabolism, either through activation of glycolytic genes or through modification of metabolic enzymes (Papa et al., 2019). Moreover, TNFα signalling also activates the Rho-like small GTPase Rac1 (Papaharalambus et al., 2005), which can increase glycolysis by rear-ranging F-actin structures, increasing mobilisation of the glycolytic enzyme, aldolase (Hu et al., 2016).

###### 4.2.1.2.2. TNFα and mitochondrial metabolism.

In most tissues, TNFα has been reported to have an inhibitory effect on mitochondrial respiration. TNFα suppresses the basal OCR in rat cardiomyocytes (Zell et al., 1997), mouse fibroblasts (Schulze-Osthoff et al., 1992), myotubes (Tse et al., 2017), and neurons (Doll et al., 2015), as well as in human prostate epithelial cells (Vaughan et al., 2013). The suppressive effect of TNFα on respiration is mediated by inhibition of pyruvate dehydrogenase (PDH) (Zell et al., 1997) and respiratory complexes I (Zell et al., 1997), II (Zell et al., 1997) and III (Suematsu et al., 2003) in primary rat cardiomyocytes, and through tyrosine phosphorylation of complex IV in the mouse and bovine liver (Samavati et al., 2008). Moreover, treatment of human chondrocytes with TNFα results in decreased complex I activity, mitochondrial membrane potential, and ATP production (López-Armada et al., 2006).

Mitochondrial abnormalities may also mediate the effects of TNFα on respiration. TNFα decreases total mitochondrial abundance in human prostate epithelial cells (Vaughan et al., 2013), induces structural abnormalities in mouse fibroblasts (Schulze-Osthoff et al., 1992), and decreases mtDNA copy number in rat cardiomyocytes (Suematsu et al., 2003). The effects of TNFα on OCR in mouse hepatocytes (Kastl et al., 2014), liver tissue (Samavati et al., 2008), and neurons (Doll et al., 2015) are accompanied by decreases in ATP production. Conversely, TNFα increases both OCR and ATP production in hfRPE cells (Shu et al., 2022), and increases mitochondrial reserve capacity in differentiated ARPE-19 cells (Hansman et al., 2024). The contrast between the effects of TNFα on respiration in the RPE compared to other tissues is compelling and warrants further investigation.

###### 4.2.1.2.3. TNFα and lipid metabolism.

TNFα has potent effects on systemic lipid metabolism, broadly promoting lipolysis while decreasing circulating cholesterol. TNFα administration increases serum triglyceride (TG) levels in mice (Feingold et al., 1994; Memon et al., 1993), and plasma glycerol, TG, and FFA concentrations in rats (Feingold et al., 1989a, 1990; Grunfeld and Feingold, 1995). Similarly, TNFα increases plasma triacylglycerol (TAG) levels in cynomolgus monkeys (Ettinger et al., 1992), and both plasma glycerol and FFA in humans (Plomgaard et al., 2005). Circulating total cholesterol and HDL-cholesterol are also decreased with TNFα administration in humans (Popa et al., 2007), with systemic anti-TNFα therapy similarly increasing circulating HDL-cholesterol levels (Popa et al., 2005; Vis et al., 2005). In Syrian hamsters, TNFα treatment suppresses expression of the cholesterol transporter CETP in a range of tissues including kidney, muscle and heart (Hardardóttir et al., 1996). Moreover, activity of the enzyme lecithin-cholesterol acyltransferase (LCAT), which converts free cholesterol into cholesterol ester, is decreased with TNFα treatment in the plasma of both Syrian hamsters (Ly et al., 1995) and cynomolgus monkeys (Ettinger et al., 1990, 1992).

TNFα promotes lipolysis in a range of *in vitro* adipocyte models. It increases glycerol release from murine adipocytes (Jack et al., 2022; Kawakami et al., 1987; Sethi et al., 2000; Souza et al., 1998a; Souza et al., 1998b), rat adipocytes (Green et al., 1994), and rat adipose tissue explants (Porter et al., 2002). Interestingly, the lipolytic effect of TNFα occurs despite its inhibitory effect on lipolytic enzymes. In mouse 3T3-L1 adipocytes, TNFα inhibits lipoprotein lipase (LPL) (Beutler and Cerami, 1985; Kawakami et al., 1987; Ogawa et al., 1989; Price et al., 1986b; Sumida et al., 1990) and hormone sensitive lipase (HSL) (Sumida et al., 1990) activity, and decreases HSL (Jack et al., 2022; Sumida et al., 1990; Tamori et al., 2002), LPL (Jack et al., 2022; Tamori et al., 2002), and adipose triglyceride lipase (ATGL) (Kralisch et al., 2005) gene expression. Likewise, TNFα administration decreases adipose LPL activity in mice (Feingold et al., 1994) and adipose LPL expression in rats (Ruan et al., 2002b). The paradox concerning why TNFα increases lipolysis in adipose cells while decreasing lipolytic gene expression is unresolved and the subject of ongoing research (Cawthorn and Sethi, 2008; Jack et al., 2022).

TNFα also decreases the activity and expression of genes involved in fatty acid oxidation and synthesis in adipocytes. TNFα treatment inhibits acetyl-CoA carboxylase (ACC) activity and expression in mouse preadipocytes (Pape and Kim, 1988), and decreases PPARγ expression in mature rat adipocytes (Tanaka et al., 1999). Moreover, in 3T3-L1 adipocytes, TNFα decreases the expression of acetyl-CoA synthetase (ACS) (Tamori et al., 2002), fatty acid synthase (FAS) (Jack et al., 2022), PPARα (Jack et al., 2022), PPARγ (Jack et al., 2022; Rotter et al., 2003; Xing et al., 1997; Zhang et al., 1996), and the fatty acid transporters CD36 (Tamori et al., 2002) and fatty acid binding protein 4 (FABP4) (Jack et al., 2022). Treatment of 3T3-L1 cells with a PPARγ agonist blocks TNFα-induced lipolytic activity (Sandler et al., 1990), offering a mechanism through which TNFα induces lipolysis despite repressing lipolytic genes. TNFα-induced lipolysis in human adipocytes (Laurencikiene et al., 2007) and repression of PPARγ, HSL and FAS in mouse adipocytes (Ruan et al., 2002a) is mediated by the transcription factor NF-κB. MEK1 inhibition also prevents TNFα-induced lipolysis in human adipocytes (Rydén et al., 2002) and preadipocytes (Rydén et al., 2002). Moreover, TNFα-induced lipolysis was found to be partially dependent on the β-arrestin-ERK pathway in 3T3-L1 cells (Kawamata et al., 2007).

In contrast to adipocytes, TNFα promotes fatty acid synthesis in hepatocytes. TNFα administration increases hepatic FAS and ACC activity in rats (Grunfeld et al., 1988) and increases hepatic citrate levels and lipogenesis in mice (Grunfeld et al., 1990). On the other hand, TNFα may inhibit hepatic FAO. TNFα administration in rats decreases the hepatic expression of peroxisomal β-oxidation enzymes (Beier et al., 1992), hepatic fatty acyl-CoA levels (Grunfeld et al., 1988), and fatty acid uptake and β-HB production in isolated perfused rat livers (De Bandt et al., 1994). Moreover, TNFα treatment inhibits palmitate oxidation in cultured rat hepatocytes (Nachiappan et al., 1994). TNFα may promote cholesterol synthesis while having an inhibitory effect on lipoprotein biogenesis. In mice, TNFα administration increases activity of HMGCR, the rate-limiting enzyme in cholesterol synthesis (Memon et al., 1993). Likewise, hepatic HMGCR expression is increased following TNFα treatment in rats (Ruan et al., 2002b). TNFα decreases hepatic LCAT expression in Syrian hamsters *in vivo* (Ly et al., 1995), as well as in HepG2 (Ettinger et al., 1994) and H35 (Ly et al., 1995) hepatoma cells. This is accompanied by decreased accumulation of TG, cholesterol, and the apolipoproteins ApoA1 and ApoB in the medium of HepG2 cells (Ettinger et al., 1994). Thus, TNFα elicits contrasting effects on hepatic cholesterol metabolism depending on experimental model.

Outside of adipose and liver tissue, TNFα broadly inhibits lipolysis and FAO. It inhibits LPL activity in primary rat cardiomyocytes (Hülsmann and Dubelaar, 1988), mouse macrophages (Tengku-Muhammad et al., 1998, 1999), and human osteosarcoma cells (Sakayama et al., 1996). Inhibition of LPL expression and activity with TNFα treatment in J774.2 mouse macrophages is mediated by phosphoinositide 3-kinase (PI3K) signalling (Tengku-Muhammad et al., 1999). TNFα also inhibits expression of the fatty acid transporter ABCA1 in murine macrophages and human monocytes (Khovidhunkit et al., 2003; Mei et al., 2007; Voloshyna et al., 2014), but has also been reported to induce ABCA1 in mouse peritoneal macrophages through NF-κB (Gerbod-Giannone et al., 2006). TNFα suppresses FAO in human osteosarcoma cells (Sakayama et al., 1996), mouse myotubes (Tse et al., 2017), and mouse muscle tissue (Steinberg et al., 2006). TNFα-induced suppression of FAO in skeletal muscle involves suppression of AMP-activated protein kinase (AMPK) signalling (Steinberg et al., 2006; Tse et al., 2017). While its effects on lipid metabolism vary depending on cell type, TNFα consistently inhibits FAO in adipocytes, hepatocytes, myocytes, macrophages, and other cells. Whether TNFα elicits similar effects on RPE FAO, or whether it perturbs other aspects of RPE lipid metabolism, remains an open question.

###### 4.2.1.2.4. TNFα and amino acid metabolism.

TNFα administration influences systemic amino acid metabolism, increasing the uptake of serine, glycine, alanine, proline, tryptophan, and histidine from circulation in rats (Argiles et al., 1989). Another study observed an increase in whole body leucine turnover, oxidation, and clearance and increased plasma concentrations of tryptophan and ornithine with TNFα infusion in rats (Holeček et al., 1997). TNFα has particularly potent effects on hepatic amino acid metabolism. TNFα administration has been reported to increase the plasma membrane vesicle-mediated uptake of cysteine and glutamine in rat livers (Pacitti et al., 1993), as well as increase the hepatic uptake of alanine, serine, glutamate, arginine, proline, lysine and threonine, while decreasing uptake of leucine, isoleucine, and valine (Argiles and Lopez-Soriano, 1990). Studies using isolated perfused rat livers suggest that TNFα promotes the hepatic uptake of phenylalanine, tyrosine, valine, and isoleucine, while decreasing uptake of alanine, asparagine, glutamine (De Bandt et al., 1994; Holeček et al., 1997). While these data contradict somewhat regarding which specific amino acids are trafficked by the liver and peripheral tissues, these discrepancies may reflect shifting systemic metabolic conditions resulting from feeding time, circadian rhythm, and other factors. In hfRPE cells, metabolic pathway analysis identifies lysine degradation and arginine and proline metabolism pathways as being enriched following TNFα treatment (Ng et al., 2023). Given the emerging significance of amino acid metabolism in maintaining the metabolic ecosystem, the effects of TNFα on RPE amino acid uptake and metabolism warrants further investigation.

#### 4.2.2. Transforming growth factor beta (TGF-β)

Transforming growth factor beta 1–3 (TGF-β1-3) are multifunctional cytokines that play crucial roles in immune regulation, development and tissue homeostasis (Batlle and Massague, 2019). Secreted TGF-β is sequestered by the large latent complex (LLC), which serves as a TGF-β reservoir, releasing active TGF-β in response to extracellular matrix (ECM) perturbations such as low pH, reactive oxygen species (ROS), and stress-associated proteins including MMP-2 and MMP-9 (Xu et al., 2018). Once released, active TGF-β ligands bind and unite the TGF-β receptor types I and II, initiating a downstream signalling cascade (Shi and Massagué, 2003).

The TGF-β receptor complex elicits downstream responses through both canonical and non-canonical signalling pathways. In the canonical pathway, the type I receptor phosphorylates the receptor-activated Smad proteins (R-Smads), Smad2 and Smad3 (Xu et al., 2018), allowing for the formation of heterotrimers with Smad4, and binding to the Smad binding element (SBE) (Zawel et al., 1998). Non-canonical (Smad-independent) signalling involves a diverse range of pathways including Wnt/β-catenin, NF-κB, Notch, PI3K/AKT, and MEK/ERK (Kubiczkova et al., 2012).

##### 4.2.2.1. TGF-β signalling in the eye.

TGF-β2 is the predominant TGF-β isoform in the eye, and is approximately 10 times more abundant in the RPE/choroid than in the neural retina (Pfeffer et al., 1994; Pfeiffer et al., 1997; Tanihara et al., 1993). Both TGF-β1 and TGF-β2 maintain retinal homeostasis by regulating photoreceptor OS phagocytosis in the RPE (Sheu et al., 1994). Moreover, TGF-β2 also promotes RPE cell survival when cultured on aged and AMD Bruch’s membrane (Sugino et al., 2016). Conversely, TGF-β is a key regulator of epithelial to mesenchymal transition (EMT) and promotes fibrotic membrane formation in proliferative vitreoretinopathy (PVR) (Connor et al., 1989; Xu et al., 2018). TGF-β is also associated with AMD pathology, showing higher abundance in the aqueous humour (Tosi et al., 2017) and RPE/choroid (Kliffen et al., 1997) of AMD patients. All three TGF-β isoforms induce secretion of vascular endothelial growth factor (VEGF), a key driver of CNV, in cultured RPE cells(Nagineni et al., 2003). Moreover, the AMD risk gene *HTRA1* facilitates TGF-β signalling by releasing it from the LLC (Beaufort et al., 2014). The role of TGF-β in wet AMD remains controversial however, as TGF-β exhibits both pro- and anti-angiogenic effects in AMD models depending on disease stage, dose, signalling pathway target, and animal model (Tosi et al., 2018; K. Wang et al., 2018c).

As discussed below, MMPs increase in abundance and activity in the aging Bruch’s membrane. This, coupled with the increases in ROS and low pH associated with chronic inflammation (Mittal et al., 2014; Rajamäki et al., 2013), may provoke heightened release of TGF-β by the LLC. Senescent microglia and infiltrating macrophages secrete TGF-β (Lee et al., 2021), and cultured RPE cells have been shown to convert CD4^+^ T cells into TGF-β-producing regulatory T (Treg) cells (Sugita et al., 2008). TGF-β1 and TGF-β2 secretion in cultured human RPE cells is also inducible by oxidative stress (Yu et al., 2009). Moreover, plasma concentrations of active TGF-β1 increase markedly in elderly humans (Forsey et al., 2003). Thus, a combination of endogenous and exogenous sources, as well as elevated LLC release, may contribute to increased TGF-β signalling in the aging and diseased RPE.

##### 4.2.2.2. TGF-β and metabolism

###### 4.2.2.2.1. TGF-β and glucose metabolism.

**Fig. 5.**
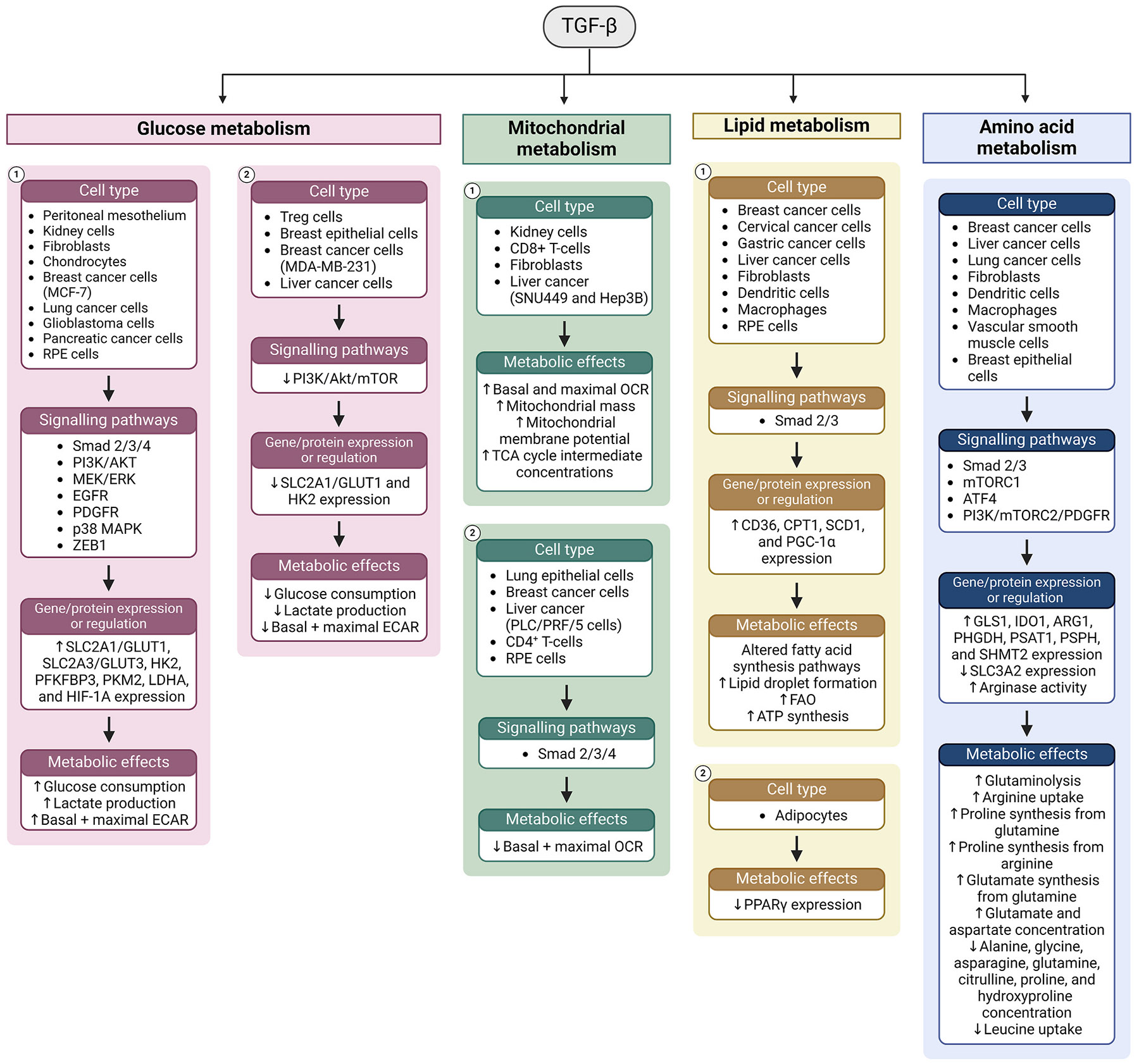
Figure 5. The effects of TGF-β on metabolism. Summary of the effects of TGF-β on glucose (purple), mitochondrial (green), lipid (beige), and amino acid (blue) metabolism in various cell types. Numbers in top left corner of boxes indicate differing effects depending on cell type. ↑ indicates an increase and ↓ indicates a decrease.

TGF-β1 has pleiotropic effects on glycolytic gene expression depending on cell type (Fig. 5, Table 2). TGF-β1 treatment increases *SLC2A1, LDHA,* pyruvate dehydrogenase kinase 1 (*PDK1*), and *HIF1A* expression in human peritoneal mesothelial cells (Young et al., 2014). TGF-β1 also induces expression of GLUT1 in rat mesangial cells (Inoki, 1999), mouse fibroblasts (Kitagawa et al., 1991), MCF-7 breast cancer cells (Li et al., 2013), and human articular chondrocytes (C. Wang et al., 2018a). On the other hand, TGF-β1 reportedly reduces GLUT1 mRNA and protein expression in murine thymic-derived Treg cells (Chen et al., 2020; Priyadharshini et al., 2018), and mammary epithelial cells (Nilchian et al., 2020), as well as human breast cancer (Nilchian et al., 2020) and glioblastoma (Rodríguez-García et al., 2017) cell lines. HK2 is induced by TGF-β1 in human chondrocytes (C. Wang et al., 2018c), glioblastoma cells (Rodríguez-García et al., 2017), and in multiple mouse and human fibroblast cell lines (Yin et al., 2019), but is repressed in murine thymic Treg cells (Chen et al., 2020; Priyadharshini et al., 2018). Moreover, TGF-β1 promotes HIF-1α protein accumulation in human lung fibroblasts (Bernard et al., 2018) and induces pyruvate kinase muscle isozyme 2 (PKM2) expression human lung adenocarcinoma epithelial cells (Y. Liu et al., 2016a). In human fibroblasts (Selvarajah et al., 2019), glioblastoma (Rodríguez-García et al., 2017), and pancreatic ductal carcinoma (Yalcin et al., 2017) cells, TGF-β1 treatment increases expression of 6-phosphofructo-2-Kinase/fructose-2,6-bisphosphatase 3 (PFKFB3), the enzyme responsible for producing fructose-2, 6-bisphosphate, a potent allosteric activator of PFK, a rate-limiting enzyme of glycolysis (Ros and Schulze, 2013). Due to the predominance of TGF-β2 in the retina, recent investigations have focused on the effects of this isoform on glycolytic gene expression in the RPE. TGF-β2 has been shown to promote expression of *SLC2A1* (Hansman et al., 2024), *SLC2A3* (Shu et al., 2021), and *LDHA* (Hansman et al., 2024) in ARPE-19 cells. However, given that TGF-β has been reported to both promote gene expression and decrease protein expression of glucose transporters in breast cancer cells, further studies are warranted to investigate its effects on protein expression.

The cell type-dependent effects of TGF-β on glycolytic gene expression are reflected by its effects on glucose consumption and lactate production. TGF-β1 treatment promotes both glucose consumption and lactate production in mouse fibroblasts (Kitagawa et al., 1991; Schwörer et al., 2020; Yin et al., 2019), human peritoneal mesothelial cells (Young et al., 2014), articular chondrocytes (C. Wang et al., 2018c), as well as pancreatic (M. Liu et al., 2016b; Yalcin et al., 2017), glioblastoma (Rodríguez-García et al., 2017), and breast cancer cells (Lee et al., 2016; Li et al., 2013). Additionally, TGF-β1 promotes glucose consumption in rat mesangial cells (Inoki, 1999), lactate production in rat (Boerner et al., 1985) and human (Selvarajah et al., 2019) fibroblasts, and increases both basal and maximal ECAR in mouse podocytes (Abe et al., 2013). Conversely, TGF-β1 reduces glucose uptake in mouse mammary epithelial cells and MDA-MB-231 triple negative breast cancer cells (Nilchian et al., 2020). Moreover, TGF-β1 inhibits lactate production in murine thymic Treg cells (Chen et al., 2020), and decreases basal and maximal ECAR in thymic Treg cells (Priyadharshini et al., 2018) and several hepatocellular carcinoma cell lines (Soukupova et al., 2017, 2021). In vitro models suggest that TGF-β2 promotes glycolysis in the RPE. TGF-β2 treatment increases glucose consumption in primary human RPE cells and rat eyecups (Hansman et al., 2024). In ARPE-19 cells, TGF-β2 increases glucose consumption (Hansman et al., 2024; Shu et al., 2021), lactate production (Hansman et al., 2024), and glycolytic reserve capacity (Shu et al., 2021).

The Smad signalling pathway is responsible for mediating many of the diverse metabolic effects induced by TGF-β. Smad4 interacts with PKM2 to modulate lactate production in mouse podocytes and Smad4 deficiency in these cells elevates the basal and maximal ECAR (J. Li et al., 2020a). Non-canonical TGF-β signalling activates similar glycolysis-promoting pathways to TNFα, such as MEK/ERK (Lee et al., 2007), JNK (Yamashita et al., 2008), and NF-κB (Arsura et al., 2003). TGF-β signalling also activates PI3K/AKT and the downstream mTORC1/2 (Zhang et al., 2013). AKT regulates metabolism by directly phosphorylating metabolic enzymes (Cross et al., 1995; Deprez et al., 1997; Lee et al., 2017; Roberts et al., 2013), promoting GLUT4 membrane trafficking (Sakamoto and Holman, 2008), and activating mTORC1, which promotes translation of the glycolytic transcription factors HIF-1α and c-Myc (Duvel et al., 2010). Both canonical and non-canonical pathways mediate TGF-β1-induced HK2 induction in mouse fibroblasts, where its effect is dependent on Smad2/3, PI3K, ERK, PDGFR, and EGFR (Yin et al., 2019). Similarly, TGF-β1-induced PFKFB3 expression in glioblastoma cells is prevented by inhibition of Smad3, PI3K, and p38 MAPK (Rodríguez-García et al., 2017), while TGF-β1-induced *SLC2A1* expression and increased basal ECAR are diminished by pan-mTOR inhibition in human fibroblasts (Selvarajah et al., 2019). Conversely, TGF-β1 inhibits glycolysis in murine thymic Treg cells by suppressing PI3K/mTOR signalling (Priyadharshini et al., 2018). Lastly, TGF-β2-induced GLUT3 expression is mediated by ZEB1 (Masin et al., 2014), an EMT-associated transcription factor activated by PI3K/AKT, MEK/ERK, and Smad signalling (Drápela et al., 2020).

###### 4.2.2.2.2. TGF-β and mitochondrial metabolism.

As with glycolysis, the effects of TGF-β on mitochondrial respiration are mixed depending on cell type. While TGF-β1 decreases basal OCR in mink lung epithelial cells (Yoon et al., 2005), breast cancer cells (Lee et al., 2016), and activated human effector memory CD4^+^ T cells (Dimeloe et al., 2019), it increases basal and maximal OCR in mouse podocytes (Abe et al., 2013) and human fibroblasts (Selvarajah et al., 2019). Smad proteins are present in the mitochondria of human CD4^+^ T cells and inhibition of Smad2 partially abolishes TGF-β-induced inhibition of respiration (Dimeloe et al., 2019). Similarly, Smad4 deficiency in mouse podocytes elevates the basal and maximal OCR (J. Li et al., 2020a). Conversely, TGF-β1 increases the maximal OCR, mitochondrial mass and membrane potential in mouse CD8^+^ T-cells (Gabriel et al., 2021). The effects of TGF-β1 on hepatocellular carcinoma cell lines is mixed, causing an increase in basal and maximal OCR in SNU449 (Soukupova et al., 2017) and Hep3B (Soukupova et al., 2021) cells, and a decrease in PLC/PRF/5 cells (Soukupova et al., 2017). TGF-β1 broadly promotes respiration in fibroblasts, increasing basal and maximal OCR in mouse fibroblasts (Schwörer et al., 2020; Yin et al., 2019), and both mitochondrial mass and steady state concentrations of TCA intermediates in human lung fibroblasts (Bernard et al., 2018; Sun et al., 2019). TGF-β2 treatment has been reported to suppress maximal OCR in ARPE-19 cells (Shu et al., 2021), however this effect was not observed on ARPE-19 cells maintained in long-term culture prior to treatment (Hansman et al., 2024).

###### 4.2.2.2.3. TGF-β and lipid metabolism.

TGF-β promotes fatty acid uptake and oxidation in several cancer models. Expression of the fatty acid transporter CD36 is induced by TGF-β1 in breast cancer cells (Liu et al., 2020) and by TGF-β2 in cervical cancer cells (Corbet et al., 2020). TGF-β1 additionally promotes expression of CPT1 and increases ATP synthesis in breast cancer cells (Liu et al., 2020), and in human gastric cancer cell lines (Li et al., 2023). While TGF-β1 suppresses PPARγ expression in 3T3-L1 adipocytes (Xing et al., 1997), FAO is increased with TGF-β1 treatment in MCF-7 (Liu et al., 2020), Hep3B (Soukupova et al., 2021), and gastric cancer cells (Li et al., 2023), and with TGF-β2 treatment in cervical cancer cells (Corbet et al., 2020). TGF-β promotes expression of PGC-1α in human lung fibroblasts through Smad2/3 (Sun et al., 2019). The Smad pathway also mediates the TGF-β1-mediated induction of the acid ceramidase ASAH1 in choriocarcinoma JEG3 cells (Chauvin et al., 2015) and the inositol phosphatase, SHIP, in mouse haematopoietic cells (Valderrama-Carvajal et al., 2002).

TGF-β2 promotes lipid droplet formation in cervical cancer cells (Corbet et al., 2020), as well as in mouse bone marrow-derived dendritic cells (Trempolec et al., 2020) and RAW 264.7 macrophages (Bose et al., 2019). Treatment of primary human RPE cells with TGF-β1, 2 and 3 potently induces expression of stearoyl-CoA desaturase (SCD), the rate-limiting enzyme in monounsaturated fatty acid biosynthesis (Samuel et al., 2002). Moreover, metabolic pathway enrichment analysis of TGF-β2-treated hfRPE cells identified altered fatty acid biosynthesis pathways (Ng et al., 2023). Given the role of TGF-β in enhancing FAO in multiple cell types, its effects may oppose those of cytokines like TNFα. Further research is required to more fully understand the role of TGF-β in the regulation of RPE FAO, fatty acid synthesis, and lipid droplet formation.

###### 4.2.2.2.4. TGF-β and amino acid metabolism.

TGF-β participates in the regulation of several amino acid metabolism pathways, including glutaminolysis, serine and glycine biosynthesis, and arginine and tryptophan catabolism. TGF-β1 treatment induces expression of the glutaminolysis enzyme glutaminase 1 (GLS1) in MCF-7 cells (Lee et al., 2016), human and murine lung fibroblasts (Bernard et al., 2018; Choudhury et al., 2020), and human hepatocellular carcinoma cells (Soukupova et al., 2017). In fibroblasts, TGF-β1 promotes glutaminolysis and incorporation of glutamine carbons into TCA cycle intermediates (Schwörer et al., 2020), proline (Schwörer et al., 2020), and glutamate (Bernard et al., 2018; Choudhury et al., 2020). Interestingly, in MCF-7 cells, inhibition of *GLS1* expression prevents TGF-β1-induced glycolytic switch (Lee et al., 2016). Inhibition of Smad3 prevents TGF-β1-induced GLS1 expression in human lung fibroblasts (Bernard et al., 2018). Similarly, in murine lung fibroblasts, GLS1 expression is dependent on Smad2/3 as well as PI3K/mTORC2/platelet-derived growth factor receptor (PDGFR) signalling (Choudhury et al., 2020).

TGF-β1 treatment of A549 non-small cell lung carcinoma (NSCLC) cells induces significant reprogramming of amino acid metabolism, increasing glutamate and aspartate, while decreasing alanine, glycine, asparagine, glutamine, citrulline, proline, and hydroxyproline (Nakasuka et al., 2021). TGF-β1 also induces expression of the tryptophan catabolism enzyme indoleamine 2,3-dioxygenase 1 (IDO1) in murine dendritic cells (Mondanelli et al., 2017). Arginine metabolism is also influenced by TGF-β, promoting arginase activity in rat peritoneal macrophages (Boutard et al., 1995) and arginase 1 (ARG1) expression in mouse dendritic cells (Mondanelli et al., 2017). TGF-β1 promotes arginine uptake by primary rat vascular smooth muscle cells where it is used to synthesise proline (Durante et al., 2001). In human lung fibroblasts, TGF-β1 induces the serine-glycine biosynthesis pathway genes phosphoglycerate dehydrogenase (*PHGDH*), phosphoserine aminotransferase 1 (*PSAT1*), phosphoserine phosphatase (*PSPH*) and serine hydroxymethyltransferase 2 (*SHMT2*) through mTORC1 and Smad3-mediated production of the transcription factor ATF4 (Selvarajah et al., 2019). Lastly, TGF-β1 treatment in human breast epithelial cells reduces expression of the SLC3A2 subunit of the leucine transporter, inhibiting leucine uptake and cell proliferation (Loayza-Puch et al., 2017). Given the emerging importance of amino acids such as serine, glycine, glutamine, and proline in RPE and retina metabolism, overactive TGF-β signalling may have a profound impact on retinal metabolic homeostasis.

#### 4.2.3. IL-6

IL-6 is a pleiotropic cytokine with well characterised roles in both acute and chronic inflammation (Tanaka et al., 2014). IL-6 induces expression of numerous acute phase proteins and promotes immune cell recruitment, proliferation and differentiation (Jones et al., 2018; Tanaka et al., 2014). While mainly expressed by immune cells such as monocytes and macrophages (Akira et al., 1993; Wongchana and Palaga, 2012), IL-6 is also produced by fibroblasts (Akira et al., 1993; Tanaka et al., 2014), myocytes (Akira et al., 1993), endothelial cells (Akira et al., 1993), as well as RPE cells (Elner et al., 1992; Kociok et al., 1998). IL-6 concentration in serum increases with age and has been implicated in numerous pathologies such as Alzheimer’s disease, multiple sclerosis, rheumatoid arthritis, and cancer (Kaur et al., 2020; Maggio et al., 2006). Notably, RPE cells express both the membrane-bound IL-6-recognising receptor molecule (IL-6Rα) and the signal transducer gp130 (Kociok et al., 1998; Leung et al., 2009), allowing for both classical and trans signalling (Wolf et al., 2014).

##### 4.2.3.1. IL-6 signalling in the eye.

As with TNFα and TGF-β, IL-6 is associated with several retinal pathologies. Elevated levels of IL-6 are present in the vitreous of patients with glaucoma (Franks et al., 1992), PVR (Limb et al., 1991), and retinitis pigmentosa (N. Yoshida et al., 2013b), and IL-6 levels in both the aqueous humour and vitreous increase with diabetic retinopathy disease progression (Funatsu et al., 2005; Wu et al., 2017; Zhou et al., 2012). IL-6 is also associated with the characteristic inflammation of AMD, with systemic levels rising with disease progression (Seddon et al., 2005). Increased IL-6 expression is found in the RPE and stroma of patients with CNV (Sato et al., 2018), and its knockdown suppresses CNV and subretinal fibrosis in mouse models (Izumi-Nagai et al., 2007; Sato et al., 2018). IL-6 is secreted by macrophages that accumulate in the sub-retinal space in AMD (Levy et al., 2015), and its production is elevated in macrophage/RPE co-cultures compared with sole cultures, suggesting synergistic action between these cell types (Yamawaki et al., 2016). Expression of IL-6 in the RPE is also stimulated by the drusen components amyloid-β (Liu et al., 2013) and A2E (Zhang et al., 2015).

##### 4.2.3.2. IL-6 and metabolism

###### 4.2.3.2.1. IL-6 and glucose metabolism.

The role of IL-6 in systemic glucose homeostasis and energy metabolism is complex and the subject of ongoing debate (Lehrskov and Christensen, 2019; Wueest and Konrad, 2020). IL-6 administration in mice has been reported to promote insulin insensitivity in the liver (Kim et al., 2004; Klover et al., 2003) and skeletal muscle (Kim et al., 2004). On the other hand, IL-6-deficient mice develop obesity (Wallenius et al., 2002), glucose intolerance (Wallenius et al., 2002), and impaired insulin action (Matthews et al., 2010). Moreover, hydrodynamic gene delivery of IL-6 expression plasmids in obese mice improves insulin sensitivity and glucose tolerance (Ma et al., 2015). IL-6 administration also improves insulin sensitivity, as well as whole-body glucose utilisation and oxygen consumption in healthy human subjects (Carey et al., 2006; Lyngsø et al., 2002; Stouthard et al., 1995; Wueest and Konrad, 2020).

**Fig. 6.**
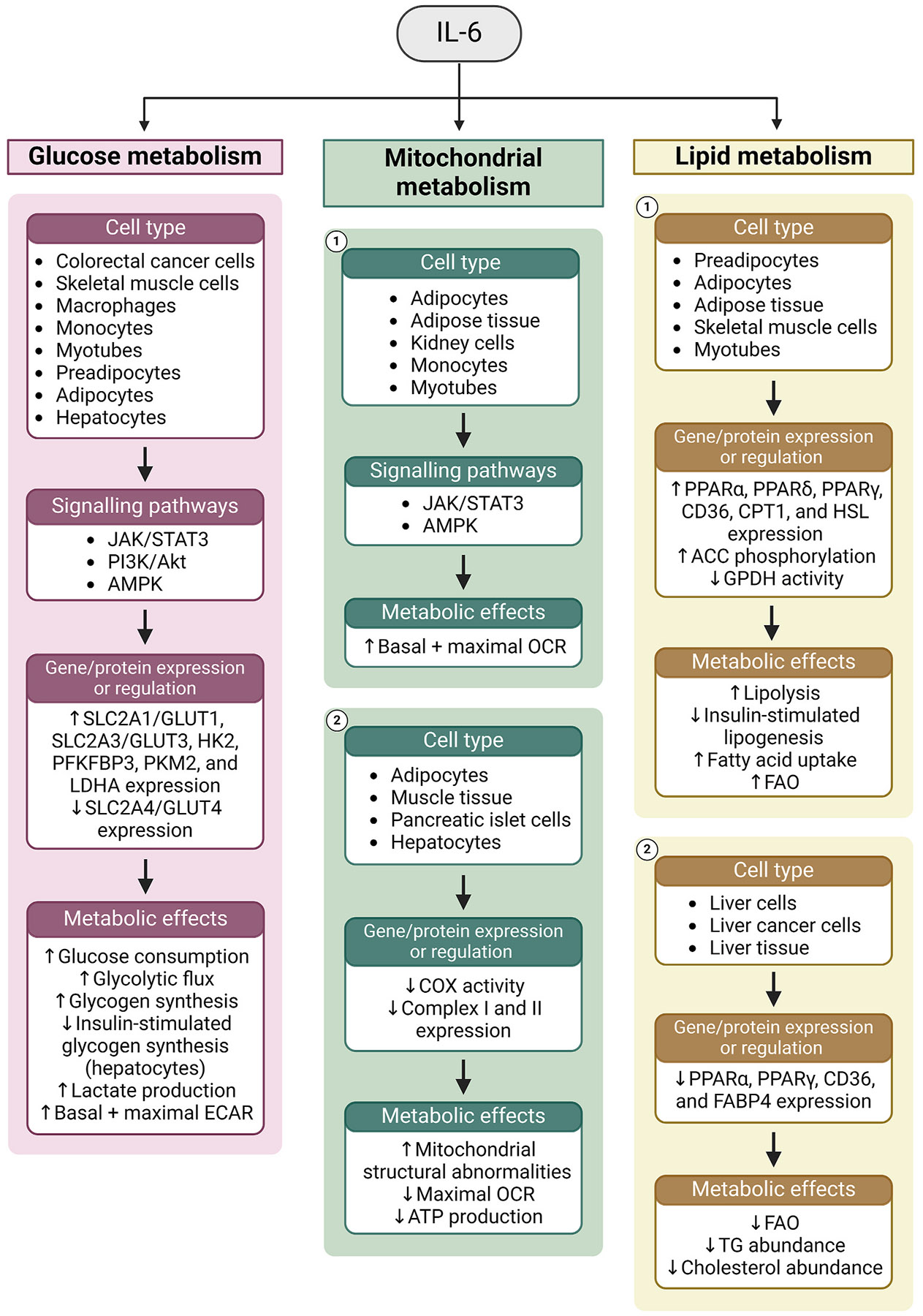
Figure 6. The effects of IL-6 on metabolism. Summary of the effects of IL-6 on glucose (purple), mitochondrial (green), lipid (beige) metabolism in various cell types. Numbers in top left corner of boxes indicate differing effects depending on cell type. ↑ indicates an increase and ↓ indicates a decrease.

IL-6 generally promotes glycolysis and glycolytic gene expression across cell types (Fig. 6, Table 3). Treatment with IL-6 promotes expression of the glycolytic genes *SLC2A1, HK2, PKM2, PFKFB3,* and *LDHA* in human colorectal cancer cells (Han et al., 2016), and similarly induces *Hk2, Pkm2,* and *Pfkfb3* expression in murine macrophages (Kumari et al., 2020). IL-6 also promotes the expression of *SLC2A1* and *SLC2A3* in human monocytes (Xu et al., 2024) and Glut4 translocation to the plasma membrane in rat L6 myotubes (Carey et al., 2006). Conversely, IL-6 suppresses Glut4 expression in mouse adipocytes (Rotter et al., 2003), and both Glut4 and *Gapdh* expression in preadipocytes (Lagathu et al., 2003).

While it may decrease Glut4 expression, IL-6 nevertheless promotes glucose consumption in mouse adipocytes (Ji et al., 2011; Stouthard et al., 1996), as well as mouse macrophages (Kumari et al., 2020), rat myotubes (Carey et al., 2006), and both human skeletal muscle (Al-Khalili et al., 2006; Glund et al., 2007), and colorectal cancer cells (Han et al., 2016). In human monocytes, IL-6 promotes glucose carbon flux to glycolytic and TCA cycle intermediates, while increasing the basal and maximal ECAR (Xu et al., 2024). IL-6 likewise increases lactate production in rat hepatocytes (Kitade et al., 1996), and human colorectal cancer (Han et al., 2016), skeletal muscle cells (Al-Khalili et al., 2006), as well as in ARPE-19 cells (Hansman et al., 2024). While IL-6 promotes glycogen synthesis in primary human skeletal muscle cells (Al-Khalili et al., 2006; Weigert et al., 2005) and *ex vivo* human skeletal muscle tissue (Glund et al., 2007), it inhibits insulin-stimulated glycogen synthesis in primary rat (Kanemaki et al., 1998) and mouse (Senn et al., 2002) hepatocytes. Moreover, IL-6 promotes hepatic glucose release in humans (Stouthard et al., 1995) while suppressing it in rats (Dent et al., 2016), suggesting species-specific effects.

The JAK/STAT3 pathway, comprising Janus kinase (JAK) and signal transducer and activator of transcription 3 (STAT3), is a central pathway activated by IL-6 (Luo and Zheng, 2016). Constitutive STAT3 activation in mouse embryonic fibroblasts leads to a switch to glycolytic metabolism, increasing lactate efflux and the expression of glycolytic genes including *Slc2a1, Pfk,* and enolase (*Eno*) through HIF-1α (Demaria et al., 2010). STAT3 also mediates IL-6-induced increases in HK activity, and succinate, pyruvate, and lactate production in human monocytes (Xu et al., 2024). IL-6 signalling may also influence metabolism through the NF-κB (Bai et al., 2009), MEK/ERK (Ogata et al., 1997), or PI3K/AKT (Wegiel et al., 2008) pathways. Indeed, IL-6 has been reported to influence glucose metabolism through PI3K/AKT in rat myotubes (Carey et al., 2006), and both mouse macrophages (Kumari et al., 2020) and skeletal muscle cells (Al-Khalili et al., 2006).

AMPK is a central regulator of cellular energy homeostasis. When activated by rising AMP/ATP ratio, it phosphorylates multiple downstream targets to enhance catabolic pathways and inhibit anabolic processes, thereby maintaining energy balance within the cell (Hardie et al., 2012; Kahn et al., 2005). AMPK suppresses mTOR activity, decreasing protein synthesis and cell growth, and inhibits glycogen synthase to lower glycogen synthesis. Additionally, it activates PFK2 to boost glycolysis and facilitates GLUT4 translocation to the plasma membrane to increase glucose uptake in muscle cells (Hardie et al., 2012; Jeon, 2016). IL-6 increases AMPK phosphorylation in rat (Kelly et al., 2004) and mouse (Biensø et al., 2014) skeletal muscle, L6 rat myotubes (Carey et al., 2006), and mouse adipocytes (Kelly et al., 2004), presenting another avenue through which IL-6 may influence glucose metabolism.

###### 4.2.3.2.2. IL-6 and mitochondrial metabolism.

IL-6 may promote or inhibit mitochondrial metabolism depending on cell type. IL-6 promotes PGC-1α expression in both mouse adipocytes (Ji et al., 2011) and rat podocytes (Kim and Park, 2013), while hydrodynamic gene delivery of IL-6 in mice increases the expression of PGC-1α in brown adipose tissue (BAT) (Ma et al., 2015). These effects may be mediated by activation of AMPK which activates PGC-1α to promote mitochondrial biogenesis and oxidative metabolism (Hardie et al., 2012). IL-6 treatment increases the maximal OCR in human monocytes (Xu et al., 2024), and both basal and maximal OCR in mouse myotubes through activation of JAK/STAT3 (Abid and Ryan, 2020). On the other hand, IL-6 treatment causes mitochondrial structural abnormalities in murine adipocytes (Ji et al., 2011) and overexpression of IL-6 decreases cytochrome *c* oxidase (COX) activity and complex I and II protein abundance in mouse muscle tissue (Vanderveen et al., 2019). Moreover, activation of IL-6 trans signalling decreases the mitochondrial reserve capacity and increases the relative reliance on glycolysis for ATP production in human retinal endothelial cells (Hoffman et al., 2023). IL-6 treatment also suppresses total ATP production in primary rat hepatocytes (Kitade et al., 1996) and pancreatic islet cells (Sandler et al., 1990), suggesting perturbed OXPHOS in these cells. Taken together, these data suggest that in most cells IL-6 hinders ATP production through OXPHOS, leading to AMPK activation, PGC-1α induction, and increased glycolysis.

###### 4.2.3.2.3. IL-6 and lipid metabolism.

IL-6 broadly stimulates whole-body lipolysis and FAO. IL-6 administration in humans induces a rapid increase in systemic FAO (Lyngsø et al., 2002; Van Hall et al., 2003; Wolsk et al., 2010) and the release of glycerol (Van Hall et al., 2003), fatty acids (Petersen et al., 2005; Van Hall et al., 2003), and the ketone bodies β-HB and acetoacetate (Stouthard et al., 1995), into the plasma. While IL-6 administration has been reported to increase serum TG, cholesterol, and FFA concentrations in rats (Nonogaki et al., 1995), IL-6^−/−^ mice exhibit higher serum TG and total cholesterol than wild-type mice when fed a high fat diet (HFD) (Chen et al., 2017). IL-6 exerts a lipolytic effect on adipocytes in a similar manner to TNFα. IL-6 treatment stimulates glycerol release from cultured murine (Ji et al., 2011), and human adipocytes (Päth et al., 2001), human adipose tissue explants (Trujillo et al., 2004), and human adipose tissue *in vivo* (Lyngsø et al., 2002). IL-6 suppresses fatty acid synthase (*Fasn*) expression and insulin-stimulated lipogenesis from glucose in murine preadipocytes (Lagathu et al., 2003) and decreases glycerol 3-phosphate dehydrogenase activity in human adipocytes (Päth et al., 2001). Moreover, IL-6 increases FAO in both rat myotubes (Carey et al., 2006) and mouse adipocytes (Petersen et al., 2005), and hydrodynamic gene delivery of IL-6 increases *Cd36*, *Cpt1*, and *Hsl* expression in mouse adipose tissue *in vivo* (Ma et al., 2015).

IL-6 promotes PPARα, δ, and γ expression, fatty acid uptake, and β-oxidation in human skeletal muscle cells (Al-Khalili et al., 2006; Bruce and Dyck, 2004). Conversely, IL-6 inhibits FAO in primary rat hepatocytes (Nachiappan et al., 1994) and suppresses PPARα expression in hepatocarcinoma cells (Chew et al., 2007). Similarly, IL-6 gene delivery in mice decreases expression of PPARγ, along with the fatty acid transporters Cd36 and Fabp4 in the liver (Ma et al., 2015). IL-6 overexpression also decreases hepatic TG and cholesterol (Ma et al., 2015), which are also depleted in the medium of HepG2 cells treated with IL-6 (Ettinger et al., 1994). AMPK inhibits ACC1 and ACC2 to decrease fatty acid synthesis and increase FAO, and it inhibits HMGCR to reduce cholesterol synthesis (Jeon, 2016). IL-6 increases ACC phosphorylation in mouse skeletal muscle (Biensø et al., 2014), L6 rat myotubes (Carey et al., 2006), and mouse adipocytes (Kelly et al., 2004). Thus, unlike TNFα, IL-6 may promote FAO in the RPE. However, these effects, as well as those on other aspects of lipid metabolism, require experimental validation.

#### 4.2.4. IL-1β

IL-1β is a major inflammatory mediator released in response to infection or injury, modulating the differentiation and function of various lymphocytes (Gabay et al., 2010; Weber et al., 2010). Although primarily produced by stimulated monocytes and macrophages (Gabay et al., 2010), IL-1β is secreted by diverse cell types including microglia (Garlanda et al., 2013), smooth muscle cells (Gabay et al., 2010), fibroblasts (Holzberg et al., 2003), and RPE cells (Holtkamp et al., 2001; Planck et al., 1993). IL-1β is processed into its mature, active form in response to PAMPs and DAMPs by the protease caspase-1 (Afonina et al., 2015), which is activated by the NLR family pyrin domain containing 3 (NLRP3) inflammasome (Garlanda et al., 2013). Both the membrane-bound IL-1β receptor 1 (IL-1R1) and its co-receptor, IL-1RAcP, are expressed ubiquitously, including in human RPE cells (Kociok et al., 1998; Leung et al., 2009; Shi et al., 2008; Towne et al., 2004; Weber et al., 2010).

##### 4.2.4.1. IL-1β signalling in the eye.

IL-1β is a key mediator of the inflammatory conditions associated with an array of ocular diseases (Wooff et al., 2019). Vitreous levels of IL-1β are elevated in retinitis pigmentosa (N. Yoshida et al., 2013a), and increased IL-1β levels are found in the serum (Demircan et al., 2006), vitreous (Demircan et al., 2006; Zhou et al., 2012), and aqueous humour (Wu et al., 2017) of patients with diabetic retinopathy. Increased retinal IL-1β is also found in animal models of retinal detachment (Kataoka et al., 2015; Nakazawa et al., 2006), and light-induced retinal degeneration (Ni et al., 2008; M. Zhang et al., 2012a). Elevated IL-β levels are present in the vitreous (Zhao et al., 2015) and serum (Nassar et al., 2015) of patients with CNV, while IL-1β inhibition prevents laser-induced CNV in animal models (Lavalette et al., 2011). IL-1β mRNA is also elevated in the RPE of patients with dry AMD (Tarallo et al., 2012). IL-1β expression in the RPE is stimulated by oxidative stress (Wooff et al., 2019), lipofuscin (Wooff et al., 2019), lysosomal destabilisation (Tseng et al., 2013), and the drusen components amyloid-β (Liu et al., 2013) and A2E (Zhang et al., 2015).

##### 4.2.4.2. IL-1β signalling and metabolism

###### 4.2.4.2.1. IL-1β and glucose metabolism.

As with IL-6, the effects of IL-1β on systemic glucose metabolism are controversial, with numerous animal studies reporting conflicting results. On the one hand, IL-1β administration induces rapid hypoglycaemia in mice (Del Rey et al., 2006), and injection of mice with IL-1β-secreting tumour cells decreases serum glucose levels by increasing glucose uptake by the peripheral tissues and decreasing hepatic gluconeogenesis (Metzger et al., 2004). Moreover, mature age IL-1RI^−/−^ mice exhibit decreased glucose tolerance and insulin sensitivity (García et al., 2006). On the other hand, IL-1β administration has been reported to promote insulin resistance in mice (Wen et al., 2011), and IL-1β treatment in rats gradually increases blood glucose levels over 48 h (Todaro et al., 2003). Additionally, IL-1β^−/−^ and IL-1RI^−/−^ mice exhibit improved insulin sensitivity (McGillicuddy et al., 2011; Stienstra et al., 2010) and glucose tolerance (McGillicuddy et al., 2011). Treatment of type 2 diabetic GK rats with the IL-1R antagonist (IL-1Ra) reduces hyperglycaemia and improves insulin sensitivity (Ehses et al., 2009). Similarly, IL-1Ra treatment decreases fasting blood glucose concentrations in human type 2 diabetes patients (Larsen et al., 2007). Moreover, IL-1β has been shown to promote insulin resistance *in vitro,* both in mouse adipocytes (Handa et al., 2013; Jager et al., 2007) and hepatocytes (Wen et al., 2011).

IL-1β induces the expression of a host of glycolytic genes, including *Slc2a1, Slc2a3, Hk2, Pfkl, Pkm2,* and *Ldha* in mouse epithelial cells (Qian et al., 2018). IL-1β treatment promotes GLUT1 expression in mouse adipocytes (Jager et al., 2007) and human induced Treg (iTreg) cells (Feldhoff et al., 2017), and both the Glut1 and Glut3 glucose transporters in rat ovary cells (Kol et al., 1997). Injection of mice with IL-1β-secreting tumour cells increases hepatic *Slc2a3* expression (Metzger et al., 2004). Moreover, IL-1β induces *HK2* in human glioma cells (Gupta et al., 2017) and *Ldha* in rat Sertoli cells (Riera et al., 2007). Conversely, IL-1β decreases expression of both *GLUT4* and glycogen synthase kinase 3 beta (*GSK-3β*) in primary human adipocytes (Gao et al., 2014).

**Fig. 7.**
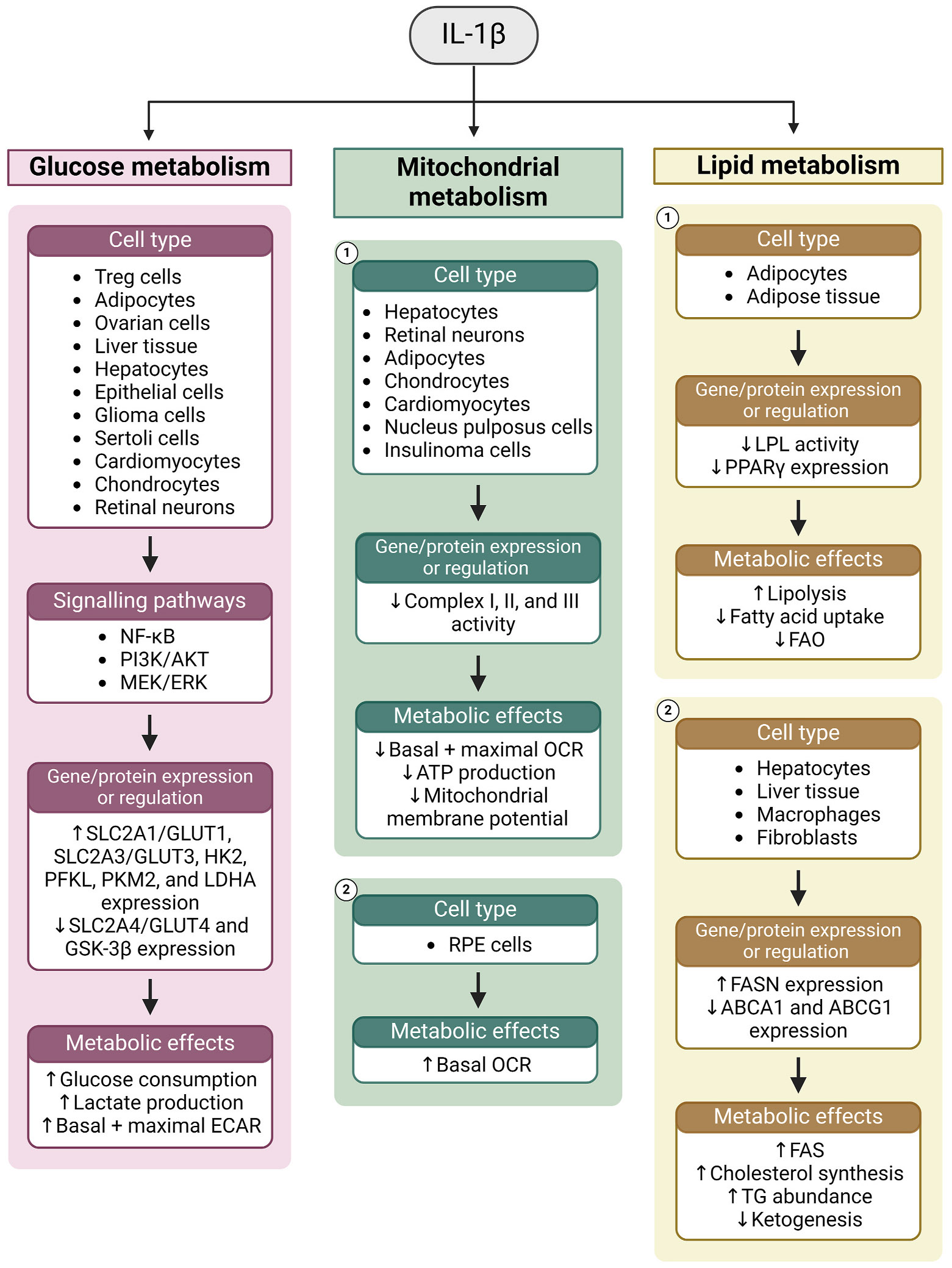
Figure 7. The effects of IL-1β on metabolism. Summary of the effects of IL-1β on glucose (purple), mitochondrial (green), lipid (beige) metabolism in various cell types. Numbers in top left corner of boxes indicate differing effects depending on cell type. ↑ indicates an increase and ↓ indicates a decrease.

IL-1β promotes glucose consumption and lactate production across most cell types (Fig. 7, Table 4). Treatment with IL-1β increases glucose consumption in mouse epithelial cells (Qian et al., 2018) and adipocytes (Jager et al., 2007), rat ovarian cells (Ben-Shlomo et al., 1997; Kol et al., 1997), cardiomyocytes (Tatsumi et al., 2000), and Sertoli cells (Riera et al., 2007), as well as human chondrocytes (Lee et al., 2002). IL-1β also increases glucose consumption in the r28 and RGC-5 retinal neuron cell lines (Abcouwer et al., 2008). Conversely, IL-1β decreases glucose utilisation in rat pancreatic islet cells (Ma et al., 1997), and long-term IL-1β treatment decreases insulin-stimulated glucose uptake and utilisation in mouse 3T3-F442A and 3T3-L1 adipocytes (Lagathu et al., 2006). IL-1β increases lactate production in rat hepatocytes (Kitade et al., 1996), ovarian cells (Ben-Shlomo et al., 1997), Sertoli cells (Riera et al., 2007), cardiomyocytes (Tatsumi et al., 2000), and retinal neurons (Abcouwer et al., 2008), mouse tracheal epithelial cells (Qian et al., 2018), as well as human chondrocytes (Lee et al., 2002) and ARPE-19 cells (Hansman et al., 2024). Additionally, IL-1β increases the basal and maximal ECAR in primary mouse epithelial cells (Qian et al., 2018) and adipocytes (Zhou et al., 2020).

IL-1β activates multiple signalling networks that may influence glucose metabolism. For example, the active IL-1R1 complex shares downstream targets with TNFα such as NF-κB (Cao et al., 1996; Sizemore et al., 1999) and MEK/ERK (Riera et al., 2007). The effects of IL-1β on lactate production and glycolytic gene expression in mouse tracheal epithelial cells is mediated by IKKε (Qian et al., 2018), a protein kinase involved in NF-κB activation (Taniguchi and Karin, 2018). IL-1 signalling also activates the PI3K/AKT pathway which regulates glucose metabolism (Riera et al., 2007; Sizemore et al., 1999). In rat Sertoli cells, IL-1β-induced *Ldha* expression is dependent on MEK/ERK, glucose uptake on PI3K/AKT, while lactate production is dependent on both pathways (Riera et al., 2007). Moreover, HIF-1α induction by IL-1β in human Treg cells is dependent on mTORC1 (Feldhoff et al., 2017).

###### 4.2.4.2.2. IL-1β and mitochondrial metabolism.

IL-1β has broad inhibitory effects on mitochondrial OXPHOS and ATP production. IL-1β treatment decreases the basal OCR in rat hepatocytes (Berthiaume et al., 2003) and R28 retinal neurons (Abcouwer et al., 2008), the maximal OCR in human Treg cells (Feldhoff et al., 2017), while decreasing both the basal and maximal OCR in mouse adipocytes (Zhou et al., 2020) and human chondrocytes (Eitner et al., 2021). IL-1β suppresses total ATP production in rat hepatocytes (Kitade et al., 1996), cardiomyocytes (Tatsumi et al., 2000), and R28 retinal neurons (Abcouwer et al., 2008), as well as in human chondrocytes (Eitner et al., 2021; López-Armada et al., 2006) and nucleus pulposus cells (Shen et al., 2017). Moreover, IL-1β decreases the mitochondrial membrane potential in rat insulinoma cells (Veluthakal et al., 2005) and human nucleus pulposus cells (Shen et al., 2017). IL-1β treatment also decreases mitochondrial complex I activity in human chondrocytes (López-Armada et al., 2006), complex I and II activity in rat cardiomyocytes (Tatsumi et al., 2000), and complex I and III activity in mouse adipocytes (Zhou et al., 2020). Interestingly, IL-1β increases the basal OCR in ARPE-19 cells (Hansman et al., 2024), suggesting that the effects of this cytokine on RPE mitochondrial metabolism may contrast with most other cell types.

###### 4.2.4.2.3. IL-1β and lipid metabolism.

IL-1β administration in mice results in increased serum TG (Feingold et al., 1994; Memon et al., 1993) and cholesterol (Memon et al., 1993) levels. In a similar manner to TNFα, IL-1β inhibits fatty acid uptake and oxidation in adipocytes, increasing lipolysis despite decreasing LPL activity. IL-1β decreases LPL activity in mouse adipose tissue *in vivo* (Feingold et al., 1994), in cultured 3T3-L1 adipocytes (Beutler and Cerami, 1985; Price et al., 1986a), and in primary mouse preadipocytes (Grégoire et al., 1992). However, IL-1β increases lipolysis in human (Gao et al., 2014), and mouse (Lagathu et al., 2006; Price et al., 1986a) adipocytes. IL-1β also inhibits fatty acid uptake and oxidation in mouse adipocytes (Handa et al., 2013; Zhou et al., 2020), while decreasing PPARγ expression in both human and mouse adipocytes (Gao et al., 2014; Lagathu et al., 2006).

IL-1β administration in mice promotes hepatic fatty acid (Feingold et al., 1989b; Grunfeld et al., 1990) and cholesterol (Feingold et al., 1989b; Memon et al., 1993) synthesis *in vivo*. Moreover, IL-1β treatment increases FAS protein expression (Negrin et al., 2014) and TG abundance (Miura et al., 2010; Negrin et al., 2014) in cultured mouse hepatocytes. Treatment also decreases ketone body production in primary rat hepatocytes (Kitade et al., 1996), suggesting that IL-1β may inhibit β-oxidation in the liver. Lastly, IL-1β decreases ABCA1 and ABCG1 expression in J774 macrophages (Khovidhunkit et al., 2003), and promotes cholesterol ester biosynthesis in human skin fibroblasts (Kronqvist et al., 1999). Given that it decreases fatty acid uptake and oxidation across multiple cell types, IL-1β may also exert a similar influence on RPE lipid metabolism.

## 5. Oxidative stress and RPE metabolism

**Fig. 8.**
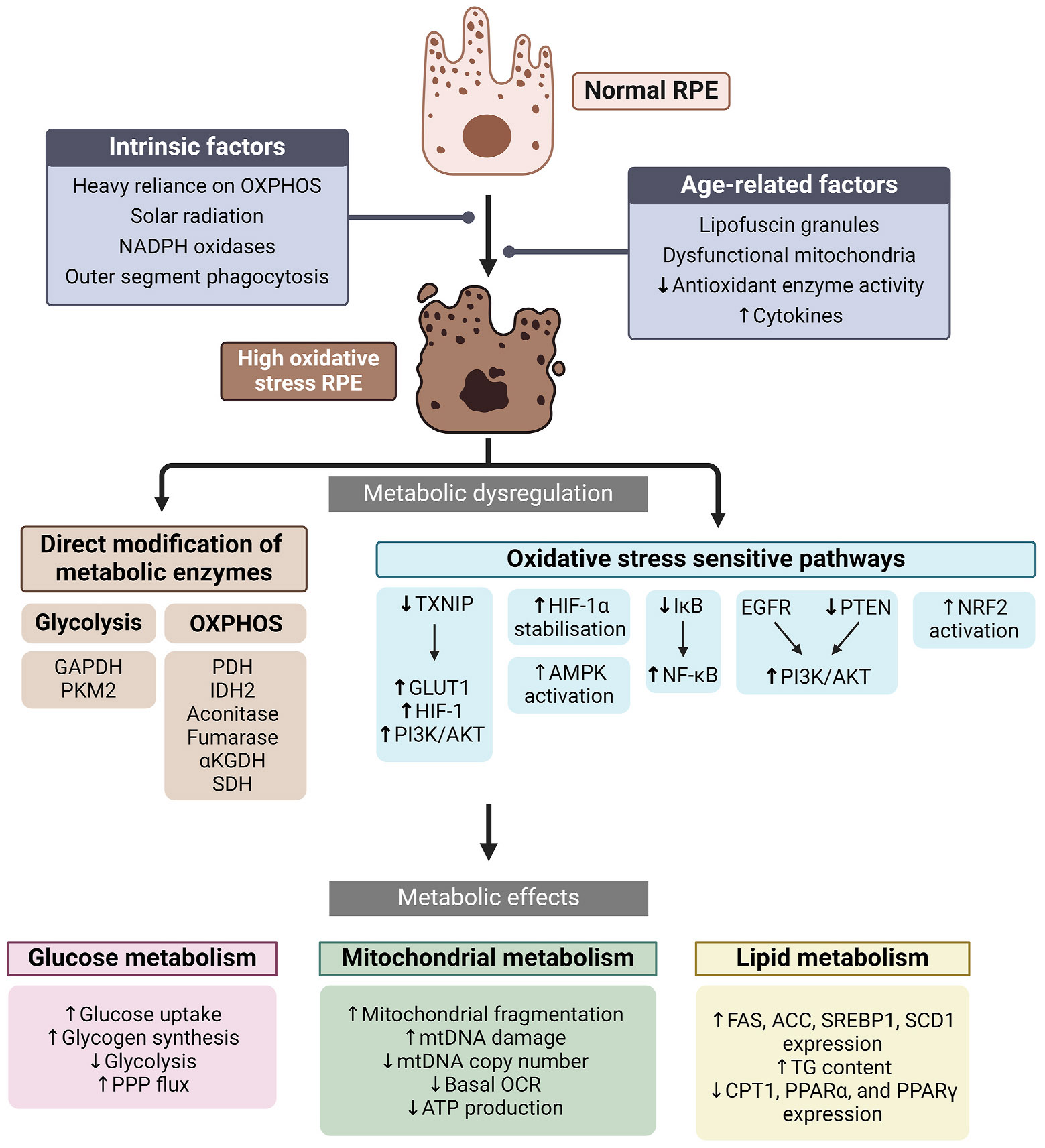
Figure 8. The sources of oxidative stress in the RPE and their effects on metabolism. Multiple intrinsic and age-related factors contribute to severe oxidative stress in the RPE. Reactive oxygen species may influence metabolism through direct modification of metabolic enzymes or through oxidative stress sensitive pathways. This results in dysregulated glucose (pink), mitochondrial (green), and lipid (beige) metabolism. ↑ indicates an increase and ↓ indicates a decrease.

Due to its anatomical position and physiological functions, a significantly high degree of oxidative stress is imposed upon the RPE. This stress is intensified by aging and disease-related factors, further exacerbating the burden met by these cells. As discussed below, the influence of oxidative stress on cellular metabolism is well established, either through direct protein modification, or by stimulating ROS-sensitive pathways (Fig. 8). Besides its broader detrimental effects, more specifically, oxidative stress may disrupt the metabolic balance in the retina with advancing age.

### 5.1. Sources of oxidative stress in the RPE

Recent research highlights the heavy reliance of the RPE on mitochondrial OXPHOS (Kanow et al., 2017; Zhang et al., 2021), which may be a key contributor to high levels of oxidative stress in these cells. Given that mitochondria are believed to be the source of approximately 90% of cellular ROS (Balaban et al., 2005), this may impose a particularly severe burden on these cells. Furthermore, the outer retina is susceptible to superoxide ($O_{2}^{-}$) generation due to incoming solar radiation (Young, 1988), introducing another source of oxidative stress.

NADPH oxidases may present another major ROS generator in the RPE. These enzymes transport electrons across lipid membranes, generating $O_{2}^{-}$ (Panday et al., 2015). Although conventionally associated with the anti-microbial respiratory burst of phagocytic immune cells such as neutrophils, members of the NADPH oxidase family are now known to be expressed in virtually all tissues (Bedard and Krause, 2007). The respiratory burst during photoreceptor OS phagocytosis in the RPE resembles that of other phagocytes and may be mediated by NADPH oxidases (Bedard and Krause, 2007; Miceli et al., 1994). Thus, the daily phagocytosis of photoreceptor OSs may also be a potent source of free radicals. Indeed, phagocytosis prompts a substantial release of $O_{2}^{-}$ from cultured RPE cells (Dorey et al., 1989; Tate et al., 1995). Moreover, photoreceptor membranes contain particularly high levels of polyunsaturated fatty acids (PUFAs) (Fliesler and Anderson, 1983), which, when phagocytosed by the RPE, offer another source of ROS (Jarrett and Boulton, 2012).

#### 5.1.1. Oxidative stress in the aging RPE

The ‘free radical theory’ of aging, proposed in the 1950s, attributes the deleterious effects of aging to the accumulated damage caused by oxygen radicals (Harman, 1956). Indeed, aged primary RPE cells exhibit increased ROS production (Rohrer et al., 2016). The unique characteristics of the RPE may present particularly potent and pernicious sources of oxidative stress with aging compared with other tissues.

Lipofuscin granules accumulate gradually in the RPE with age, increasing fourfold between the 1st and 6th decade of life (Feeney-Burns et al., 1984). When exposed to white light, granules isolated from elderly donors were estimated to generate 3.5 × 10^8^ molecules of $O_{2}^{-}$ per RPE cell per minute (Winkler et al., 1999), which increases in proportion to light intensity (Boulton et al., 1993). Concomitantly, autophagy and mitophagy systems decrease in activity with age (Barbosa et al., 2019), leading to even further ROS production from damaged or dysfunctional mitochondria (Lee et al., 2012; Murphy, 2009). This can be particularly consequential for the RPE given its high reliance on mitochondrial OXPHOS for energy. Additionally, the aging RPE may suffer from an inability to clear free radicals, as activity levels of the antioxidant enzyme catalase decrease with age and in AMD (Castorina et al., 1992; Liles et al., 1991). Moreover, lipid droplets accumulate in the RPE/choroid of aged mice and suppression of lipid droplet accumulation decreases ROS production in ARPE-19 cells (Yako et al., 2022). In addition to directly influencing metabolism as previously discussed, cytokines such as TNFα (Kastl et al., 2014) and IL-6 (Abid and Ryan, 2020; Ji et al., 2011) may also contribute to oxidative stress by generating ROS.

Oxidative stress is a well-established factor in AMD pathophysiology (Beatty et al., 2000; Campagne et al., 2014; Datta et al., 2017; Jarrett and Boulton, 2012). Genetic variants of mitochondrial enzymes that generate ROS correlate with increased disease risk (Datta et al., 2017). Moreover, a polymorphism in the antioxidant enzyme superoxide dismutase 2 (SOD2) gene has been linked to CNV (Kimura et al., 2000). *Sod1* and *Sod2*-deficient mice, as well as mice lacking the antioxidant transcription factor, nuclear factor erythroid 2-related factor 2 (NRF2), mimic many pathological features of AMD including drusen-like deposits, declining ERG responses, CNV, and photoreceptor degeneration (Imamura et al., 2006; Mao et al., 2014; Z. Zhao et al., 2011b). RPE from AMD donors have also been reported to exhibit decreased glutathione (Ferrington et al., 2017), increased ROS production, and decreased cell viability under oxidative stress (Golestaneh et al., 2017; He and Tombran-Tink, 2010). However, this remains controversial as other investigations have reported that RPE from AMD donors demonstrate increased resistance to oxidative damage compared to non-AMD donors (Ferrington et al., 2017).

### 5.2. Oxidative stress and metabolism

#### 5.2.1. Oxidative stress and glucose metabolism

Although its effect vary somewhat depending on cell type, oxidative stress generally promotes glucose uptake while inhibiting glycolysis (Fig. 8, Table 5). Glucose consumption is decreased upon H_2_O_2_ treatment in mouse lymphocytes (Hyslop et al., 1988), as well as with H_2_O_2_ induction with glucose oxidase (GO) treatment in rat endothelial cells (Fernandes et al., 2011). On the other hand, H_2_O_2_ treatment promotes glucose uptake in rat skeletal muscle explants (Archuleta et al., 2009; Dokken et al., 2008; Higaki et al., 2008; Jensen et al., 2008; Kim et al., 2006; Toyoda et al., 2004), cultured rat skeletal muscle cells (Pinheiro et al., 2010), Lewis lung carcinoma cells (Jung et al., 2013), rat (Hayes and Lockwood, 1987) and mouse adipocytes (Prasad and Ismail-Beigi, 1999), mouse myoblasts (Prasad and Ismail-Beigi, 1999), fibroblasts, and hepatocytes (Prasad and Ismail-Beigi, 1999). Similarly, GO treatment increases glucose uptake by rat myotubes (Kozlovsky et al., 1997), and treatment of Lewis lung carcinoma (Jung et al., 2013) and human leukemia cells (Prata et al., 2008) with antioxidants decreases glucose uptake. While GO treatment of rat endothelial cells decreases *Slc2a1* expression and promotes Glut1 proteasomal degradation (Fernandes et al., 2011), GO reportedly increases Glut1 expression in rat myotubes (Kozlovsky et al., 1997) and mouse adipocytes (Rudich et al., 1997). Moreover, both pharmacological and genetic activation of Nrf2 in mice *in vivo* results in increased hepatic *Slc2a1* expression (Yates et al., 2009).

H_2_O_2_ treatment of murine lymphocytes (Hyslop et al., 1988) and rat cardiomyocytes (Janero et al., 1994) has been reported to decrease lactate production. Conversely, lactate production is increased upon H_2_O_2_ treatment in rat skeletal muscle cells (Pinheiro et al., 2010), and decreased with the antioxidant resveratrol in Lewis lung carcinoma cells (Jung et al., 2013). The widely reported stimulation of glucose uptake by ROS, coupled with the lack of increased lactate production, suggests that glycolysis is inhibited. Indeed, H_2_O_2_ treatment decreases GAPDH activity in mouse lymphocytes (Hyslop et al., 1988), mouse lens epithelial cells (Spector et al., 2002), and rat cardiomyocytes (Janero et al., 1994). Decreased GADPH activity may be attributable to decreased levels of its essential cofactor, NAD^+^, upon H_2_O_2_ treatment (Hyslop et al., 1988; Spector et al., 2002), which is consumed by poly (ADP-ribose) polymerase (PARP) enzymes in response to DNA damage (Bai, 2015). Moreover, GAPDH (Hwang et al., 2009), as well as PKM2 (Anastasiou et al., 2011), are directly oxidised by H_2_O_2_, inhibiting their function.

Cells may use redox-based inhibition of glycolytic enzymes to drive flux through the PPP in oxidative conditions (Stincone et al., 2015). Oxidative stress depletes intracellular NADPH, causing the cell to respond by increasing flux through the oxidative PPP – a major source of NADPH in mammalian cells (Ge et al., 2020). PPP flux may also be augmented by glucose 6-phosphate dehydrogenase (G6PD), the first enzyme of the PPP, which is sensitive to the NADP^+^/NADPH ratio and therefore the cell’s redox balance (Holten et al., 1976; Kotaka et al., 2005). Indeed, H_2_O_2_ exposure increases Hk and G6PD activity in rat skeletal muscle cells (Pinheiro et al., 2010), and increases glucose carbon flux through the PPP in mouse lymphocytes (Hyslop et al., 1988). Excess glucose that is not metabolised by glycolysis or the PPP may also be incorporated into glycogen. Treatment of rat skeletal muscle explants with H_2_O_2_ increases glycogen synthase activity (Dokken et al., 2008; Kim et al., 2006) and glycogen synthesis (Dokken et al., 2008).

Oxidative stress may also influence glucose metabolism through the activation of intracellular signalling networks (Fig. 8). While mitochondria-derived H_2_O_2_ is required for HIF-1α stabilisation in hypoxia (Brunelle et al., 2005; Sanjuán-Pla et al., 2005), exogenous H_2_O_2_ treatment is sufficient to stabilise HIF-1α protein even under normoxia, promoting HIF-1 transcriptional activity (Chandel et al., 2000). ROS-mediated HIF-1 activation has been identified as a driver of aerobic glycolysis some cancer models (Shi et al., 2009). Additionally, ROS may activate or inhibit NF-κB signalling, depending on context (Morgan and Liu, 2011). H_2_O_2_ can also stimulate the PI3K/AKT pathway through multiple mechanisms (Leslie et al., 2003; Wang et al., 2000), including in cultured RPE cells (Yang et al., 2006). H_2_O_2_-stimulated glucose uptake is dependent on PI3K (Higaki et al., 2008; Kim et al., 2006) and p38 MAPK (Archuleta et al., 2009; Kim et al., 2006) signalling in rat skeletal muscle explants, and phospholipase C (PLC) in rat liver epithelial cells (Prasad and Ismail-Beigi, 1999). Moreover, H_2_O_2_ treatment promotes AMPK phosphorylation in human embryonic kidney cells (Zmijewski et al., 2010) and mouse fibroblasts (Choi et al., 2001), as well as AMPK activity in rat skeletal muscle explants (Jensen et al., 2008; Toyoda et al., 2004). Indeed, mitochondria-derived H_2_O_2_ is required for hypoxic activation of AMPK, independent of the AMP/ATP ratio (Emerling et al., 2009).

Oxidative stress may also influence insulin resistance, however its effects are duration and dose-dependent (Iwakami et al., 2011). H_2_O_2_ causes short term enhancement of insulin signalling by inhibiting tyrosine phosphatase activity (Rains and Jain, 2011). However, chronic oxidative stress may promote insulin resistance through several mechanisms such as (inhibitory) serine/threonine insulin receptor substrate (IRS) phosphorylation, cellular redistribution of insulin signalling components, decreasing GLUT4 expression, altering mitochondrial activity, and promoting JNK phosphorylation (Rains and Jain, 2011; Rindler et al., 2013).

H_2_O_2_ also downregulates expression of thioredoxin-interacting protein (TXNIP) (Schulze et al., 2002), which restricts glucose uptake by decreasing *SLC2A1* mRNA levels and promoting the internalisation of GLUT1 transporters (Wu et al., 2013). TXNIP also suppresses glycolysis by inhibiting HIF-1 transcriptional activity (Farrell et al., 2010) and by inhibiting the PI3K/AKT pathway (Hui et al., 2008). Indeed, HEK 293T cells overexpressing TXNIP exhibit decreased glycolytic and PPP metabolites (Kim et al., 2024). TXNIP expression is typically upregulated by high glucose conditions (Fang et al., 2011; Minn et al., 2005), including in the RPE, where it is associated with mitochondrial membrane depolarisation, fragmentation and mitophagic flux (Devi et al., 2019). Oxidative stress rapidly downregulates TXNIP protein in RPE cells (Ji Cho et al., 2019), while its knockdown causes autophagic flux, AMPK activation, and HIF-1α upregulation (Ji Cho et al., 2019).

Thus, oxidative stress may inhibit glycolysis through direct modification of glycolytic enzymes or enhance it through downregulation of TXNIP or activation of pro-glycolytic pathways, such as HIF-1, NF-κB and PI3K/AKT. Given the context-dependent nature of the metabolic effects of oxidative stress, the exact manner in which oxidative stress influences RPE glucose metabolism is still largely an open question requiring more extensive investigation.

#### 5.2.2. Oxidative stress and mitochondrial metabolism

Oxidative stress generally has an adverse effect on the bioenergetic status of cells, with H_2_O_2_ treatment decreasing ATP production in mouse lens epithelial cells (Spector et al., 2002), mouse lymphocytes (Hyslop et al., 1988), rat cardiomyocytes (Janero et al., 1994), and human erythrocytes (Tavazzi et al., 2000). ROS cause numerous mitochondrial defects, inducing mtDNA damage (Alexeyev, 2009), decreasing mtDNA copy number (Bonnard et al., 2008), and promoting mitochondrial fragmentation (Jahani-Asl et al., 2007). H_2_O_2_ treatment decreases the basal OCR in mouse lymphocytes (Hyslop et al., 1988), while the antioxidant resveratrol increases the basal OCR of LLC cells (Jung et al., 2013). Conditional knockout of the antioxidant enzyme Sod2 in mouse RPE cells leads to changes in mitochondrial structure and function, as well as a compensatory increase in glycolytic gene expression (Brown et al., 2019).

As with glycolytic enzymes, ROS may directly oxidise TCA cycle enzymes, inhibiting their function. H_2_O_2_ and $O_{2}^{-}$ oxidise and decrease the activity of numerous TCA cycle enzymes including PDH (Cabiscol et al., 2000), isocitrate dehydrogenase (Lee et al., 2001), aconitase (Cabiscol et al., 2000; Gardner, 2002; Lushchak et al., 2014; NultonαPersson and Szweda, 2001), fumarase (Jang and Imlay, 2007), α-ketoglutarate dehydrogenase (Cabiscol et al., 2000; Nulton-Persson and Szweda, 2001), and succinate dehydrogenase (Nulton-Persson and Szweda, 2001). Moreover, Inhibition of NADPH oxidase increases skeletal muscle complex I and III activity in mice fed a HFD (Yokota et al., 2009). H_2_O_2_ treatment decreases citrate synthase activity in mouse myotubes (Bonnard et al., 2008), and both PDH activity and glucose oxidation to CO_2_ in rat cardiomyocytes (Janero et al., 1994).

NRF2 is a transcription factor that is activated in response to ROS exposure and plays a central role in orchestrating a transcriptional response to oxidative stress and inflammation. NRF2 may have a protective effect to offset the direct effects of ROS on mitochondrial metabolism. Nrf2 null mice exhibit decreased liver NADH (Y. K. J. Zhang et al., 2012b), while primary adipocytes isolated from Nrf2^−/−^ mice exhibit increased basal and maximal OCR (Sun et al., 2020). Additionally, Nrf2^−/−^ mouse embryonic fibroblasts (MEFs) exhibit decreased mitochondrial membrane potential, basal OCR, and mitochondrial NADH pool (Holmström et al., 2013). Moreover, heart slices (Ludtmann et al., 2014) and primary cortical neurons (Holmström et al., 2013) isolated from Nrf2^−/−^ mice have a high FAD/FADH_2_ ratio compared to wildtype, suggesting an impaired TCA cycle.

#### 5.2.3. Oxidative stress and lipid metabolism

H_2_O_2_ treatment increases FAS, ACC, and SREBP expression and increases the TG content of mouse adipocytes (Sun et al., 2020) and HepG2 cells (Sekiya et al., 2008). Additionally, HepG2 cells exhibit upregulated SCD1 expression upon H_2_O_2_ treatment (Sekiya et al., 2008), and *FASN* expression is upregulated by H_2_O_2_ treatment in human breast cancer cell lines (Furuta et al., 2008). Treatment with the ROS scavenger N-Acetylcysteine (NAC) prevents high glucose-induced increases in FAS activity, 6PGD activity, and TG content in primary yellow catfish hepatocytes (Zhao et al., 2020). Moreover, in mouse adipocytes, *Scd1* and *Acly* are upregulated upon treatment with the superoxide generator TBHP, and downregulated with NAC treatment (Okuno et al., 2018). On the other hand, ROS-augmented (adipose glutathione depleted) mice exhibit increased hepatic TG content, but exhibit decreased gonadal white adipose tissue (WAT) expression of *Fasn*, *Srebf1*, and *Scd1* (Okuno et al., 2018).

Nrf2^−/−^ mice exhibit increased hepatic expression of *Pparα* and *Cpt1* (Tanaka et al., 2012), decreased *Pparγ* (Huang et al., 2010; Tanaka et al., 2008), *Srebf1*(Yates et al., 2009), *Srebf2*(Huang et al., 2010), *Scd1* (Huang et al., 2010), and *Acc* (Huang et al., 2010) expression, as well as decreased liver lipid and TG levels (Huang et al., 2010). However, Nrf2^−/−^ mice have been reported to have both increased (Zhang et al., 2010) and decreased (Huang et al., 2010; Yates et al., 2009) hepatic *Fasn* expression. Likewise, liver *Cd36* expression has been reported to be both increased (Yates et al., 2009) and decreased (Huang et al., 2010) in these mice. H_2_O_2_ treatment decreases *PPARα* and *CPT1* expression in human hepatocytes (Li et al., 2012), and *PPARγ* expression and activity in human umbilical vein endothelial cells (Blanquicett et al., 2010). Moreover, TG accumulation, as well as Srebp1, Fas, and Acc protein expression in mouse adipocytes is dependent on Nrf2 (Sun et al., 2020). Taken together, these data suggest that H_2_O_2_ generally induces genes involved in fatty acid synthesis, while downregulating those involved in FAO, with NRF2 as a key mediator of these effects.

## 6. Advanced glycation end-products and RPE metabolism

AGEs are glycated forms of lipids, proteins and amino acids that accumulate extracellularly during aging. They are implicated in a broad range of pathologies including Alzheimer’s disease (Chaudhuri et al., 2018), Parkinson’s disease (Chaudhuri et al., 2018), kidney disease (Busch et al., 2010), atherosclerosis (Busch et al., 2010), cardiovascular disease (Eganã-gorroño et al., 2020), and diabetes (Eganã-gorroño et al., 2020). AGEs typically form through non-enzymatic glycation by a reducing sugar in a process known as the Maillard reaction, via the polyol pathway or through lipid peroxidation (Ott et al., 2014). Since AGE formation is slow at physiological temperatures, long-lived proteins, particularly ECM components like elastin and collagen, are more susceptible to these modifications (Ott et al., 2014).

In the aging eye, AGEs gradually accumulate on lens crystallin proteins and Bruch’s membrane components, as well as basal deposits and drusen (Farboud et al., 1999; Glenn et al., 2009; Handa et al., 1999). The AGE N^ε^-(carboxymethyl)lysine (CML) is present in soft macular drusen, the RPE, and subretinal membranes in patients with CNV (H.-P. Hammes et al., 1999a; Ishibashi et al., 1998). CML is also found in higher abundance in the retinas of patients with diabetic retinopathy (H. P. Hammes et al., 1999b).

RAGE, the most extensively studied AGE receptor, is a pattern recognition receptor (PRR) and a member of the immunoglobulin superfamily (Teissier and Boulanger, 2019). It recognises ligands that include AGEs (Schmidt et al., 1999), amyloid-β (Yan et al., 1996), as well as DAMPs such as HMGB1 (Hori et al., 1995) and S100/calgranulin family members (Hofmann et al., 1999). RAGE expression in the RPE significantly increases in patients with AMD, particularly in cells overlying basal deposits and drusen (Howes et al., 2004; Park et al., 2015; Yamada et al., 2006). ARPE-19 cells grown on AGE-Matrigel express genes associated with aging, including those related to cell differentiation and apoptosis (Honda et al., 2001). RAGE protein expression increases both with age and the presence of AGEs (Simm et al., 2004; Yu et al., 2013), suggesting that RAGE signalling is likely prominent in the aging RPE.

### 6.1. RAGE signalling and metabolism

RAGE signalling has broad effects on metabolism in different cellular contexts. For example, RAGE signalling alters mitochondrial dynamics through dynamin-related protein 1 (DRP1) in colorectal cancer cells (Huang et al., 2018). Moreover, RAGE-null mice show reduced systemic glucose uptake (Feng et al., 2021), and an approximate 50% decrease in whole-body glycogen synthesis when fed high or low-fat diets (Song et al., 2014). Importantly, the RPE also synthesises and stores glycogen, which is suggested to function as a glucose buffer in the RPE (Kanow et al., 2017; Senanayake et al., 2006). However, the specific effects of increased RAGE on glucose utilisation and glycogen synthesis in the aged or diseased RPE remain to be determined.

Several signalling pathways downstream of RAGE may regulate metabolism. RAGE signalling activates the JAK/STAT, MEK/ERK, JNK, NF-kB pathways (Ott et al., 2014) that regulate metabolism as previously discussed. Indeed, AGE-RAGE interaction has been shown to activate the focal adhesion kinase (FAK) and downstream PI3K/AKT pathways in cultured RPE cells (Sun et al., 2017). RAGE ligand binding also activates the small GTPase Rac1, leading to cytoskeletal reorganisation and possible increase in glycolytic rate due to aldolase mobilisation (Hu et al., 2016; Hudson et al., 2008). RAGE also stimulates expression of the pro-anabolic and glycolytic carbohydrate responsive element binding protein (ChREBP) (Chen et al., 2014). RAGE stimulation may also regulate metabolism indirectly, whether by upregulating cytokines such as TNFα and IL-6 (Singh et al., 2001), or by inducing oxidative stress by activating NADPH oxidase enzymes (Wautier et al., 2001), or inactivation of the antioxidant enzyme, thioredoxin (Liu et al., 2012).

## 7. Growth factor signalling and RPE metabolism

Growth factors are a ubiquitous presence in the blood and tissues of mammals, playing crucial roles in regulating metabolism, typically promoting glucose consumption for the anabolic synthesis of macromolecules. A variety of growth factors reside in the eye, found in the aqueous humour and vitreous, as well as bound to components of the interphotoreceptor matrix (IPM) and Bruch’s membrane. Moreover, many growth factors are upregulated in eye diseases, including AMD. Growth factors such as heparin-binding epidermal growth factor (HBEGF), FGF, and IGF, bind to ECM components. This makes them subject to control by matrix metalloproteinases, a family of proteases that degrade ECM components (Nagase et al., 2006). Several MMPs are upregulated in Bruch’s membrane with aging (Guo et al., 1999) and in CNV (Steen et al., 1998), or are inducible by cytokines such as IL-1β (Eitner et al., 2021; Lee et al., 2002). While the direct effects of growth factors on metabolism have been confirmed in numerous tissues and cell types, there is a paucity of data on the impact of enhanced growth factor signalling on the metabolic ecosystem in the retina.

### 7.1. Epidermal growth factor (EGF)

EGF is a peptide growth factor that promotes cell proliferation, differentiation and survival, particularly of epithelial cells and fibroblasts (Wong and Guillaud, 2004). It is expressed throughout most tissues, with particularly high expression in the kidneys (Kajikawa et al., 1991). EGF and related growth factors, such as heparan-binding EGF (HB-EGF), exert their physiological effects by binding to the epidermal growth factor receptor (EGFR), one of four structurally similar receptor tyrosine kinases (RTKs) (Hynes and Lane, 2005). The EGFR family is widely expressed in cultured cell lines and tissues, including the central nervous system (Wong and Guillaud, 2004), liver (O’Keefe et al., 1974), skin (O’Keefe et al., 1974), and cornea (O’Keefe et al., 1974).

#### 7.1.1. EGF signalling in the retina

Weak staining for EGF and EGFR is observed throughout the retina and RPE (Patel et al., 1994), and both are expressed in cultured RPE cells (Kociok et al., 1998; Yan et al., 2007). EGF maintains homeostasis in this tissue by responding to and repairing retinal damage (Close et al., 2006; Wan et al., 2014). Recent single-cell transcriptomics of the aging primate retina revealed a significant increase in EGFR expression with age across various retinal cell types (Wang et al., 2021), contrasting with age-depended decreases in EGFR expression in other tissues such as the liver and brain (Hiramatsu et al., 1988; Kamat et al., 2008). Several age-related factors may promote EGF signalling in the RPE. HB-EGF expression in primary human RPE cells is inducible by oxidative stress, TGF-β and FGF2 (Hollborn et al., 2006). EGFR signalling may also be induced by HB-EGF liberation from heparin by MMP-2 and -9 (Razandi et al., 2003), which increase in abundance and activity in the aging Bruch’s membrane (Guo et al., 1999).

Increased EGF and EGFR staining is observed in the retinas of patients with PDR (Patel et al., 1994), and inhibition of the EGFR reduces macula oedema (Sugimoto et al., 2013), angiogenesis, and inflammation (Ju et al., 2019) in mouse models of diabetes and diabetic retinopathy. In cultured RPE cells, EGFR activation promotes proliferation (Hollborn et al., 2006; Leschey et al., 1990), EMT (Park and Kim, 2017), and VEGF secretion (Inoue et al., 2018; Park and Kim, 2017) in cultured RPE cells. The latter may contribute to neovascularisation in AMD as knockout of HB-EGF protects against laser-induced CNV in mice (Inoue et al., 2018).

#### 7.1.2. EGF and metabolism

##### 7.1.2.1. EGF and glucose metabolism.

**Fig. 9.**
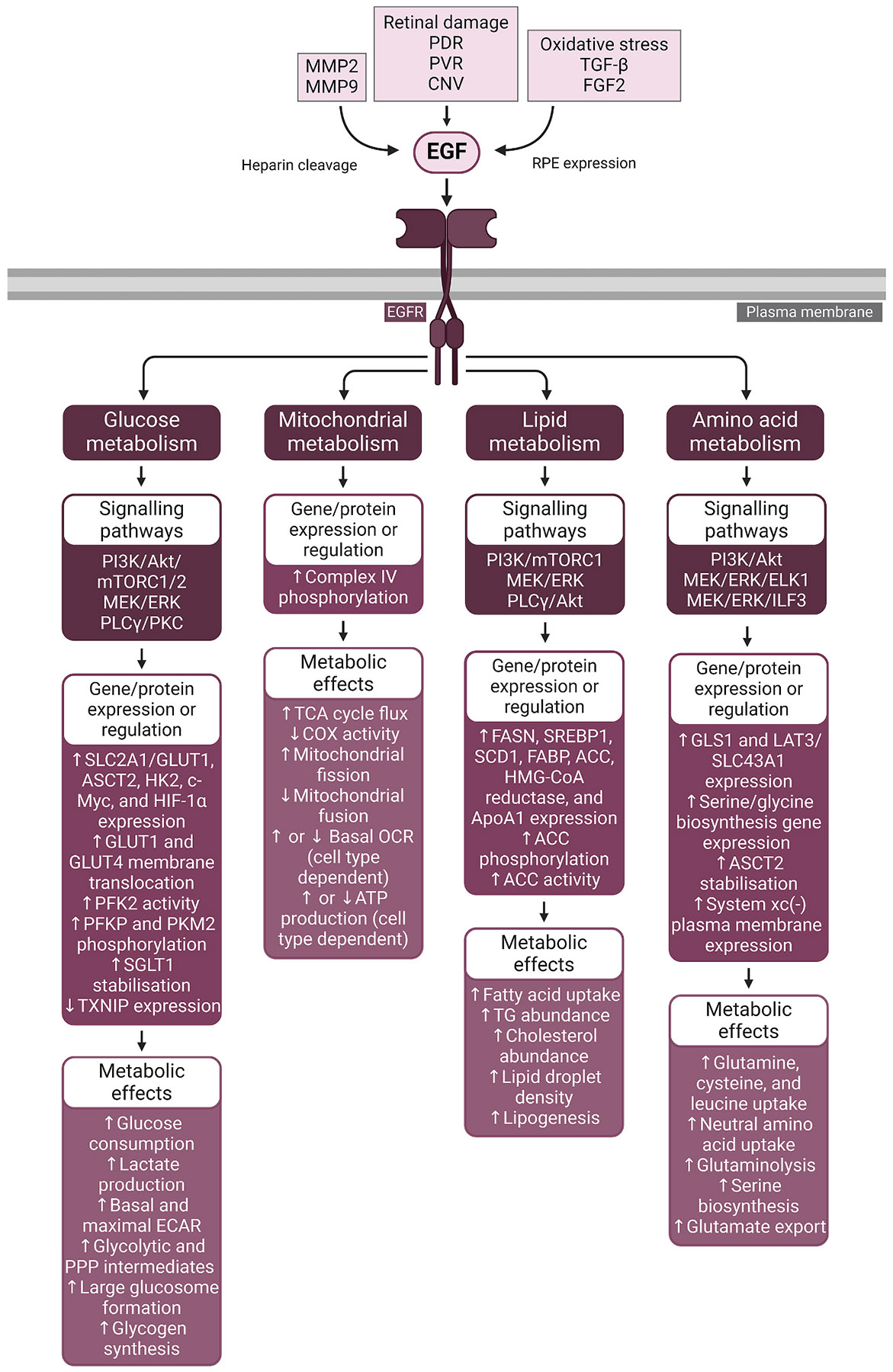
Figure 9. The effects of EGF on metabolism. EGF is expressed in the RPE, can be released from heparin by MMP cleavage, and is upregulated in several eye diseases. EGF acts on several intracellular signalling pathways, influencing metabolic gene/protein expression and activity, and ultimately affecting multiple aspects of glucose, mitochondrial, lipid, and amino acid metabolism. ↑ indicates an increase and ↓ indicates a decrease.

While EGF influences glucose metabolism in a diverse range of cell types, its effects are particularly well studied in cancer models (Fig. 9, Table 6). The EGFR is frequently mutated in cancer, with exon 19 deletions and the exon 21 L858R point mutation commonly associated with NSCLC, while the EGFRvIII variant is a prevalent driver of glioblastomas (Orofiamma et al., 2022). EGFR inhibition decreases *SLC2A1, HK2,* and *c-Myc* gene expression (Makinoshima et al., 2014), and GLUT1, HK2, c-Myc, and HIF-1α protein expression in human NSCLC cells (Momcilovic et al., 2017). Similarly, EGF stimulates GLUT1 protein expression in glioblastoma cells (Lee et al., 2018), and promotes GLUT4 translocation to the plasma membrane in HeLa cells (Trefely et al., 2015). Moreover, EGFR signalling in breast cancer cells increases c-Myc expression (Iqbal et al., 2021), HK2 activity (Jung et al., 2019), and GLUT1 plasma membrane localisation (Jung et al., 2019), while decreasing expression of TXNIP (Iqbal et al., 2021).

The constitutive activation of mutated EGFR in cancer stimulates glucose uptake and lactate production. Glucose consumption is upregulated in EGFRvIII mutant U87 glioblastoma cells (Guo et al., 2009a), and in breast cancer cells treated with EGF (Jung et al., 2019; Kaplan et al., 1990) or that overexpress EGFR (Iqbal et al., 2021). Similarly, inhibition of EGFR signalling decreases glucose consumption and the abundance of glycolytic and PPP metabolites in NSCLC cell lines (Makinoshima et al., 2014; Momcilovic et al., 2017). EGF treatment also increases the basal ECAR in glioblastoma cells (Li et al., 2016), as well as the lactate production (Jung et al., 2019; Lim et al., 2016) and both basal and maximal ECAR in breast cancer cells (Lim et al., 2016). Likewise, EGFR overexpression in breast cancer cells increases lactate production (Iqbal et al., 2021), and inhibition of EGFR tyrosine kinase signalling decreases lactate production and basal ECAR in NSCLC cells (Makinoshima et al., 2014).

The effects of EGFR signalling on cancer glucose metabolism may be driven through the PI3K/AKT/mTORC1 pathway (Jung et al., 2019; Makinoshima et al., 2015), mTORC2 (Masui et al., 2013), the transcription factor c-Myc (Babic et al., 2013; Masui et al., 2013), PKM2 phosphorylation (Lim et al., 2016), or stabilisation of the sodium/glucose cotransporter 1 (SGLT1) (Weihua et al., 2008). EGF treatment also promotes PFK2 activity in squamous cell carcinoma cells, resulting in increased fructose-2,6-bisphosphate abundance (Baulida et al., 1992). Similarly, in glioblastoma cells, activated EGFR directly phosphorylates PFKP, leading to PI3K activation, PFK2 phosphorylation, and increased PFK1 activity (Lee et al., 2018). EGFR signalling also stimulates the MEK/ERK pathway, promoting the formation of large-sized ‘glucosomes’, subcellular assemblies comprising glycolytic enzymes (Jeon et al., 2022). The phospholipase C gamma/protein kinase C (PLCγ/PKC) pathway is also activated by EGFR signalling (Lemmon and Schlessinger, 2010), which regulates glucose metabolism by promoting GLUT4 membrane trafficking (Van Epps-Fung et al., 1997) and by the phosphorylation and inactivation of GSK-3β (Goode et al., 1992).

EGF also regulates glucose metabolism in non-cancer cells. Treatment with EGF promotes glucose consumption in mouse (Barnes and Colowick, 1976; Scimeca et al., 1989) and human (Barnes and Colowick, 1976) fibroblasts, rat adipocytes (Stagsted et al., 1993), rabbit renal proximal tubules (Nowak and Schnellmann, 1995), as well as in primary human RPE cells (Takagi et al., 1994a). EGF also increases lactate production in mouse (Diamond et al., 1978) and rat (Boerner et al., 1985) fibroblasts, as well as in rabbit proximal tubules (Nowak and Schnellmann, 1995). Moreover, EGF promotes glycogen synthesis in rat hepatocytes (Bosch et al., 1986; Freemark, 1986) and mouse adipocytes (Van Epps-Fung et al., 1996). While the effects of EGF on RPE glucose metabolism are unlikely to be as severe as in EGFR-mutated cancer cells, upregulated EGF in the RPE/choroid during aging and AMD may work to increase glycolysis as seen in other non-cancerous cells.

##### 7.1.2.2. EGF and mitochondrial metabolism.

EGF has mixed effects on mitochondrial metabolism depending on cell type. Mouse prolymphocytes with constitutively active EGFR exhibit increased glucose carbon flux into the TCA cycle (Jin et al., 2019). In NSCLC cells, the EGFR translocates to the mitochondria (Che et al., 2015; C.-Y. Huang et al., 2020a), where it inhibits mitochondrial fusion and promotes fission (Che et al., 2015). EGFR inhibition suppresses the basal OCR in these cells (Momcilovic et al., 2017), while EGF treatment increases the mitochondrial reserve capacity (C.-Y. Huang et al., 2020b) and ATP production (Che et al., 2015; C.-Y. Huang et al., 2020b). Conversely, in MEFs, the EGFR phosphorylates respiratory complex IV subunit II (COXII) upon mitochondrial translocation, decreasing COX activity and ATP production (Demory et al., 2009). EGF treatment also decreases the basal OCR in both rabbit renal proximal tubules (Nowak and Schnellmann, 1995) and human glioblastoma cells (Li et al., 2016). Thus, EGF has pleiotropic effects on mitochondrial metabolism, even among cancer or non-cancer cells, and its effects on RPE respiration remain unknown.

##### 7.1.2.3. EGF and lipid metabolism.

Plasma EGF concentrations positively correlate with ApoA1 and HDL-cholesterol concentrations in human plasma (Berrahmoune et al., 2009). Moreover, adult mice with a hyperactive mutant EGFR exhibit increased plasma cholesterol and triglycerides (Scheving et al., 2014). The effects of EGF on lipid metabolism have been particularly well studied in cancer models, where it primarily promotes fatty acid synthesis. EGFR signalling upregulates *FASN* expression in pancreatic cancer cells (Bian et al., 2015), and *SREBF1* (Ali et al., 2018; Chen et al., 2021; Luo et al., 2021; Xu et al., 2021), *FASN* (Ali et al., 2018; Chen et al., 2021; Xu et al., 2021), and *SCD1* (Xu et al., 2021; J. Zhang et al., 2017a) expression in NSCLC cells. In colorectal cancer cells, EGF decreases expression of the fatty acid transporter intestinal fatty acid binding protein (I-FABP) and fatty acid uptake (Darimont et al., 1999) while increasing FAS expression (Wei et al., 2023), and intracellular TG and cholesterol content (Liang et al., 2002). Moreover, EGF increases lipid droplet density in colorectal cancer cells through PI3K/mTOR signalling (Penrose et al., 2016). In glioblastoma cells, EGFR signalling promotes the cleavage and nuclear translocation of SREBP1, inducing FAS and ACC expression (Guo et al., 2009b), with EGFRvIII mutant cells exhibiting increased fatty acid abundance and ACC phosphorylation (Guo et al., 2009a).

EGF has similar lipogenesis-promoting effects in most non-cancer cells. While mice containing an overactive EFGR mutant have decreased hepatic TG, they exhibit increased hepatic cholesterol ester content, and upregulated Fas, and Hmgcr expression (Scheving et al., 2014). Moreover, EGF treatment induces ApoA1 expression in human hepatocytes through the MEK/ERK pathway (Zheng et al., 2001). EGF treatment promotes lipid synthesis from glucose in mouse adipocytes (Van Epps-Fung et al., 1996) and in rat adipocytes (Haystead and Hardie, 1986) as well as increasing ACC activity. EGFR inhibition decreases the expression of *FASN* and *ACC* in human keratinocytes (Oh et al., 2022). Similarly, EGFR overexpression in primary goat mammary epithelial cells increases *Fasn*, *Acc*, and *Scd1* expression, as well as the intracellular TG content through PLCγ1/AKT signalling (J. Huang et al., 2020a). Conversely, EGF treatment decreases total lipid, TG, FFA, and cholesterol concentration in hamster sebocytes (Sato et al., 2001), and EGFR inhibition increases the TG and cholesterol ester abundance, and increases *Srebf1* and *Fas* expression in human sebocytes (Dahlhoff et al., 2015). With the exception of sebocytes, EGF generally promotes fatty acid, TG, and cholesterol biosynthesis in most cells. However, whether these effects are similar in RPE cells remains to investigated.

##### 7.1.2.4. EGF and amino acid metabolism.

EGF is involved in the metabolism and transport of a range of amino acids across many cancer models. EGF treatment promotes the Na^+^-dependent uptake of glutamine in colorectal cancer cells (Ray et al., 2005). Moreover, the EGFR associates with the Na^+^-dependent neutral amino acid transporter ASCT2 in squamous cell carcinoma cells, while EGFR inhibition suppresses glutamine uptake (Lu et al., 2016). In glioblastoma cells, EGF treatment promotes GDH1 expression through the MEK/ERK/ELK1 pathway, stimulating glutaminolysis and incorporation of glutamine carbons into TCA cycle intermediates (Yang et al., 2020). The MEK/ERK pathway is also involved in mediating EGF-stimulated serine biosynthesis in colorectal cancer cells by stabilising interleukin enhancer binding factor 3 (ILF3) (K. Li et al., 2020a). In glioblastoma cells, the EGFR interacts with the system xc(−) transporter, promoting its cell surface expression, glutamate export and cysteine uptake (Tsuchihashi et al., 2016). Inhibition of the EGFR increases aspartate abundance in NSCLC cells (Makinoshima et al., 2014), while EGF treatment promotes leucine uptake in prostate cancer cells by upregulating the L-type amino acid transporter 3 (LAT3) through the PI3K/AKT pathway (Zhang et al., 2019).

EGF may also influence amino acid uptake in non-cancer cells. EGF administration in rats decreases the serum concentration of asparagine, threonine, proline, valine, leucine, and tryptophan, while increasing glycine levels(Thongsong et al., 2002). Moreover, EGF treatment promotes the uptake of alpha-aminoisobutyric acid (AIB), a non-metabolisable surrogate used to study neutral amino acid transport, in chick embryonic hepatocytes (Marino et al., 2000), murine fibroblasts (Scimeca et al., 1989), rat hepatocytes (Bereta et al., 1990), rat kidney cells (Boerner et al., 1985), as well as human trophoblasts (Guyda, 1991). Additionally, murine pro-B-cells containing an overactive EGFR mutant exhibit upregulated expression of serine-glycine biosynthesis pathway genes (Jin et al., 2019). Thus, in both cancer and non-cancer models, EGF regulates several amino acids critical for RPE and retinal function, but its effects in the RPE itself remain poorly defined.

### 7.2. Fibroblast growth factor (FGF)

The FGFs are a family of mitogenic glycoproteins involved in development, wound repair, and tissue homeostasis (Xie et al., 2020). The FGF1 subfamily is the best characterised, consisting of FGF1 and FGF2 which are produced by a variety of cells including macrophages (Yun et al., 2010), smooth muscle cells (Antoine et al., 2005), and endothelial cells (Antoine et al., 2005; Yun et al., 2010). These paracrine FGFs bind ECM components as well as membrane-bound HSPGs, restricting diffusion through the ECM and stabilising the FGF-receptor complex (Schlessinger et al., 2000; Turner and Grose, 2010). FGFs exert their effects by binding to a family of four RTKs, FGFR1-4, most of which are broadly expressed across most tissues, including the retina (Fon Tacer et al., 2010; Turner and Grose, 2010; Valter et al., 2002).

#### 7.2.1. FGF signalling in the retina

HSPGs such as perlecan and agrin are found throughout the retina, including the inner limiting membrane, blood vessel walls, and Bruch’s membrane (Keenan et al., 2012; Lin, 1989), offering potential FGF binding sites. In the eye, both FGF1 and FGF2 are detectable in basement membranes (Jeanny et al., 1987), retina (Caruelle et al., 1989; Connolly et al., 1992; Jacquemin et al., 1990; Walsh et al., 2001), and RPE (Connolly et al., 1992; Jacquemin et al., 1990; Walsh et al., 2001). FGF2 is a potent angiogenic factor in the retina (Wong et al., 2001) and is believed to play a role several ocular pathologies. Diabetic rats exhibit elevated FGF2 mRNA in their eyes compared to non-diabetic rats (Lowe et al., 1995), and vitreous levels of FGF2 protein are elevated in PDR patients (Boulton et al., 1997). FGF2 localises to epiretinal membranes (Hueber et al., 1997) and vascular endothelial cells in diabetic retinopathy (Hanneken et al., 1991). In contrast, FGF1 was found to protect against RPE inflammation and oxidative stress in streptozotocin-induced diabetic rat models (Huang et al., 2021).

FGFs are also associated with neovascularisation in AMD. Patients with CNV display strong expression of FGF1 and FGF2 in the RPE (Amin et al., 1994), while FGF2 is also present in epiretinal and CNV membranes (Frank et al., 1996). RPE and infiltrating macrophages derived from mouse eyes with photocoagulation-induced CNV show increased FGF2 expression (Ogata et al., 1996). FGF2 expression in cultured human RPE cells is inducible by oxidative stress (Hackett et al., 1997; Wada et al., 2009) and the cytokine IL-1β (Hackett et al., 1997).

#### 7.2.2. FGF and metabolism

##### 7.2.2.1. FGF and glucose metabolism.

**Fig. 10.**
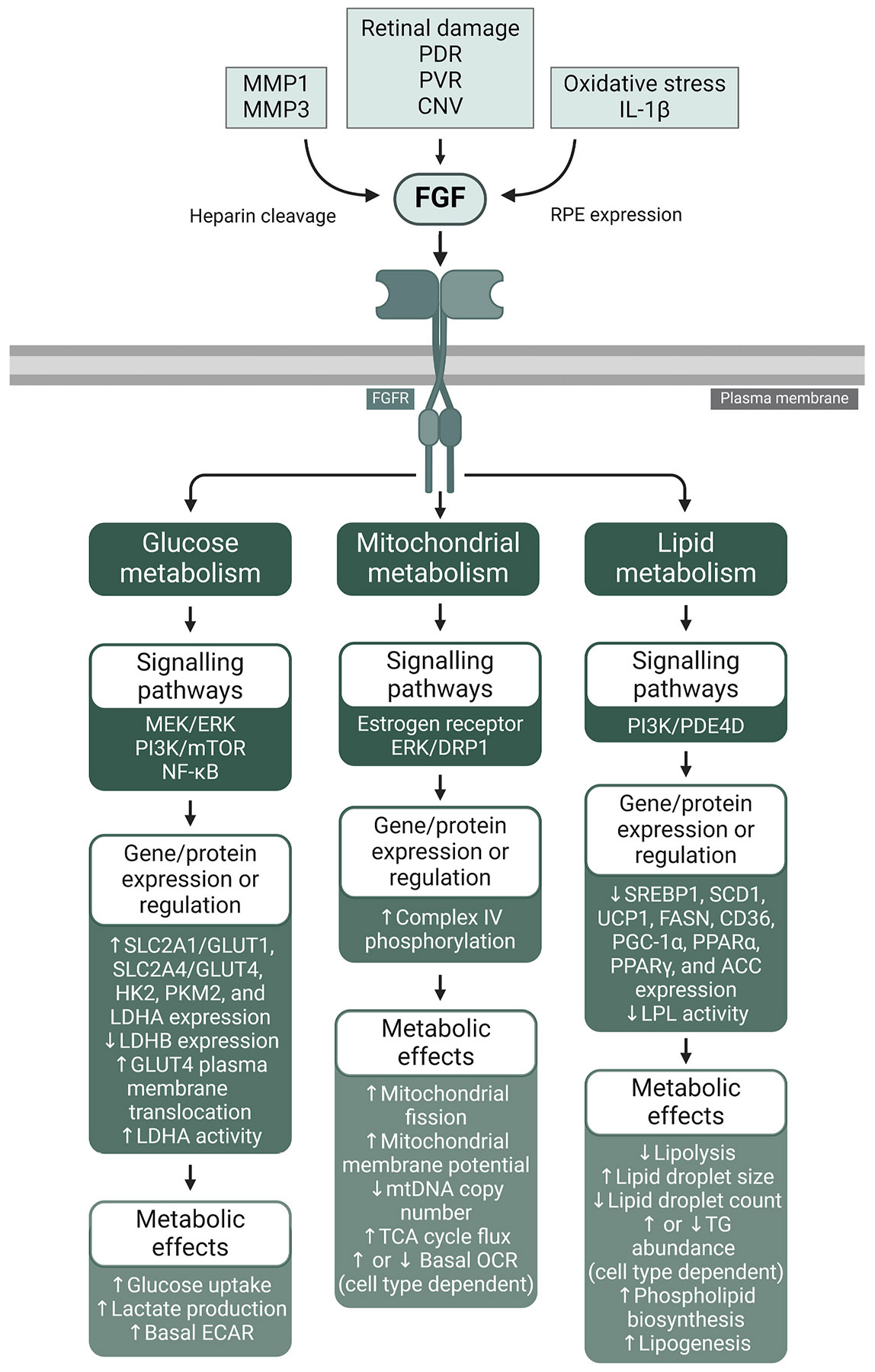
Figure 10. The effects of FGF on metabolism. FGF is expressed in the RPE, can be released from heparin by MMP cleavage, and is upregulated in several eye diseases. EGF acts on several intracellular signalling pathways, influencing metabolic gene/protein expression and activity, and ultimately affecting multiple aspects of glucose, mitochondrial, and lipid metabolism. ↑ indicates an increase and ↓ indicates a decrease.

Both FGF1 and 2 promote glycolysis and glycolytic gene expression across diverse cell types (Fig. 10, Table 7). FGF1 administration promotes Glut4 expression and translocation to the cell surface in the skeletal muscle of diabetic *db/db* mice (Ying et al., 2021). FGF1 signalling through the MEK/ERK pathway promotes *Slc2a1* expression in mouse adipocytes (Nies et al., 2022), while FGF2 promotes GLUT1 protein expression in mouse adipocytes (Kihira et al., 2011). Moreover, FGF2 treatment increases *Slc2a1* and both *Ldha* expression and activity in rat Sertoli cells (Riera et al., 2002). Ablation of *Fgfr1* and *Fgfr2* in MEFs increases the *Ldhb* expression relative to *Ldha* (Liu et al., 2018). Similarly, inhibition of both FGF1 and FGF2 decreases the LDHA/LDHB ratio in prostate cancer cells through the transcription factor STAT1 (Ye et al., 2024). FGFR2 inhibition in patient-derived intrahepatic cholangiocarcinoma (ICC) cell lines containing an overactive FGFR2 mutant decreases *HK2*, *PKM2*, and *LDHA* expression (Zhen et al., 2024).

Both FGF1 and FGF2 promote glucose uptake by human prostate cancer cells (Ye et al., 2024) and mouse adipocytes through the MEK/ERK pathway (Kihira et al., 2011; Nies et al., 2022). Additionally, glucose uptake is promoted by FGF1 in *ex vivo* mouse WAT (Nies et al., 2022) and by FGF2 in rat Sertoli cells (Riera et al., 2002). Moreover, FGFR inhibition in FGFR-mutant ICC cells (Zhen et al., 2024), prostate cancer cells (Liu et al., 2018), as well as in lung squamous cell carcinoma cell lines (Fumarola et al., 2017), decreases glucose uptake. Similarly, inhibition of the PI3K/mTOR pathway suppresses FGF-2-induced glucose uptake in squamous cell carcinoma cells (Fumarola et al., 2017).

FGF1 treatment increases the basal ECAR in breast cancer cell lines (Castillo-Castrejon et al., 2023), while FGF2 treatment increases lactate production in murine adipocytes (Kihira et al., 2011), rat Sertoli cells (Riera et al., 2002), and human squamous cell carcinoma cells (Fumarola et al., 2017). Similarly, inhibition of both FGF1 and FGF2 decreases lactate production, as well as the basal and maximal ECAR in prostate cancer cells (Ye et al., 2024). Moreover, mouse pro-B-cells harbouring an overactive FGFR1 mutant exhibit increased basal ECAR and glucose-derived lactate production (Jin et al., 2019). Similarly, genetic ablation of *Fgfr* isoforms in MEFs and human prostate cancer cells (Liu et al., 2018), as well as FGFR inhibition in FGFR2-mutant ICC cells (Zhen et al., 2024), results in suppressed lactate production. Interestingly, FGFR2-mediated stimulation of glucose uptake, ECAR, lactate production and *HK2* and *LDHA* expression in patient-derived FGFR2-mutant ICC cells is dependent on the transcription factor NF-κB (Zhen et al., 2024). FGFR signalling also activates other signalling pathways implicated in glucose metabolism such as PI3K/AKT, PLCγ/PKC, and JAK/STAT (Turner and Grose, 2010; Xie et al., 2020). Thus, like EGF, both FGF1 and FGF2 increases glycolytic gene expression, glucose consumption, and lactate production in many cancerous and non-cancerous cell types.

##### 7.2.2.2. FGF and mitochondrial metabolism.

Genetic ablation of *Fgfr* isoforms in MEFs and human prostate cancer cells results in increased OCR (Liu et al., 2018), while Fgf2^−/−^ mice exhibit increased WAT and BAT mtDNA copy number (Li et al., 2021). On the other hand, FGF1 treatment increases basal OCR in breast cancer cells through activation of the estrogen receptor (Castillo-Castrejon et al., 2023). FGF1 also increases mitochondrial fission and membrane potential in mouse myoblasts through ERK-mediated phosphorylation of DRP1 (Liu et al., 2024). Mouse pro-B-cells containing an overactive FGFR1 exhibit increased basal OCR (Jin et al., 2019). Moreover, FGFR inhibition in FGFR2-mutant ICC cells results in mitochondrial abnormalities, decreasing the number of fragmented mitochondria while increasing the number of elongated mitochondria, accompanied by decreased glucose carbon labelling of the TCA cycle intermediates citrate, α-KG, and malate (Zhen et al., 2024). Thus, most studies support a role for FGFR signalling in supporting mitochondrial health and metabolism.

##### 7.2.2.3. FGF and lipid metabolism.

In adipocytes, FGF signalling generally supports lipogenesis while downregulating lipid synthesis and oxidation. FGF1 suppresses lipolysis in mouse gonadal WAT explants and cultured mouse adipocytes through PI3K-mediated activation of the phosphodiesterase PDE4D (Sancar et al., 2022). Fgf2^−/−^ mice exhibit decreased WAT lipid droplet size, and increased *Pgc-1α*, uncoupling protein 1 (*Ucp1*), and *Pparγ* expression in both WAT and BAT (Li et al., 2021). Moreover, adipocyte-specific ablation of *Fgfr1* in mice promotes WAT phospholipid biosynthesis and lipogenesis (Ye et al., 2016).

FGFR inhibition in FGFR2-mutant ICC cells results in a higher dependency on fatty acids as a fuel source, decreasing expression of fatty acid synthesis genes while increasing β-oxidation genes, increasing lipase activity, and increasing the number of lipid droplets (Zhen et al., 2024). Moreover, Fgf2^−/−^ mice exhibit decreased liver TG content (Li et al., 2021). On the other hand, FGF1 administration suppresses hepatic *Srebf1*, *Scd1* and *Fasn* expression in *db/db* mice (Lin et al., 2021). Similarly, adipose-specific *Fgfr1* knockout mice exhibit elevated hepatic expression of *Cd36*, *Pparα*, *Pparγ*, *Acc*, *Fasn*, and *Scd1*, and were found to exhibit higher levels of liver TG than wildtype mice following a 48-h fast (Yang et al., 2012). FGF1 treatment of primary mule duck fatty liver cells decreases the TG content, as well as *Acc1* and *Srebf1* expression (Hu et al., 2024). Thus, FGF has conflicting effects on hepatocyte lipid metabolism depending on the experimental model. Whether the FGFs influence lipid synthesis or oxidation in the RPE remains an open question.

### 7.3. Insulin-like growth factor 1 (IGF-1)

IGF-1 shares high structural and functional homology with insulin, regulating growth, differentiation and metabolism (LeRoith and Yakar, 2007). Although it is expressed ubiquitously, IGF-1 is primarily produced in the liver (LeRoith and Yakar, 2007). Extracellular IGF binding proteins (IGFBPs) inhibit IGF-1 by binding to it in circulation with high affinity, restricting its ability to activate the IGF-1 receptor (IGF-1R) (Bach, 2018). MMPs including MMP-1, -2, -3 and -9 can degrade IGFBPs, releasing intact IGF (Coppock et al., 2004; Fowlkes et al., 1995). IGF-1 binds to IGF-1R, a class II RTK with structural similarities to the insulin receptor (LeRoith et al., 1995) which is expressed in RPE cells *in vitro* (Martin et al., 1992) and *in vivo* (Lambooij et al., 2003; Ocrant et al., 1989). The IGF-1 homologue, IGF-2, also binds to IGF-1R (LeRoith and Yakar, 2007), however IGF-1 has closer associations with retinal disease and its influence on glucose metabolism is better characterised.

#### 7.3.1. IGF-1 signalling in the retina

IGF-1 plays well-established roles in retinal development, promoting neurogenesis and vascularisation in the developing neural retina (Hellström et al., 2002; Hernández-Sánchez et al., 1995). Igf1^−/−^ mice exhibit severe deleterious effects on retinal function that intensify with age (Rodriguez-de la Rosa et al., 2012). While IGF-1 grants neuroprotective effects in rodent models of the retinal degenerative disease retinitis pigmentosa (Arroba et al., 2011), overexpression in mouse photoreceptors leads to retinal neurodegeneration and functional decline (Villacampa et al., 2013). IGF-1 is expressed in cultured human RPE cells (Kociok et al., 1998; Takagi et al., 1994b), as well as the RPE *in vivo* (Lambooij et al., 2003).

Diabetic retinopathy patients exhibit elevated serum and vitreous IGF-1 levels (Boulton et al., 1997; Grant et al., 1986; Meyer-Schwickerath et al., 1993), and diabetic rats show increased IGF-1R expression in the retina (Charkrabarti et al., 1991). IGF-1 signalling is also associated with AMD, where patients have significantly higher plasma concentrations of IGF-1 (Machalinska et al., 2011), and a significant association exists between a SNP (rs2872060) in the *IGF1R* gene and advanced AMD (Chiu et al., 2011). Due to immunolocalization of IGF-1 and IGF-R1 to CNV membranes, IGF-1 has been proposed to facilitate neovascularisation in AMD patients (Lambooij et al., 2003; Rosenthal et al., 2004). Indeed, IGF-1 stimulates VEGF secretion from cultured RPE cells (Slomiany and Rosenzweig, 2004).

#### 7.3.2. IGF-1 and metabolism

##### 7.3.2.1. IGF-1 and glucose metabolism.

IGF-1 plays an integral role in maintaining systemic glucose homeostasis, functioning in a similar manner to insulin (Kasprzak, 2021). Treatment with IGF-1 in humans promotes glucose uptake and utilisation by the peripheral tissues while inhibiting hepatic gluconeogenesis (Boulware et al., 1992; Laager et al., 1993; Turkalj et al., 1992). IGF-1 promotes GLUT1 protein expression in human choriocarcinoma cells, syncytial cells, and placental explants (Baumann et al., 2014). It also promotes GLUT4 membrane localisation in cervical cancer cells (Trefely et al., 2015), and trafficking of GLUT1 to the plasma membrane in rat myotubes (Bilan et al., 1992). IGF-1 promotes GLUT1 membrane trafficking in cultured human Müller glial cells through both the PI3K and MEK/ERK signalling pathways (Actis Dato et al., 2021).

**Fig. 11.**
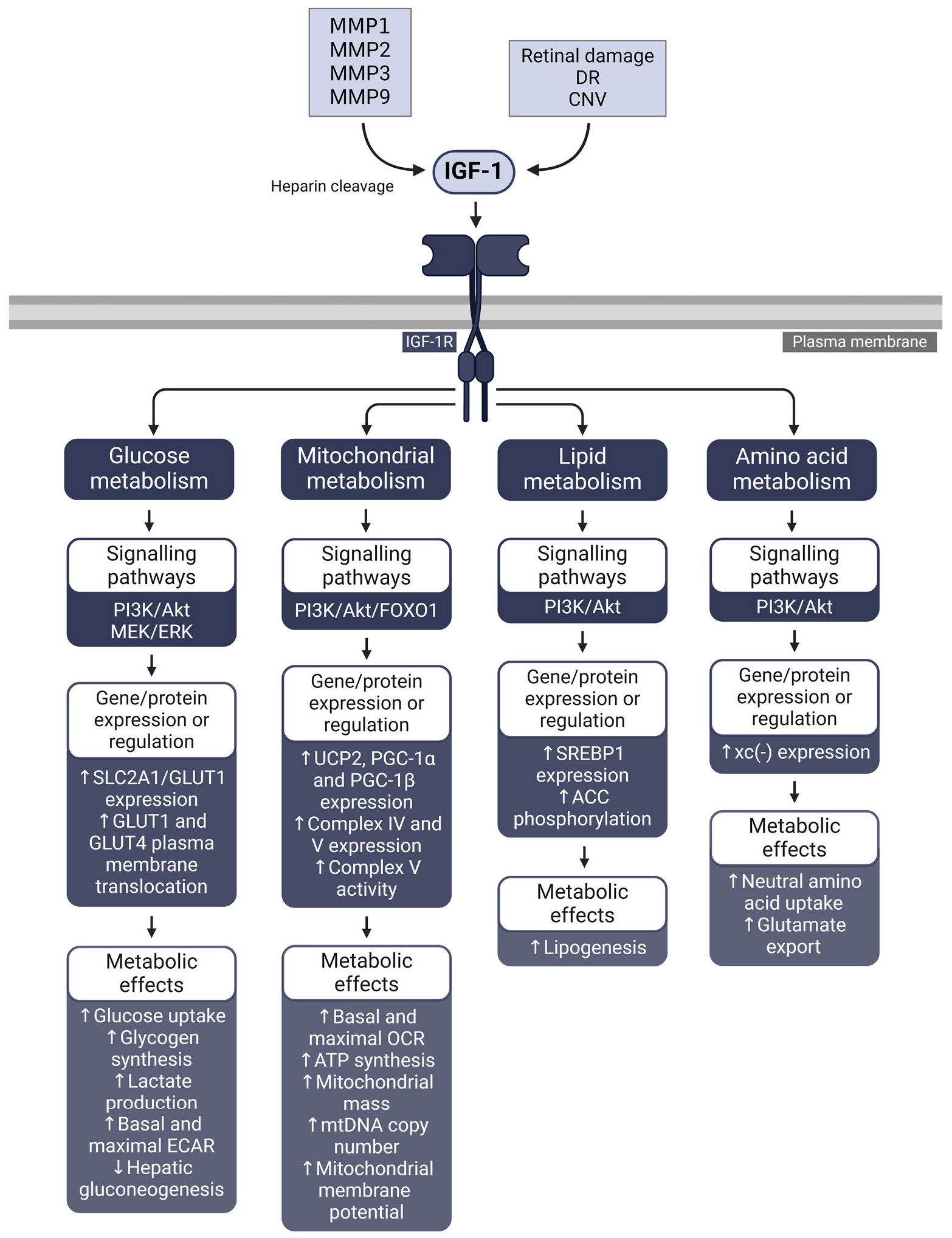
The effects of IGF-1 on metabolism. IGF-1 can be released from heparin by MMP cleavage and is upregulated in several eye diseases. EGF acts on several intracellular signalling pathways, influencing metabolic gene/protein expression and activity, and ultimately affecting multiple aspects of glucose, mitochondrial, lipid, and amino acid metabolism. ↑ indicates an increase and ↓ indicates a decrease.

IGF-1 elicits a robust increase in glucose uptake in a wide range of cell types (Fig. 11, Table 8). Treatment with IGF-1 promotes glucose uptake in mouse adipocytes (Assefa et al., 2017) and CD4^+^ T-cells (Kiernan et al., 2024), rat myotubes (Bilan et al., 1992), *ex vivo* rat (Dimitriadis et al., 1992) and human (Dohm et al., 1990) skeletal muscle tissue, human myotubes (Furling et al., 1999; Henry et al., 1995), choriocarcinoma cells (Baumann et al., 2014), Müller cells (Actis Dato et al., 2021), as well as in cultured hfRPE cells (Takagi et al., 1994a). Similarly, astrocytes (Logan et al., 2018) and whole-brain synaptosomes (Cheng et al., 2000) isolated from Igfr^−/−^ mice exhibit decreased glucose uptake. IGF-1 treatment also promotes glycogen synthesis in *ex vivo* rat skeletal muscle tissue (Dimitriadis et al., 1992). Moreover, IGF-1 increases lactate production in *ex vivo* rat skeletal muscle explants (Dimitriadis et al., 1992) and human multiple myeloma cells (Freund et al., 1993). Similarly, basal and maximal ECAR are increased with IGF-1 treatment in human breast cancer cells (Rajoria et al., 2023) and with IGF-1 overexpression in rat dorsal root ganglion (DRG) neurons (Aghanoori et al., 2022). Like EGF and FGF, IGF signalling stimulates the MEK/ERK pathway (Siddle, 2011), as well as the PI3K/AKT pathway (Siddle, 2011). PI3K signalling is a main driver of IGF-1-induced aerobic glycolysis in mouse mammary tumour cells (Landis and Shaw, 2014), and mediates IGF-1-stimulated glucose uptake in mouse adipocytes (Assefa et al., 2017).

##### 7.3.2.2. IGF-1 and mitochondrial metabolism.

IGF-1 generally promotes mitochondrial health and respiration in most cell types. Treatment with IGF-1 increases the basal and maximal OCR in murine CD4^+^ T-cells (Kiernan et al., 2024) and human embryonic stem cell-derived cardiomyocytes (Sánchez-Aguilera et al., 2023). Moreover, both IGF-1 treatment (Aghanoori et al., 2019) and overexpression (Aghanoori et al., 2022) increases the maximal OCR in rat DRG neurons. These effects in mouse CD4^+^ T-cells and rat DRG neurons are accompanied by increased ATP synthesis. Similarly, primary astrocytes isolated from Igfr^−/−^ mice exhibit decreased basal and maximal OCR (Logan et al., 2018). On the other hand, MCF-7 breast cancer cells with acquired resistance to IGF-1R tyrosine kinase inhibition exhibit reduced basal OCR (Lyons et al., 2017), and silencing of IGF-1R in human colon cancer cells increases the maximal OCR (S. Q. Wang et al., 2019b).

IGF-1 treatment increases the mitochondrial mass (Lyons et al., 2017) and *PGC-1β* expression (Chang et al., 2011; Lyons et al., 2017) in human breast cancer cells through activation of PI3K. Moreover, muscle-specific overexpression of Igf-1 promotes *Pgc-1α* expression in the skeletal muscle of in aged mice (Ascenzi et al., 2019). Similarly, IGF-1 treatment increases mtDNA copy number and *Pgc-1α* expression in mouse myotubes (Guan et al., 2022). Treatment of aged rats with IGF-1 increases liver mitochondrial membrane potential and mitochondrial complex V ATPase activity (García-Fernández et al., 2008; Puche et al., 2008). In addition, IGF-1 increases complex IV and V expression in primary rat DRG neurons (Aghanoori et al., 2019), and MEFs derived from Igf-1R^−/−^ mice exhibit decreased complex V expression (Riis et al., 2020). The effects of IGF-1 on complex V expression may be a compensatory response to mitochondrial uncoupling as IGF-1 induces uncoupling protein 2 (UCP2) expression through the PI3K/AKT/FOXO1 axis in diverse cell types including mouse adipocytes, myoblasts, and human hepatocellular carcinoma cells (Watamoto et al., 2019).

##### 7.3.2.3. IGF-1 and lipid metabolism.

Igf-1^−/−^ mice exhibit increased serum cholesterol (Sjögren et al., 2001), and IGF-1 administration in rats decreases circulating cholesterol (García-Fernández et al., 2008; Pascual et al., 1995) and TG (García-Fernández et al., 2008) levels, while increasing FFAs (García-Fernández et al., 2008). However, IGF-1 administration in humans decreases plasma FFA (Boulware et al., 1992, 1994; Laager et al., 1993; Turkalj et al., 1992), TG (Turkalj et al., 1992), and ketone (Boulware et al., 1994; Laager et al., 1993) levels, and increases the systemic lipid oxidation rate (Hussain et al., 1993). IGF-1 promotes lipogenesis in rat adipocytes (Frick et al., 2000), and induces ACC phosphorylation in human breast cancer cells (Favre et al., 2010). IGF-1 also induces *SREBF1* expression and lipogenesis in human sebocytes through PI3K (Smith et al., 2008). While research has primarily focused on the impact of IGF-1 on systemic lipid metabolism, its effects on RPE lipid synthesis and oxidation remain unknown.

##### 7.3.2.4. IGF-1 and amino acid metabolism.

IGF-1 administration broadly decreases the plasma concentrations of amino acids in rats (Jacob et al., 1989) and humans (Boulware et al., 1994), most consistently influencing leucine, isoleucine, valine, methionine, and phenylalanine concentrations (Boulware et al., 1992; Elahi et al., 1993; Turkalj et al., 1992). These effects are most likely to be attributable to the anabolic effects of IGF-1 as whole-body leucine oxidation decreases with IGF-1 treatment (Laager et al., 1993; Mauras and Beaufrere, 1995). IGF-1 treatment promotes AIB uptake in chicken myotubes (Duclos et al., 1993), *ex vivo* rat skeletal muscle tissue (Dardevet et al., 1994), and human placental trophoblasts (Guyda, 1991; Karl, 1995). IGF-1 also promotes AIB uptake in human choriocarcinoma cells through the PI3K pathway (Fang et al., 2006; Jones et al., 2014). Moreover, IGF-1 treatment promotes system xc(−) expression and glutamate export in several human breast cancer cell lines (Yang and Yee, 2014).

## 8. Biomechanical stress and RPE metabolism

The Bruch’s membrane/choroid complex, with an elastic modulus of approximately 1–2 MPa, is stiffer than other ocular tissues such as the retina, cornea and iris (Ferrara et al., 2021; X. Wang et al., 2018d). The elasticity of this complex in humans declines linearly after the age of 21 at a rate of approximately 1% per year (Ugarte et al., 2006). Mechanical stress strengthens the bonds between ECM components and integrins, cell surface receptors which ‘integrate’ biomechanical information from the extracellular environment in a process called ‘mechano-transduction’. This leads to integrin clustering and focal adhesion formation, ultimately altering the cell’s cytoskeletal architecture (Kechagia et al., 2019). Aging RPE cells display irregular cytoskeletal arrangements, including F-actin stress fibres (Tarau et al., 2019), which are further exacerbated in AMD, potentially due to the mechanical forces applied by drusen (Ach et al., 2015; Mazzitello et al., 2016; Tarau et al., 2019).

### 8.1. Causes of biomechanical stress

The causes of age-related stiffening in Bruch’s membrane have not been well explored experimentally. Stiffness does not change significantly in AMD eyes compared to age-matched controls (Ugarte et al., 2006), suggesting that the mechanism is not AMD-specific. Collagen cross-linking, which drastically increases stiffness in collagen gels (Lin and Gu, 2015), increases slightly with age in Bruch’s membrane (Karwatowski et al., 1995). Aging mice also show decreased expression of proteins involved in elastic fibre assembly (Mori et al., 2019), while decreased thickness/integrity of Bruch’s membrane’s elastic layer is associated with CNV (Chong et al., 2005). Decreasing elastin content in Bruch’s membrane during aging could also contribute to stiffness, but this hypothesis requires further experimental validation.

### 8.2. Mechanotransduction and metabolism

Integrins themselves may directly augment growth factor signalling pathways; for example, β1 integrin-mediated adhesion activates the EGFR in multiple cell types (Moro et al., 1998, 2002). Moreover, matrix-integrin interactions trigger the autophosphorylation of focal adhesion kinase (Frisch et al., 1996), which subsequently activates several pro-glycolytic signalling pathways such as PI3K/AKT and MAPK/ERK (Mitra et al., 2005).

Glucose metabolism may also be regulated through cytoskeletal dynamics. The glycolytic rate in human bronchial epithelial cells increases when plated on collagen to mimic a stiff extracellular environment (Park et al., 2020). In these cells, F-actin stress fibres that form under stiff conditions bind to and sequester the E3 ubiquitin ligase, tripartite motif containing 21 (TRIM21). TRIM21 targets PFK, a rate limiting enzyme of glycolysis, for degradation (Lee et al., 2017). Consequently, F-actin restricts TRIM21 motility, leading to increased PFK stability and glycolysis. Cytoskeleton reorganisation has been shown to increase glycolysis through another mechanism in mammary epithelial cells MCF10A cells. Activation of PI3K disrupts the actin cytoskeleton via the GTPase, Rac (Hu et al., 2016), releasing F-actin–bound aldolase and resulting in increased glycolysis. The data highlight several avenues through which altered biomechanical properties and cytoskeletal changes may influence metabolism in the RPE.

## 9. Conclusions and perspectives

The aging RPE and Bruch’s membrane are plagued by a range of pathological conditions, some unique and others common to aging tissues, that can influence RPE metabolism. These include chronic, nonresolving inflammation driven by DAMPs, cellular senescence, basal deposits, and drusen; oxidative stress exacerbated by high OXPHOS, photoreceptor OS phagocytosis, lipofuscin, incoming solar radiation and declining antioxidant capacity; accumulation of AGEs in Bruch’s membrane with increased expression of their receptor in the RPE; and dysregulated MMP activity in Bruch’s membrane, potentially affecting growth factor signalling pathways. Additionally, aging is associated with increasing biomechanical stress placed upon the RPE by basal deposits, drusen, and a stiffening Bruch’s membrane.

Furthermore, pathologies such as AMD have strong associations with chronic inflammation, oxidative stress, growth factor signalling, and metabolic dysregulation. These pathological features may converge to cause metabolic dysregulation in the RPE, compromising the ability of these cells to perform the critical function of facilitating metabolite exchange between the choroid and outer retina. More research is needed to fully elucidate the complex relationship between these disease states, factors such as cytokines, growth factors, and free radicals, and RPE metabolism.

Advancements in gene editing technology have vastly increased the efficacy of generating animal models to study how single genes or pathways affect different pathologies (Lee et al., 2020). Mice where specific genes encoding antioxidant, metabolic, and inflammatory components have been knocked out have proved to be powerful tools for understanding the cause and progression of AMD (Pennesi et al., 2012). Moreover, cell-specific promotors have been used to knock out genes specifically in photoreceptors (Cheng et al., 2020; Dong et al., 2009; Petit et al., 2018; Zhou et al., 2015) and RPE cells (Chuang et al., 2022; Shao et al., 2022; Y. Zhang et al., 2017a). These animal models have provided an invaluable tool for elucidating not only the role of particular genes in retinal disease, but also in deciphering their essential functions in specific retinal cell types. Given our emerging understanding of the association between growth factors, cytokines, and other factors with the pathogenic features of aging and disease, animal models such as these present an invaluable resource, where essentially any gene can be knocked out specifically in defined cell types or tissues, even under temporal control. However, while these animal models allow the roles of specific genes in retinal development and visual function to be determined, distinguishing the specific effects on metabolism in distinct retinal cell populations, and the overall retinal metabolic ecosystem *in vivo* presents more difficult challenges.

Our understanding of the retinal metabolic landscape has expanded rapidly over recent years. Advances in mass spectrometry technology now enable the detailed examination of the ‘metabolome’, the application of which has recently been applied to bulk retina and RPE metabolomics (B. Li et al., 2020b; Sinha et al., 2020b; Zhang et al., 2021). The integration of radiolabelled metabolite treatments of cultured RPE cells (Chao et al., 2017; Du et al., 2016; Yam et al., 2019) and explanted retinal tissue (Chao et al., 2017; Hass et al., 2022; Kanow et al., 2017; Yam et al., 2019) has provided insight into how the retina utilizes nutrients, and highlighted how key metabolites are exchanged between specific cell types. While the metabolic examination of *ex vivo* tissue and cultured cells provide useful insights, there are drawbacks inherent to the removal of these cells from their native state.

The evolution of the field of retina metabolism is moving towards *in vivo* analysis of metabolites, allowing the measurement of real-time changes in flux in response to genetic and chemical perturbations. The refinement of existing and emergence of novel technologies capable of detecting metabolite abundance within living organisms is beginning to transform both how retinal metabolism is analysed, and the quality, precision and accuracy of data obtained.

Magnetic resonance spectroscopy (MRS) is a technique that combines the principles of nuclear magnetic resonance (NMR) with detection using an MRI scan. This non-invasive method has been useful tool in obtaining a rudimentary picture of the metabolic landscape of tumours and the brain (Gillies and Morse, 2005; Van Der Graaf, 2010). However, while it continues to be a powerful tool it currently suffers from the drawback of low sensitivity (Van Der Graaf, 2010) and resolution (Buonocore and Maddock, 2015). Although these factors are set to improve with advances in ultra-high magnetic field strengths and coil technology (Vachha and Huang, 2021), the resolution is still insufficient to detect metabolic changes in specific regions of the retina, much less between individual retinal cells.

Microdialysis combined with mass spectrometry is another powerful metabolomic technique, useful for monitoring changes in neurotransmitters, neuromodulators, and other metabolites in brain extracellular and cerebral spinal fluid (Bongaerts et al., 2018; Kao et al., 2015). This technique offers the advantage of allowing continuous fluid sampling from live animals. Microdialysis of vitreous fluid adjacent to the retina has been used to monitor glutamate release in response to ischemia in mice (Louzada-Junior et al., 1992), and 17-β glucuronide in rats (Katayama et al., 2006). Although this technique is not capable of discerning metabolic differences between individual cells of the retina, it can provide valuable insights into how different conditions affect the production and secretion of metabolites *in vivo*, and the transport of specific metabolites across the BRB.

A key improvement in mass spectrometry has been the development of mass spectrometry imaging (MSI), which provides the spatially resolved distribution of metabolites in tissues. This has facilitated the progression of the technology from bulk, low resolution multi-cell analysis of tissues to proteomics and metabolomics at single cell resolution (Gilmore et al., 2019; Taylor et al., 2021). While numerous MSI techniques exist, analysis of biological samples generally involves the ionisation of metabolites in tissue sections, which are subsequently measured by the mass spectrometer. The spatial resolution of MSI is constantly increasing, now capable of better than 2 μm resolution, and is of detecting metabolites with abundance in the femtomole to attomole range using secondary ion mass spectrometry (SIMS) (Gilmore et al., 2019; Taylor et al., 2021). MSI has been used to detect metabolites in porcine retinas (Sun et al., 2014), as well as retinal lipids in mice (Anderson et al., 2014). Surprisingly, aside from these studies, which are almost a decade old, the application of MSI in retina research has been limited. Although innovations such as gas cluster ion beams (GCIBs) have considerably increased the sensitivity, current techniques have very low ionisation efficiencies which vary between metabolites, leaving considerable room for improvement (Gilmore et al., 2019). As this technology continues to develop, it is predicted that the unique metabolism of the RPE and all the other cells in the retina will be characterised at a single cell level *in vivo* for all of the major metabolites and key pathways.

When combined with genetic knockouts and other animal models, these techniques will provide a powerful tool to decipher how metabolic exchange in the retina is disrupted by factors involved in retinal aging and disease such as oxidative stress, a stiff Bruch’s membrane, AGEs, as well as specific growth factors and cytokines. Improved technology should enable the analysis of other important metabolites beyond glucose metabolism, OXPHOS, and related pathways such as the PPP by identifying changes in components such as lipids or amino acids. Establishing the link between the aging and disease-relative factors and metabolic changes will not only aid our understanding of retinal pathology, but may reveal therapeutic targets such as key factors, metabolites, and signalling components. Thus, furthering our understanding of these processes is of key value as we move towards better clinical outcomes in aging and disease.

## Figures and Tables

**Table 1:** The effects of TNFα on metabolism in different experimental systems.

Organism	Tissue	Cell line/model	Treatment	Effect on metabolism^1^	Signalling pathway	Reference
Hamster	Plasma	Syrian hamster	Intraperitoneal rhTNFα injection	↓Plasma LCAT activity	NA	Ly et al. (1995)
Hamster	Various tissues	Syrian hamster	Intraperitoneal rhTNFα injection	↓Muscle, heart, intestine stomach and kidney CETP	NA	Hardardóttir et al. (1996)
Human	Adipose	Primary adipocytes	TNFα	↑Lipolysis	MEK/ERK	Rydén et al. (2002)
Human	Bone	OST osteosarcoma	rhTNFα	↓LPL activity ↓TAG oxidation	NA	Sakayama et al. (1996)
Human	Cartilage	Primary chondrocytes	TNFα	↓Complex I activity ↓Mitochondrial membrane potential ↓ATP	NA	López-Armada et al. (2006)
Human	Immune system	THP-1 monocytes	TNFα	↓ABCA1 ↑Intracellular cholesterol ester ↓Extracellular total cholesterol	NA	Voloshyna et al. (2014)
Human	Immune system	THP-1 monocytes	TNFα	↓Abca1 ↑Intracellular total cholesterol	NA	Mei et al. (2007)
Human	Liver	HepG2 hepatoma	rhTNFα	↓ApoA1, ApoB, TG, cholesterol, LCAT	NA	Ettinger et al. (1994)
Human	NA	Primary fibroblast-like synoviocytes	rhTNFα	↑*SLC2A1, PFKL, HIF1A* ↑Basal and maximal ECAR	TAK1	Koedderitzsch et al. (2021)
Human	Pancreas	PANC-1 pancreatic cancer cells	rhTNFα	↑Glucose consumption ↑Lactate production ↑*SLC2A3, PDK1* ↓*PDK2, PDK3*	NA	(M. Liu et al., 2016b)
Human	Plasma	NA	rhTNFα infusion	↑Plasma glycerol and FFA	NA	Plomgaard et al. (2005)
Human	Plasma	Rheumatoid arthritis patients	Anti-TNFα antibody treatment	↑HDL-cholesterol	NA	Popa et al. (2005)
Human	Plasma	Rheumatoid arthritis patients	Anti-TNFα antibody treatment	↑Total cholesterol ↑HDL-cholesterol	NA	Vis et al. (2005)
Human	Prostate	LNCaP	TNFα	↓GLUT1 ↑Basal and maximal ECAR ↑Glycolytic ATP production ↓Mitochondrial content ↓Maximal OCR ↓PGC-1α expression ↓Cytochrome C expression ↓Total ATP	NA	Vaughan et al. (2013)
Human	Prostate	RWPE-1	TNFα	↑GLUT1 ↑Basal and maximal ECAR ↑Glycolytic ATP production ↓Mitochondrial content ↓Basal and maximal OCR ↓Total ATP	NA	Vaughan et al. (2013)
Human	RPE	ARPE-19	rhTNFα	↑Lactate production ↑Glucose consumption ↓SLC2A1 ↓LDHB ↑Basal and maximal ECAR ↑Maximal OCR	NA	Hansman et al. (2024)
Human	RPE	hfRPE	rhTNFα	↓Basal and maximal ECAR ↑Basal and maximal OCR ↑ATP ↓PGC-1α ↑Mitochondria size ↑mtDNA copy number	NF-κB	Shu et al. (2022)
Human	RPE	hfRPE	rhTNFα	Alters amino acid metabolism pathways	NA	Ng et al. (2023)
Monkey	Plasma	Cynomolgus monkey	rhTNFα injection	↑Plasma TAG ↓Plasma cholesterol and ApoB ↓Plasma LCAT activity	NA	Ettinger et al. (1992)
Monkey	Plasma	Cynomolgus monkey	rhTNFα injection	↓Plasma LCAT activity	NA	Ettinger et al. (1990)
Mouse	Adipose	30A-5 preadipocytes	rhTNFα	↓ACC activity	NA	Pape and Kim (1988)
Mouse	Adipose	3T3-L1	rhTNFα	↓Glut1, Glut4, C/ebp ↑Insulin resistance	NA	Stephens and Pekala (1991)
Mouse	Adipose	3T3-L1	TNFα	↓*Slc2a4, Pparγ*	NA	Rotter et al. (2003)
Mouse	Adipose	3T3-L1	rhTNFα	↓LPL activity ↑Lipolysis	NA	Kawakami et al. (1987)
Mouse	Adipose	3T3-L1	rmTNFα	↑Lipolysis	NA	Souza et al. (1998a)
Mouse	Adipose	3T3-L1	rmTNFα	↑Lipolysis	PPARγ	Souza et al. (1998b)
Mouse	Adipose	3T3-L1	rmTNFα	↑Lipolysis ↓Lipid content ↓HSL, PPARα, PPARγ, SREBP1, FABP4, LPL, FASN, SCD1	NA	Jack et al. (2022)
Mouse	Adipose	3T3-L1	rmTNFα	↓ACS, CD36, LPL, HSL, GLUT4	NA	Tamori et al. (2002)
Mouse	Adipose	3T3-L1	TNFα	↓HSL, ATGL	NA	Kralisch et al. (2005)
Mouse	Adipose	3T3-L1	TNFα	↓PPARγ	NA	Xing et al. (1997)
Mouse	Adipose	3T3-L1	rmTNFα	↓PPARγ ↓C/EBPα	NA	Zhang et al. (1996)
Mouse	Adipose	3T3-L1	TNFα	↓GLUT4, HSL, C/EBPα, G3PDH	NF-κB	Ruan et al. (2002a)
Mouse	Adipose	3T3-L1	mTNFα	↑Lipolysis	β-arrestin/ERK	Kawamata et al. (2007)
Mouse	Adipose	3T3-L1 and 3T3-F442A adipocytes	mTNFα	↓Glut4 ↑Insulin resistance	NA	Hotamisligil et al. (1994)
Mouse	Adipose	Primary adipocytes	rmTNFα	↑Lipolysis	NA	Sethi et al. (2000)
Mouse	Fibroblasts	L929	rmTNFα	↓Basal OCR ↑Mitochondrial structural abnormalities	NA	Schulze-Osthoff et al. (1992)
Mouse	Immune system	J774 macrophages	rTNFα	↓*Abca1, Abcg1*	NA	Khovidhunkit et al. (2003)
Mouse	Immune system	J774.2 macrophages	rmTNFα	↓LPL activity	NA	Tengku-Muhammad et al. (1998)
Mouse	Immune system	J774.2 macrophages	TNFα	↓LPL activity and expression	PI3K	Tengku-Muhammad et al. (1999)
Mouse	Immune system	Primary peritoneal macrophages	rmTNFα	↑Abca1 ↑Cholesterol efflux	NF-κB, p38 MAPK	Gerbod-Giannone et al. (2006)
Mouse	Liver	C57BL/6J	Intramuscular hTNFα injection	↑Hepatic FAS ↑Hepatic citrate	NA	Grunfeld et al. (1990)
Mouse	Liver	H2.35	TNFα	↓Mitochondrial membrane potential ↓ATP	NA	Samavati et al. (2008)
Mouse	Liver	NMRI	rhTNFα tail vein injection	↓Blood glucose ↓Hepatic glycogen	NA	Mahony and Tisdale (1990)
Mouse	Liver	Primary hepatocytes	TNFα	↑Basal OCR ↓ATP	NA	Kastl et al. (2014)
Mouse	Muscle	C2C12 myoblasts	TNFα	↑*Slc2a1, Slc16a3, Hk2, Pfk, Ldha, Hif1a* ↑Glucose consumption ↑Lactate production	NF-κB, HIF-1α	Remels et al. (2015)
Mouse	Muscle	C2C12 myotubes	rhTNFα	↓Basal OCR ↓FAO	PIKE-A, AMPK	Tse et al. (2017)
Mouse	NA	L929 fibrosarcoma cells	TNFα	↑*Pfk* and *Gapdh* ↑Glucose consumption ↑Lactate production ↑Glutaminolysis ↓COX II and ATPase	NA	Sánchez-Alcázar et al. (2003)
Mouse	Neurons	HT-22	rmTNFα	↓Basal OCR ↓ATP	NA	Doll et al. (2015)
Mouse	Serum, adipose	C57BI/6	Intraperitoneal mTNFα injection	↑Serum TG ↓Adipose LPL activity	NA	Feingold et al. (1994)
Mouse	Serum, liver	C57BI/6	Intraperitoneal mTNFα injection	↑Serum TG and cholesterol ↑Hepatic HMG-CoA reductase activity	NA	Memon et al. (1993)
Mouse	Skeletal muscle	C57BL/6	TNFα infusion	↓Glucose uptake ↓AMPK activity ↓ACC phosphorylation ↓FAO ↑DAG and TAG content ↑Insulin resistance	AMPK, PP2C	Steinberg et al. (2006)
Mouse, Bovine	Liver	Liver homogenates	TNFα	↓Complex IV activity ↓ATP	NA	Samavati et al. (2008)
Pig	Testes	Primary Sertoli cells	TNFα	↑*Ldha*	NA	Boussouar and Benahmen (1999)
Rat	Adipose	Adipose tissue explants	rhTNFα	↑Lipolysis	NA	Porter et al. (2002)
Rat	Adipose	Primary adipocytes	rhTNFα	↑Lactate production ↑Lipolysis	NA	Porter et al. (2002)
Rat	Adipose	Primary adipocytes	rhTNFα	↑Lipolysis	NA	Green et al. (1994)
Rat	Adipose	Primary adipocytes	rhTNFα	↓PPARγ ↓GLUT4	NA	Tanaka et al. (1999)
Rat	Heart	Isolated perfused rat hearts	rmTNFα	↑Lactate production	NA	Hülsmann and Dubelaar (1988)
Rat	Heart	Primary cardiomyocytes	rmTNFα	↓LPL activity	NA	Hülsmann and Dubelaar (1988)
Rat	Heart	Primary cardiomyocytes	rhTNFα	↓Basal OCR ↓PDH activity ↓Complex I and II activity	NA	Zell et al. (1997)
Rat	Heart	Primary cardiomyocytes	TNFα	↓mtDNA copy number ↓Complex III activity	ROS	Suematsu et al. (2003)
Rat	Liver	H35 hepatoma	rhTNFα	↓LCAT activity	NA	Ly et al. (1995)
Rat	Liver	Primary hepatocytes	hTNFα	↓FAO	NA	Nachiappan et al. (1994)
Rat	Liver	Sprague-Dawley	rhTNFα tail vein injection	↓Hepatic peroxisomal β-oxidation enzymes	NA	Beier et al. (1992)
Rat	Liver	Sprague-Dawley	TNFα	↑Hepatic membrane vesicle-mediated uptake of MeAIB, Gln, and Cys	NA	Pacitti et al. (1993)
Rat	Liver	Sprague-Dawley isolated perfused liver	rhTNFα	↑Hepatic Asp, Tyr and Phe uptake ↓Hepatic Asn, Gln and Ala uptake ↓Hepatic Glu release ↓Hepatic FFA uptake and β-HB release ↑Hepatic glucose uptake ↑Hepatic Gly, Val, and Ile content	NA	De Bandt et al. (1994)
Rat	Liver	Wistar	Tail vein rhTNFα injection	↑Hepatic Ala, Ser, Glu, Arg, Pro, Lys and Thr uptake ↓Hepatic Leu, Iso, and Val uptake	NA	Argiles and Lopez-Soriano (1990)
Rat	Liver	Wistar isolated perfused liver	rhTNFα	↑Hepatic Val and Iso uptake ↓Asp uptake	NA	Holeček et al. (1997)
Rat	Muscle	L6 myotubes	TNFα	↓AMPK activity ↓ACC phosphorylation ↑Insulin resistance	AMPK, PP2C	Steinberg et al. (2006)
Rat	Plasma	Wistar	Tail vein rhTNFα injection	↓Plasma Ser, Gly, Ala, Pro, Trp, and His	NA	Argiles et al. (1989)
Rat	Plasma	Wistar	rhTNFα infusion	↑Systemic leucine turnover, oxidation and clearance ↑Plasma Trp and ornithine	NA	Holeček et al. (1997)
Rat	Plasma, liver	Sprague-Dawley	Tail vein rhTNFα injection	↑Plasma TG ↑Hepatic TG synthesis	NA	Feingold et al. (1989a)
Rat	Plasma, liver	Sprague-Dawley	Tail vein rhTNFα injection	↑Plasma TG, glycerol, FFA ↑Hepatic fatty acid synthesis	NA	Feingold et al. (1990)
Rat	Plasma, liver	Sprague-Dawley	rhTNFα tail vein injection	↑Hepatic FAS and ACC activity ↑Hepatic citrate ↓Hepatic fatty acyl-CoA ↑Plasma FFA	NA	Grunfeld et al. (1988)
Rat	Plasma, liver, adipose	Wistar	TNFα infusion	↑Plasma FFA and TG ↑Adipose aldolase ↓Adipose GLUT4, LPL and FAS ↑Hepatic FAS and SREBP1	NA	Ruan et al. (2002b)

1. rhTNFα = recombinant human TNFα, rmTNFα = recombinant mouse TNFα, mTNFα = mouse TNFα, rTNFα = recombinant TNFα.

2. ↑ indicates and increase, ↓ indicates a decrease.

**Table 2:** The effects of TGF-β on metabolism in different experimental systems.

Organism	Tissue	Cell line/model	Treatment^1^	Effect on metabolism^2^	Signalling pathway	Reference
Human	Brain	T98G and U87MG glioblastoma cells	rhTGF-β1	↑*GLUT1, HK2, LDHA, PFKFB3* ↑Lactate production, glucose consumption	Smad, p38MAPK, PI3K	Rodríguez-García et al. (2017)
Human	Breast	MCF10A breast epithelial cells	rhTGF-β1	↓*SLC3A2* ↓Leucine uptake	NA	Loayza-Puch et al. (2017)
Human	Breast	MCF-7 breast epithelial carcinoma cells	rhTGF-β1	↑Glucose consumption, lactate production ↑*GLS1* ↓Basal OCR	DLX-2	Lee et al. (2016)
Human	Breast	MCF-7 breast epithelial carcinoma cells	rhTGF-β1	↑*CD36, CPT1,* COX1 ↓*FASN, ACC* ↑ATP	AMPK	Liu et al. (2020)
Human	Breast	MCF-7 breast epithelial carcinoma cells	TGF-β1	↑GLUT1 ↑Glucose consumption	NA	Li et al. (2013)
Human	Breast	MDA-MB-231 and MCF-7 breast epithelial carcinoma	rmTGF-β1	↓GLUT1 ↓Glucose uptake	NA	Nilchian et al. (2020)
Human	Cartilage	Primary chondrocytes	TGF-β1	↑SLC2A1, ↑HK1, ↑HK2 ↑Glucose consumption, lactate production ↓ATP production	NA	(C. Wang et al., 2018d)
Human	Cervix	SiHa squamous cell carcinoma cells	rhTGF-β2	↑*CD36* ↑Fatty acid uptake ↑FAO ↑Lipid droplet formation	PKC	Corbet et al. (2020)
Human	Fibroblasts	Primary fibroblasts	TGF-β1	↑*PFKBP3, LDHA, SLC2A1* ↑Lactate production ↑Basal ECAR ↑Basal and maximal OCR ↑Serine-glycine biosynthesis pathway genes	mTORC1/2, Smad3, ATF4	Selvarajah et al. (2019)
Human	Immune system	Activated effector memory CD4^+^ T-cells	TGF-β1	↓Basal and maximal ECAR ↓Basal OCR ↓Complex V activity	Smad2	Dimeloe et al. (2019)
Human	Liver	Hep3B hepatocellular carcinoma cells	Long-term rhTGF-β1 treatment	↓Basal ECAR ↓Lactate production ↑Basal and maximal OCR ↑*FABP1, FABP3, SLC27A1, SLC27A4, SLC27A5, ACSL3, ACSL5, HMGCS1* ↑Lipolysis	NA	Soukupova et al. (2021)
Human	Liver	PLC/PRF/5 and SNU449 hepatocellular carcinoma cells	Long-term rhTGF-β1 treatment	↑*LDHA, GLS1* ↓*LDHB* ↑Glutaminolysis ↓PLC/PRF/5 basal and maximal OCR ↑SNU449 maximal OCR	NA	Soukupova et al. (2017)
Human	Lung	A549 NSCLC cells	rhTGF-β1	↑PKM2	mTOR	(Y. Liu et al., 2016a)
Human	Lung	A549 NSCLC cells	rhTGF-β	↑*P4HA3* ↑Glu, Asp ↓Ala, Gly, Asn, Gln, Cit, Pro, Hydroxyproline	NA	Nakasuka et al. (2021)
Human	Lung	IMR-90 fibroblasts	Porcine platelet-derived TGF-β1	↑GLS1 ↑Glutaminolysis ↑TCA metabolites, Glu ↑Basal OCR ↑HIF-1α	Smad3, p38 MAPK	Bernard et al. (2018)
Human	Lung	Primary fibroblasts	TGF-β	↑*PGC-1α* ↑Mitochondrial mass	Smad2/3	Sun et al. (2019)
Human	Pancreas	Panc1 ductal carcinoma	rhTGF-β	↑*SLC21A1, SLC2A3* ↓*PDK3, PDK4* ↑Glucose consumption, lactate production	NA	(M. Liu et al., 2016a)
Human	Pancreas	Panc1 ductal carcinoma	TGF-β1	↑*PFKFB3* ↑Glucose consumption, lactate production ↑Fructose 2,6-BP	NA	Yalcin et al. (2017)
Human	Peritoneum	Primary mesothelial cells	rhTGF-β1	↑SLC2A1, LDHA, PDK1, HIF1A ↑Glucose consumption, lactate production	NA	Young et al. (2014)
Human	Retina	hfRPE	rhTGF-β2	Altered fatty acid biosynthesis pathways	NA	Ng et al. (2023)
Human	Retina	Primary RPE cells	rhTGF-β1,2,3	↑*SCD*	NA	Samuel et al. (2002)
Human	RPE	ARPE-19 cells	TGF-β2	↑*SLC2A1, LDHA* ↑Glucose consumption, lactate production	PI3K/AKT	Hansman et al. (2024)
Human	RPE	ARPE-19 cells	rhTGF-β2	↑*SLC2A3, SLC16A1, LDHA, PKM2, PFKFBP3* ↑Maximal ECAR ↑Glucose consumption ↓*PGC-1α* ↓Maximal OCR ↓ATP	NA	Shu et al. (2021)
Human	RPE	hfRPE	rhTGF-β2	↑*LDHA, PKM2, PFKFBP3* ↓*PGC-1α* ↑Maximal ECAR	NA	Shu et al. (2021)
Human	RPE	Primary RPE cells	TGF-β2	↑Glucose consumption	NA	Hansman et al. (2024)
Human	Stomach	MKN45 and MGC803 gastric cancer cells	TGF-β	↑*CPT1A,* ↑FAO ↑ATP	NA	Li et al. (2023)
Human, mouse	Fibroblasts	AKR-2B, Swiss 3T3, IMR90, MRC5, TIG-1, NHLF	TGF-β	↑*HK2* ↑HK2, HK2 activity ↓HK1 ↑Lactate production ↑Basal and maximal OCR	Smad2/3, PI3K, ERK, PDGFR, EGFR	Yin et al. (2019)
Human, mouse	Fibroblasts	Primary human fibroblasts, AKR-2B, MRC5	TGF-β	↑GLS1 ↑Glutamate production	SMAD2/3, PI3K/mTORC2/PDGFR, SIRT7, FOXO4	Choudhury et al. (2020)
Mink	Lung	Mv1Lu lung epithelial cells	TGF-β1	↓Basal OCR ↓Mitochondrial membrane potential ↓Complex IV activity	ROS	Yoon et al. (2005)
Mouse	Adipose	3T3-L1 adipocytes	TGF-β	↓PPARγ	NA	Xing et al. (1997)
Mouse	Bone marrow	Primary dendritic cells	TGF-β2	↑Lipid droplet formation	NA	Trempolec et al. (2020)
Mouse	Fibroblast	NIH-3T3	TGF-β1	↑Glucose consumption ↑P5cs, Gls1 ↑Glutaminolysis ↑TCA metabolites and proline ↑Basal and maximal OCR	NA	Schwörer et al. (2020)
Mouse	Fibroblast	Quiescent Swiss 3T3	Human platelet TGF-β1	↑Slc2a1, ↑Glucose consumption	NA	Kitagawa et al. (1991)
Mouse	Immune system	CD8^+^ T-cells	rhTGF-β1	↑Maximal OCR ↑Mitochondrial mass ↑Mitochondrial membrane potential	mTORC1	Gabriel et al. (2021)
Mouse	Kidney	Primary and AI podocytes	rhTGF-β1	↑Basal and maximal ECAR ↑Basal and maximal OCR ↓*Pgc-1α* ↑FAO ↑ATP	mTOR	Abe et al. (2013)
Mouse	Immune cells	RAW 264.7 macrophages	rmTGF-β	↑Lipid droplet formation	NA	Bose et al. (2019)
Mouse	Breast	NMuMG, EpH4	rmTGF-β1	↓Glut1 ↓Glucose uptake	NA	Nilchian et al. (2020)
Mouse	Spleen	Primary dendritic cells	rhTGF-β	↑*Ido1*, Arg*1*	NA	Mondanelli et al. (2017)
Mouse	Thymus	Treg cells	rhTGF-β	↓GLUT1, GLUT3, *Hk2*, ↓Basal and maximal ECAR	PI3K/mTOR	Priyadharshini et al. (2018)
Mouse	Thymus	Treg cells	TGF-β1	↓Lactate production ↓*Slc2a1, Hk2, Eno1, Hif1a* ↓Hk2, Glut1	NA	Chen et al. (2020)
Rat	Heart	Primary vascular smooth muscle cells	TGF-β1	↑Arginine uptake ↑Arginase and ornithine aminotransferase activity ↑Proline synthesis	NA	Durante et al. (2001)
Rat	Kidney	NRK-49F fibroblasts	TGF-β	↑AIB uptake ↑Lactate production	NA	Boerner et al. (1985)
Rat	Kidney	Primary mesangial	Human platelet TGF-β1	↑Slc2a1 ↑Glucose consumption	NA	Inoki (1999)
Rat	Peritoneum	Primary macrophages	TGF-β1	↑Arginase activity	NA	Boutard et al. (1995)
Rat	RPE/choroid	Eyecups	TGF-β2	↑Glucose consumption	NA	Hansman et al. (2024)

1. rhTGF-β = recombinant human TGF-β, rmTGF-β = recombinant mouse TGF-β.

2. ↑ indicates and increase, ↓ indicates a decrease.

**Table 3:** The effects of IL-6 on metabolism in different experimental systems.

Organism	Tissue	Cell line/model	Treatment^1^	Effect on metabolism^2^	Signalling pathway	Reference
Human	Adipose	Adipose tissue explants	rhIL-6	↑Lipolysis	NA	Trujillo et al. (2004)
Human	Adipose	Primary adipocytes	rhIL-6	↑Lipolysis ↓Glycerol 3-phosphate dehydrogenase activity	NA	Päth et al. (2001)
Human	Colon	SW480 colorectal adenocarcinoma cells	rhIL-6	↑*SLC2A1, HK2, PKM2, PFKFB3, LDHA* ↑Glucose consumption, lactate production	NA	Han et al. (2016)
Human	Immune system	THP-1 monocytes	rhIL-6	↑*SLC2A1, SLC2A3* ↑HK activity ↑Glucose carbon labelling of glycolytic and TCA intermediates ↑Basal and maximal ECAR ↑Succinate, pyruvate, lactate ↑Maximal OCR ↑Complex I activity	IL-6 classic and trans-signalling, JAK/STAT3, PDK, SIRT2	Xu et al. (2024)
Human	Liver	HepG2 hepatoma cells	IL-6	↓*PPARα, C/EBPα* ↑C/EBPβ, C/EBPδ	NA	Chew et al. (2007)
Human	Liver	HepG2 hepatoma cells		↓ApoA1, ApoB, LCAT ↓TG, cholesterol	NA	Ettinger et al. (1994)
Human	Plasma	NA	IL-6 infusion	↑Oxygen consumption ↑FFA ↑Adipose FFA and glycerol release	NA	Lyngsø et al. (2002)
Human	Plasma	NA	rhIL-6 infusion	↑Plasma FFA ↑Systemic FA turnover	NA	Petersen et al. (2005)
Human	Retina	Primary retinal endothelial cells	IL-6 + sIL-6R	↓Max OCR ↑Glycolytic ATP production	NA	Hoffman et al. (2023)
Human	RPE	ARPE-19 cells	IL-6	↑Lactate production	NA	Hansman et al. (2024)
Human	Skeletal muscle	Muscle tissue explants	rhIL-6	↑Glucose consumption ↑Glycogen synthesis	NA	Glund et al. (2007)
Human	Skeletal muscle	Primary cells	rhIL-6	↑*SLC2A1, PGC-1α* ↑Glucose consumption, lactate production ↑Glycogen synthesis ↑Fatty acid uptake ↑FAO	PI3K/AKT, AMPK	Al-Khalili et al. (2006)
Human	Skeletal muscle	Primary myotubes	rhIL-6	↑Insulin-stimulated glycogen synthesis	NA	Weigert et al. (2005)
Human	Whole body	NA	rhIL-6 infusion	↑Glucose utilisation ↑Oxygen consumption	NA	Carey et al. (2006)
Human	Whole body	NA	rhIL-6 infusion	↑Systemic oxygen consumption ↑Plasma lactate, ketones, FFA ↑Hepatic glucose release	NA	Stouthard et al. (1995)
Human	Whole body	NA	rhIL-6 infusion	↑Plasma FFA ↑Plasma TAG ↑Systemic lipolysis	NA	Van Hall et al. (2003)
Human	Whole body	NA	rhIL-6 infusion	↑Whole body FAO	NA	Wolsk et al. (2010)
Mouse	Adipocytes	3T3-L1	IL-6	↓*Slc2a4* ↓Insulin-stimulated glucose uptake ↑Insulin resistance	NA	Rotter et al. (2003)
Mouse	Adipocytes	3T3-L1	rmIL-6	↑Glucose uptake	NA	Stouthard et al. (1996)
Mouse	Adipocytes	3T3-L1	IL-6	↑Glucose uptake ↑*Pgc-1α* ↓Mitochondrial membrane potential ↑Mitochondrial structural abnormalities ↑Mitochondria count ↓ATP ↑Lipolysis	NA	Ji et al. (2011)
Mouse	Adipose	3T3-F442A pre-adipocytes	rhIL-6	↓Glut4 ↓*Fasn, Gapdh* ↓Insulin-stimulated glucose uptake ↓Insulin-stimulated lipogenesis	NA	Lagathu et al. (2003)
Mouse	Adipose	3T3-L1	rhIL-6	↑FAO ↑Lipolysis	NA	Petersen et al. (2005)
Mouse	Adipose	3T3-L1	rmIL-6	↑Glucose uptake	NA	Carey et al. (2006)
Mouse	Immune system	RAW 264.7 macrophages	mIL-6	↑*Hk2, Pkm2, Pfkfb3* ↑Glucose consumption, lactate production ↑ATP	PI3K/AKT	Kumari et al. (2020)
Mouse	Liver	C57BL/6	rhIL-6 infusion	↓Insulin sensitivity	NA	Klover et al. (2003)
Mouse	Liver	Primary hepatocytes	rhIL-6	↑Glycogen synthesis ↓Insulin-stimulated glycogen synthesis	NA	Senn et al. (2002)
Mouse	NA	Il6^−/−^ C57BL/6	NA	↑Obesity ↓Glucose tolerance	NA	Wallenius et al. (2002)
Mouse	NA	Il6^−/−^ C57BL/6	NA	↓Insulin sensitivity ↓Complex II, III, and IV when fed HFD	NA	Matthews et al. (2010)
Mouse	Plasma, liver, adipose, skeletal muscle	Obese C57BL/6	Injection of *IL-6* expression plasmid	↑Insulin sensitivity ↑Glucose tolerance ↓Hepatic *Pparγ, Cd36, Fabp4, Fasn* ↓Hepatic TG, FFA, and cholesterol ↑BAT *Cd36, Hsl, Atgl, Cpt1a, Cpt1b, Ucp1, Ucp2, Pgc-1α, Pgc-1β* ↑WAT *Hsl, Atgl, Cpt1a, Cpt1b, Ucp2, Ucp3, Pgc-1α* ↑Skeletal muscle AMPK and ACC phosphorylation	NA	Ma et al. (2015)
Mouse	Skeletal muscle	C2C12 myotubes	rhIL-6	↑Basal and maximal OCR ↑Complex I, II, III, and V expression ↑PGC-1α and PGC-1β	JAK/STAT3	Abid and Ryan (2020)
Mouse	Skeletal muscle	C57BL/6	Intraperitoneal rmIL-6 injection	↑AMPK phosphorylation ↑ACC phosphorylation ↑PDHa activity	NA	Biensø et al. (2014)
Mouse	Skeletal muscle	C57BL/6	Intramuscular electroporation of IL-6 expression plasmid	↓COX activity ↓Complex I and II	NA	Vanderveen et al. (2019)
Mouse	Skeletal muscle, liver	C57BL/6	rmIL-6 infusion	↓Insulin sensitivity	NA	Kim et al. (2004)
Rat	Liver	Isolated perfused liver	rhIL-6 infusion	↓Glucose release	NA	Dent et al. (2016)
Rat	Liver	Primary hepatocytes	rhIL-6	↓Glycogen synthesis ↓Glycogen synthase activity	NA	Kanemaki et al. (1998)
Rat	Liver	Primary hepatocytes	rhIL-6	↑Lactate production ↓ATP	NO	Kitade et al. (1996)
Rat	Liver	Primary hepatocytes	rhIL-6	↓FAO	NA	Nachiappan et al. (1994)
Rat	Pancreas	Primary pancreatic islet cells	rhIL-6	↓ATP ↑Glucose oxidation and OCR in high glucose conditions	NA	Sandler et al. (1990)
Rat	Skeletal muscle	L6 myotubes	rmIL-6	↑GLUT4 plasma membrane translocation ↑Glucose uptake ↑AMPK phosphorylation ↑ACC phosphorylation ↑FAO	PI3K/AKT, AMPK	Carey et al. (2006)
Rat	Skeletal muscle	Skeletal muscle explants	IL-6	↑AMPK phosphorylation	NA	Kelly et al. (2004)
Rat	Skeletal muscle	Skeletal muscle explants	rrIL-6	↑FAO	NA	Bruce and Dyck (2004)

1. rhIL-6 = recombinant human IL-6, rmIL-6 = recombinant mouse IL-6, mIL-6 = mouse IL-6.

2. ↑ indicates and increase, ↓ indicates a decrease.

**Table 4:** The effects of IL-1β on metabolism in different experimental systems.

Organism	Tissue	Cell line/model	Treatment^1^	Effect on metabolism^2^	Signalling pathway	Reference
Human	Adipose	Primary adipocytes	IL-1β	↓*SLC2A4, GSK-3β, C/EBPα* ↑Lipolysis ↑Insulin resistance	NA	Gao et al. (2014)
Human	Bone marrow	Primary bone marrow adipocytes	rhIL-1β	↑Lipolysis ↓TG synthesis	NA	Delikat et al. (1995)
Human	Cartilage	Primary articular chondrocytes	IL-1β	↑Glucose consumption and lactate production	NA	Lee et al. (2002)
Human	Cartilage	Primary chondrocytes	IL-1β	↓Complex I activity ↓Mitochondrial membrane potential ↓ATP	NA	López-Armada et al. (2006)
Human	Cartilage	Primary OA chondrocytes	hIL-1β	↓Basal and maximal OCR ↓ATP	NA	Eitner et al. (2021)
Human	Immune cells	iTreg cells	rhIL-1β	↑HIF-1α ↑*SLC2A1* ↓Maximal OCR	mTOR	Feldhoff et al. (2017)
Human	Skin	GM 8333 A skin fibroblasts	rhIL-1β	↑Total cholesterol, cholesterol ester, phosphatidylcholine, sphingomyelin	NA	Kronqvist et al. (1999)
Human	Spine	Nucleus pulposus cells	IL-1β	↓ATP production ↓Mitochondrial membrane potential	NA	Shen et al. (2017)
Human	Whole body	Type 2 diabetes patients	IL-1Ra	↓Fasting blood glucose	NA	Larsen et al. (2007)
Mouse	Adipose	3T3-F442A and 3T3-L1 adipocytes	rmIL-1β	↓Insulin-stimulated glucose uptake and utilisation ↓*Slc2a4, Fasn, Acac, Fabp4, Lpl* ↓Pparγ, C/ebpα ↑Lipolysis	NA	Lagathu et al. (2006)
Mouse	Adipose	3T3-L1 adipocytes	rmIL-1β	↑GLUT1 ↑GLUT1 plasma membrane trafficking ↑Glucose uptake ↑Insulin resistance	NA	Jager et al. (2007)
Mouse	Adipose	3T3-L1 adipocytes	IL-1β	↑Insulin resistance	NA	Handa et al. (2013)
Mouse	Adipose	3T3-L1 adipocytes	IL-1	↓LPL activity	NA	Beutler and Cerami (1985)
Mouse	Adipose	3T3-L1 adipocytes	rIL-1	↓LPL activity ↑Lipolysis	NA	Price et al. (1986a)
Mouse	Adipose	Primary adipocytes	IL-1β	↑Basal and maximal ECAR ↓Basal and maximal OCR ↓Complex I and III activity ↓FAO ↓ATP	IRAK2	Zhou et al. (2020)
Mouse	Adipose	Primary preadipocytes	hIL-1β	↓LPL activity ↓GPDH activity	NA	Grégoire et al. (1992)
Mouse	Immune system	J774 macrophages	rIL-1β	↓*Abca1* and *Abcg1*	NA	Khovidhunkit et al. (2003)
Mouse	Liver	C57BI/6	Intraperitoneal rhIL-1β injection	↑FAS ↑Cholesterol synthesis	NA	Feingold et al. (1989b)
Mouse	Liver	C57BI/6	Intramuscular rhIL-1β injection	↑Hepatic FAS ↑Hepatic citrate	NA	Grunfeld et al. (1990)
Mouse	Liver	FL83B hepatocytes	rmIL-1β	↑Insulin resistance	NA	Wen et al. (2011)
Mouse	Liver	Primary hepatocytes	rmIL-1β	↑FAS ↑TG	NA	Negrin et al. (2014)
Mouse	Liver	Primary hepatocytes	IL-1β	↑TG	NA	Miura et al. (2010)
Mouse	Serum, adipose, skeletal muscle, heart	C57BI/6	Intraperitoneal rhIL-1β injection	↑Serum TG ↓Adipose, skeletal muscle and heart LPL activity	NA	Feingold et al. (1994)
Mouse	Serum, liver	C57BI/6	Intraperitoneal rhIL-1β injection	↑Serum TG and cholesterol ↑Hepatic HMG-CoA reductase activity	NA	Memon et al. (1993)
Mouse	Trachea	Primary tracheal epithelial cells	IL-1β	↑*Slc2a1, Slc2a3, Slc16a3, Pfkl, Hk2, Pkm2, Ldha* ↑Glucose uptake and lactate production ↑Basal and maximal ECAR	NA	Qian et al. (2018)
Mouse	Whole body	C57BL/6	IL-1β-secreting tumour inoculation	↓Serum glucose ↑Peripheral tissue glucose uptake ↑Hepatic *Slc2a3* ↓Hepatic *Slca2* ↓Hepatic gluconeogenesis ↓Hepatic glycogen	NA	Metzger et al. (2004)
Mouse	Whole body	C57BL/6	rmIL-1β	↑Insulin resistance	NA	Wen et al. (2011)
Mouse	Whole body	C57BL/6 IL-1RI^−/−^	NA	↑Insulin sensitivity ↑Glucose tolerance	NA	McGillicuddy et al. (2011)
Mouse	Whole body	C57BL/6 IL-1β^−/−^	NA	↑Insulin sensitivity	NA	Stienstra et al. (2010)
Mouse	Whole body	Mature C57BL/6 IL-1RI^−/−^	NA	↓Glucose tolerance ↓Insulin sensitivity ↑Obesity	NA	García et al. (2006)
Rat	Heart	Primary cardiomyocytes	rIL-1β	↑Glucose consumption and lactate production ↓Complex I and II activity ↓ATP	NO	Tatsumi et al. (2000)
Rat	Liver	Primary hepatocytes	rhIL-1β	↑Lactate production ↓ATP, ketogenesis	NO	Kitade et al. (1996)
Rat	Liver	Primary hepatocytes	rhIL-1β	↓Basal OCR	NA	Berthiaume et al. (2003)
Rat	Ovary	Primary ovarian cells	rhIL-1β	↑Glut1, Glut3 ↑Glucose consumption	NO	Kol et al. (1997)
Rat	Ovary	Primary ovarian cells	rhIL-1β	↑Glucose consumption and lactate production	NA	Ben-Shlomo et al. (1997)
Rat	Pancreas	INS-1 insulinoma	IL-1β	↓Mitochondrial membrane potential	NA	Veluthakal et al. (2005)
Rat	Pancreas	Primary pancreatic islet cells	IL-1β	↓Glucose utilisation	NO	Ma et al. (1997)
Rat	Retina	r28 and RGC-5 retinal neurons	IL-1β	↑Glucose consumption and lactate production ↓Cellular reductive potential ↓Mitochondrial membrane potential ↓Basal OCR ↓ATP	NA	Abcouwer et al. (2008)
Rat	Testes	Primary Sertoli cells	rrIL-1β	↑*Ldha* ↑Glucose consumption and lactate production	PI3K/AKT, MEK/ERK	Riera et al. (2007)
Rat	Whole body	GK type 2 diabetic rats	IL-1Ra	↓Hyperglycaemia ↑Insulin sensitivity	NA	Ehses et al. (2009)

1. rhIL-1β = recombinant human IL-1β, hIL-1β = human IL-1β, rmIL-1β = recombinant mouse IL-1β, rIL-1β = recombinant IL-1β.

2. ↑ indicates and increase, ↓ indicates a decrease.

**Table 5:** The effects of oxidative stress on metabolism in different experimental systems.

Organism	Tissue	Cell line/model	Treatment	Effect on metabolism^a^	Signalling pathway	Reference
Fish (Yellow Catfish)	Liver	Primary hepatocytes	NAC	Prevents ↑FAS and 6PGD activity, ↑TG content due to high glucose	NA	Zhao et al. (2020)
Human	Blood	Primary erythrocytes	H_2_O_2_	↑AMP and IMP ↓ATP and GTP	NA	Tavazzi et al. (2000)
Human	Bone marrow	B1647 leukemia	NOX inhibitors, antioxidants	↓Glucose uptake	NA	Prata et al. (2008)
Human	Breast	MDA-MB-157 and MCF-7	H_2_O_2_	↑*FASN*	NA	Furuta et al. (2008)
Human	Kidney	HEK 293	H_2_O_2_	↑AMPK and ACC phosphorylation ↑AMPK activation	AMPK	Zmijewski et al. (2010)
Human	Liver	HepG2 hepatoma cells	H_2_O_2_	↑*FAS, ACC, SREBP1c, SCD1* ↑TG content	NA	Sekiya et al. (2008)
Human	Liver	HL7702 hepatocytes	H_2_O_2_	↓*PPARα, CPT1*	NA	Li et al. (2012)
Human	Liver	SMMC-7721 hepatoma cells	Xanthine oxidase (XO)	↑HK2, HIF-1α ↑LDH activity	HIF-1	Shi et al. (2009)
Human	Umbilical cord	HUVEC	H_2_O_2_	↓PPARγ expression and activity	NA	Blanquicett et al. (2010)
Mouse	Adipose	3T3-L1	GO	↑Glucose uptake ↑*Slc2a1* ↓*Slc2a4* ↑Glut1 ↓Glut4 ↓Glycogen synthase activity ↑Lipogenesis ↑Insulin resistance	NA	Rudich et al. (1997)
Mouse	Adipose	3T3-L1	TBHP, NAC	↓*Scd1* and *Acly* (with TBHP treatment) *↑Scd1* and *Acly* (with NAC treatment)	NA	Okuno et al. (2018)
Mouse	Adipose	3T3-L1 cells	H_2_O_2_	↑Fas, Acc, Srebp1, ↑TG content ↓Lipolysis	NA	Sun et al. (2020)
Mouse	Adipose	C57BL/6 Nrf2^−/−^ mouse primary adipocytes	NA	↑Basal OCR and maximal OCR	NA	Sun et al. (2020)
Mouse	Fibroblasts	NIH 3T3	H_2_O_2_	↑AMPK phosphorylation ↑AMPK activity ↑AMP/ATP ratio	AMPK	Choi et al. (2001)
Mouse	Fibroblasts, neurons	Nrf2^−/−^ MEFs, cortical neurons	NA	↓Mitochondrial membrane potential ↓Basal OCR ↓Mitochondrial NADH pool ↓Cortical neuron FADH2 regeneration ↓Cortical neuron ATP	NA	Holmström et al. (2013)
Mouse	Heart	Nrf2^−/−^ MEFs, heart slices	NA	↓MEF basal and maximal OCR ↓Heart FADH2 regeneration	NA	Ludtmann et al. (2014)
Mouse	Lens	αTN4-1 lens epithelial cells	H_2_O_2_	↓GAPDH activity ↓NAD ↓Mitochondrial viability ↓ATP	PARP	Spector et al. (2002)
Mouse	Liver	C57BL/6 Nrf2^−/−^ mice	NA	↓Hepatic NADH	NA	(Y. K. J. Zhang et al., 2012a)
Mouse	Liver	C57BL/6 Nrf2^−/−^ mice	NA	↓*Pparγ, Srebp1, Srebp2, Scd1, Acc* ↓Hepatic lipid and TG levels	NA	Huang et al. (2010)
Mouse	Liver	C57BL/6 Nrf2^−/−^ mice	NA	↑*Pparα, Cpt1a*	NA	Tanaka et al. (2012)
Mouse	Liver	C57BL/6 Nrf2^−/−^ mice	NA	↓*Pparγ*	NA	Tanaka et al. (2008)
Mouse	Liver	C57BL/6 Nrf2^−/−^ mice	NA	↓*Fasn*	NA	Zhang et al. (2010)
Mouse	Liver	C57BL/6J, liver-targeted CKO *Alb-CreKeap1^flox/−^* mice	CDDO-Im (Nrf2 activator)	↓Hepatic *Srebf1, fasn, Acaca, Acly* ↑Hepatic *Slc2a1, Cd36, Abcd1*	NA	Yates et al. (2009)
Mouse	Liver, adipose	ROS-augmented (adipose glutathione depleted) mice	NA	↑Hepatic TG content ↓Gonadal WAT *Acly, Scd1, Fasn, Acaca, Srebf1* ↓BAT *Ucp1, Ppargc1a*	NA	Okuno et al. (2018)
Mouse	Lung	Lewis lung carcinoma cells	H_2_O_2_, Resveratrol, NAC	↑Glucose uptake ↓Glut1, glucose uptake and lactate production with antioxidant treatment ↑OCR with antioxidant treatment	HIF-1α, PI3K/AKT, mTOR	Jung et al. (2013)
Mouse	Lymphocytes	P388D monocytes	H_2_O_2_	↓Glucose uptake and lactate production ↓GAPDH activity, ↑PPP flux ↓NAD^+^ and NADH ↓OCR ↓ATP	NA	Hyslop et al. (1988)
Mouse	Muscle	C2C12 myotubes	H_2_O_2_	↓Citrate synthase activity ↓*Pgc-1α* ↓mtDNA copy number	NA	Bonnard et al. (2008)
Mouse	RPE	*VMD2-Cre;Sod2^flox/flox^* BALB/cJ mice	RPE *Sod2* conditional knockout	↑Glycolytic metabolism ↑*Pfk1, Hk, Pkm1, Pkm2, Slc2a1* ↑Mitochondrial structural abnormalities ↓Complex III ↓ATP	NA	Brown et al. (2019)
Rat	Adipose	Primary adipocytes	H_2_O_2_	↑Glucose uptake	NA	Hayes and Lockwood (1987)
Rat	Heart	Primary cardiomyocytes	H_2_O_2_	↓GAPDH and PDH activity ↓Glucose uptake, lactate production ↓Glucose oxidation ↓ATP	NA	Janero et al. (1994)
Rat	Muscle	L6 myoblasts	GO, XO	↑*Slc2a1* expression and stability	NA	Kozlovsky et al. (1997)
Rat	Retina	TR-iBB retinal endothelial cells	GO	↓*Slc2a1* ↓Plasma membrane Glut1 ↓Glucose consumption	PI3K/AKT	Fernandes et al. (2011)
Rat	Skeletal muscle	Primary skeletal muscle cells	H_2_O_2_, NAC	↑*Gapdh, Pfk, Hk2, Slc2a4* ↑Glucose uptake and lactate production ↑HK and G6PD activity	NA	Pinheiro et al. (2010)
Rat	Skeletal muscle	Skeletal muscle explants	H_2_O_2_	↑Glucose uptake ↑Insulin resistance	IR, p38 MAPK	Archuleta et al. (2009)
Rat	Skeletal muscle	Skeletal muscle explants	H_2_O_2_	↑Glucose uptake ↑Glycogen synthase activity ↑AMPK phosphorylation	PI3K, p38 MAPK	Kim et al. (2006)
Rat	Skeletal muscle	Skeletal muscle explants	XO, H_2_O_2_	↑Glucose uptake ↑AMPKα1 activity	PI3K	Higaki et al. (2008)
Rat	Skeletal muscle	Skeletal muscle explants	H_2_O_2_	↑Glucose uptake ↑AMPK phosphorylation ↑ACC phosphorylation ↑AMPKα1 activity ↓ATP	NA	Toyoda et al. (2004)
Rat	Skeletal muscle	Skeletal muscle explants	H_2_O_2_	↑Glucose uptake ↑AMPKα1 and AMPKα2 activity ↑ACC phosphorylation	AMPK	Jensen et al. (2008)
Rat	Skeletal muscle	Skeletal muscle explants	H_2_O_2_	↑Glucose uptake ↑Glycogen synthase activity ↑Glycogen synthesis ↑GSK-3β phosphorylation ↑Insulin resistance	GSK-3β	Dokken et al. (2008)
Rat	Various tissues	Clone 9 hepatocytes, NIH-3T3 fibroblasts, 3T3-L1 adipocytes, C2C12 myoblasts	H_2_O_2_	↑Glucose uptake	PLC	Prasad and Ismail-Beigi (1999)

a ↑ indicates and increase, ↓ indicates a decrease.

**Table 6:** The effects of EGF on metabolism in different experimental systems.

Organism	Tissue	Cell line/model	Treatment^1^	Effect on metabolism^2^	Signalling pathway	Reference
Chicken	Liver	Chick embryonic hepatocytes	EGF	↑AIB uptake	PKC	Marino et al. (2000)
Goat	Breast	Primary mammary epithelial cells	EGFR overexpression, EGF treatment	↑*Fasn, Acc, Scd1, Srebf1* ↑Intracellular TG content	PLCγ1/AKT	(J. Huang et al., 2020a)
Hamster	Sebaceous glands	Primary sebocytes	rhEGF	↓Lipid content ↓TG, FFA, cholesterol content	NA	Sato et al. (2001)
Human	Brain	LN229 and U251 glioblastoma cells	hEGF	↑GDH1 ↑Glutaminolysis ↑Glutamine carbons incorporation into TCA cycle	MEK/ERK/ELK1	Yang et al. (2020)
Human	Brain	U251 glioblastoma cells	EGF	↑GLUT1 ↑PFK1 and PFK2 activity	PFKP, PI3K/AKT	Lee et al. (2018)
Human	Brain	U87 and U87-EGFRvIII glioblastoma cells	EGF	↑Fatty acid content ↑SREBP1 nuclear translocation ↑FAS and ACC	NA	Guo et al. (2009b)
Human	Brain	U87/EGFR (EGFR overexpressing) glioblastoma cells	EGF	↑Basal ECAR ↓Glucose oxidation ↓Basal OCR	PGK1, PDK1	Li et al. (2016)
Human	Brain	U87-EGFRvIII glioblastoma cells	EGFRvIII mutant	↑Glucose consumption ↑Fatty acid content ↑AMPK and ACC phosphorylation	AMPK, mTOR	Guo et al. (2009a)
Human	Brain	U87MG glioblastoma cells	EGFR overexpression	↑Glutamate export ↑Cysteine uptake	EGFR interaction with system xc(−)	Tsuchihashi et al. (2016)
Human	Breast	HS578T breast ductal carcinoma cells	EGF	↑Glucosome formation	MEK/ERK	Jeon et al. (2022)
Human	Breast	MDA-MB-231 breast epithelial carcinoma cells	EGFR overexpression	↑Glucose uptake and lactate production ↑c-Myc ↓TXNIP	NA	Iqbal et al. (2021)
Human	Breast	MDA-MB-231 breast epithelial carcinoma cells	EGF	↑Glucose consumption	NA	Kaplan et al. (1990)
Human	Breast	MDA-MB-468 breast epithelial carcinoma cells	EGF	↑*HK2* ↑*HK2* activity ↑PKM2 phosphorylation ↓PKM2 activity ↑Lactate production ↑Basal and maximal ECAR	EGFR	Lim et al. (2016)
Human	Breast	T-47D breast ductal carcinoma cells	EGF	↑Glucose uptake and lactate production ↑HK2 activity ↑GLUT1 plasma membrane localisation	PI3K/AKT/mTOR	Jung et al. (2019)
Human	Cervix	HeLa cervical cancer cells	EGF	↑GLUT4 localisation to plasma membrane	NA	Trefely et al. (2015)
Human	Colon	C2BBe1 colorectal adenocarcinoma cells	rhEGF	↑Intracellular triglyceride and cholesterol content	NA	Liang et al. (2002)
Human	Colon	C2BBe1 colorectal adenocarcinoma cells	EGF	↑Glutamine uptake	NA	Ray et al. (2005)
Human	Colon	Caco-2 colorectal adenocarcinoma cells	EGF	↓I-FABP ↓DGAT activity ↓Fatty acid uptake ↑Phospholipid biosynthesis ↓TG biosynthesis	NA	Darimont et al. (1999)
Human	Colon	DLD-1 and HCT 116 colorectal adenocarcinoma cells	EGF	↑FASN	CSN6	Wei et al. (2023)
Human	Colon	DLD1 colorectal cancer cells	EGFR inhibition	↓Serine/glycine biosynthesis	MEK/ERK/ILF3	(K. Li et al., 2020c)
Human	Colon	HT29, HTC116, SW480, SW620 colorectal adenocarcinoma cells	EGF	↑Lipid droplet density	PI3k/mTOR	Penrose et al. (2016)
Human	Head and neck	HN5 squamous cell carcinoma cells	EGFR inhibitor	↓Glutamine uptake	EGFR association with ASCT2	Lu et al. (2016)
Human	Liver	HepG2 hepatoma cells	EGF	↑ApoA1	EGFR, Ras, MEK/ERK, Sp1	Zheng et al. (2001)
Human	Lung	Beas-2B bronchial epithelial cells, PC9, HCC927, and H1975 NSCLC cells	EGFR	↑LDLR, SREBP1	AKT	Luo et al. (2021)
Human	Lung	CL1-0, CL1-5, and H1299 NSCLC cells	EGF	↑ATP ↑Mitochondrial fission ↓Mitochondrial fusion	EGFR translocation to mitochondria	Che et al. (2015)
Human	Lung	HCC827 and PC9 NSCLC cells	EGFR inhibition	↓Glucose consumption and lactate production ↓Basal ECAR ↓Glycolytic and PPP intermediates ↑Aspartate ↓*HK2, SLC2A1, c-Myc* ↓HCC827 basal OCR	EGFR	Makinoshima et al. (2014)
Human	Lung	HCC827 NSCLC cells	EGFR inhibition	↓HK2, GLUT1, c-Myc, HIF-1α, ASCT2 ↓Glucose consumption and lactate production ↓Basal OCR ↑AMPK phosphorylation	EGFR	Momcilovic et al. (2017)
Human	Lung	HCC827 NSCLC cells	EGFR inhibition	↓SCD1 ↓MUFA synthesis	EGFR	(J. Zhang et al., 2017b)
Human	Lung	HCC827, H1975, PC9 NSCLC cells	EGFR inhibition	↓SREBP1, FASN, ACC ↓TAG, DAG, PE	GSK3/FBXW7	Chen et al. (2021)
Human	Lung	PC9 NSCLC cells	EGFR inhibition	↓SREBP1, FASN, SCD1 ↓Lipogenesis	SREBP1	Xu et al. (2021)
Human	Lung	PC9 NSCLC cells	EGFR inhibition	↓SREBP1 and FASN	NA	Ali et al. (2018)
Human	Lung	PE089 and Ire NSCLC cells	EGF	↑Basal and maximal OCR ↑ATP	EGFR translocation to mitochondria	(C.-Y. Huang et al., 2020b)
Human	Pancreas	HPAC and AsPC-1 pancreatic ductal adenocarcinoma cells	EGF	↑*FASN*	EGFR, MEK/ERK	Bian et al. (2015)
Human	Placenta	Primary placental cells	EGF	↑AIB and glucose uptake	NA	Guyda (1991)
Human	Plasma	NA	NA	Plasma ApoA1 and HDL-cholesterol correlates with EGF	NA	Berrahmoune et al. (2009)
Human	Prostate	LNCaP and PC-3 adenocarcinoma cells	EGF	↑Leucine uptake ↑LAT3 plasma membrane expression	PI3K/AKT	Zhang et al. (2019)
Human	RPE	Primary RPE cells	EGF	↑Glucose consumption	NA	Takagi et al. (1994a)
Human	Sebaceous glands	SZ95 sebocytes	EGFR inhibitor	↑TG, cholesterol ester ↑*SREBF1, FASN, SCD1*	NA	Dahlhoff et al. (2015)
Human	Skin	A431 squamous cell carcinoma cells	EGF	↑PFK-2 activity ↑Fructose-2,6-bisphosphate abundance	NA	Baulida et al. (1992)
Human	Skin	HaCaT keratinocytes	EGFR inhibitor, rhEGF	↓*FASN, ACC* with EGF inhibitor ↑FASN, ACC, HMG-CoA reductase wit rhEGF	NA	Oh et al. (2022)
Mouse	Adipose	3T3-L1	EGF	↑Glycogen synthesis ↑Lipogenesis	NA	Van Epps-Fung et al. (1996)
Mouse	Fibroblasts	10T1/2	EGFR	↓Cox activity ↓ATP	EGFR translocation to mitochondria	Demory et al. (2009)
Mouse	Fibroblasts	NIH 3T3 DHER1	EGF	↑Glucose uptake ↑AIB uptake	NA	Scimeca et al. (1989)
Mouse	Fibroblasts	Swiss 3T3	EGF	↑Lactate production	NA	Diamond et al. (1978)
Mouse	Immune system	BAF3-EGFR pro-B-cells	Constitutively active EGFR	↑*SLC2A1, HK2, PFKL, LDHA* ↑Lactate production ↑Glucose carbon incorporation into TCA cycle metabolites ↑Serine-glycine biosynthesis pathway genes ↓Glutaminolysis	cMyc, ATF4	Jin et al. (2019)
Mouse	Plasma, liver	129S1/SvImJ-*Egfr^Dsk5^* mice (hyperactive EGFR mutant)	NA	↑Plasma cholesterol, TG, and LDL ↑Hepatic cholesterol ester ↓Hepatic TG ↑Hepatic Fas, HMG-CoA reductase	NA	Scheving et al. (2014)
Mouse, human	Fibroblasts	3T3, primary human fibroblasts	EGF	↑Glucose uptake	NA	Barnes and Colowick (1976)
Pig	Testes	Primary Sertoli cells	EGF	↑*Ldha*	PKC	Boussouar and Benahmen (1999)
Rabbit	Kidney	Primary renal proximal tubules	EGF	↑Glucose consumption and lactate production ↑G6PD activity ↓Fructose 1,6-bisphosphatase activity ↓Basal OCR	NA	Nowak and Schnellmann (1995)
Rat	Adipose	Primary adipocytes	EGF	↑Glucose consumption	NA	Stagsted et al. (1993)
Rat	Kidney	NRK-49F fibroblasts	EGF	↑Lactate production ↑AIB uptake	NA	Boerner et al. (1985)
Rat	Liver	Primary hepatocytes	EGF	↑Glycogen synthesis ↑Glycogen synthase activity	NA	Bosch et al. (1986)
Rat	Liver	Primary hepatocytes	mEGF	↑Glycogen synthesis	NA	Freemark (1986)
Rat	Liver	Primary hepatocytes	EGF	↑AIB uptake	NA	Bereta et al. (1990)
Rat	Serum	Pregnant Wistar ST	mEGF injection	↓Asparagine, Threonine, Proline, Valine, Leucine, Tryptophan ↑Glycine	NA	Thongsong et al. (2002)

1. rhEGF = recombinant human EGF, hEGF = human EGF, mEGF = mouse EGF.

2. ↑ indicates and increase, ↓ indicates a decrease.

**Table 7:** The effects of FGF on metabolism in different experimental systems.

Organism	Tissue	Cell line/model	Treatment^1^	Effect on metabolism^2^	Signalling pathway	Reference
Duck	Liver	Primary mule duck fatty liver cells	FGF1	↓TG content ↓*Acc1, Srebf1* ↑SPT1A, ↑AMPK expression and phosphorylation	NA	Hu et al. (2024)
Human	Breast	MCF-7, UCD12 breast epithelial carcinoma	FGF1	↑Basal ECAR ↑Basal OCR	ER	Castillo-Castrejon et al. (2023)
Human	Liver	FGFR2-fusion + intrahepatic cholangiocarcinoma	FGFR inhibition	↓*HK2, PKM2, LDHA* ↓Glucose uptake and lactate production ↓Basal ECAR ↓Glycolytic, PPP, and TCA cycle intermediates ↑FAO genes ↓FAS genes ↓Lipid droplet count ↑Lipase activity ↑FA dependency ↑Elongated mitochondria ↓Fragmented and intermediate mitochondria	NF-κB	Zhen et al. (2024)
Human	Lung	H1703, H520 squamous cell carcinoma	FGF2	↑Glucose uptake and lactate production ↓PKM2 activity ↑ATP	FGFR1, PI3K/mTOR	Fumarola et al. (2017)
Human	Prostate	DU145 prostate adenocarcinoma	FGFR1 ablation	↑*LDHB* ↓Glucose uptake and lactate production ↑OCR ↓ATP	NA	Liu et al. (2018)
Human	Prostate	PC-3 and 22Rv1 prostate adenocarcinoma	FGF1 and FGF2 inhibition	↓*LDHA/LDHB* ratio ↓Glucose consumption and lactate production ↓Basal and maximal ECAR	STAT1	Ye et al. (2024)
Mouse	Adipose	3T3-L1	FGF2	↑HIF-1α ↑GLUT1 ↑GLUT1 plasma membrane translocation ↑Glucose uptake and lactate production	HIF-1α, MEK/ERK	Kihira et al. (2011)
Mouse	Adipose	3T3-L1, mouse epididymal WAT explants	FGF1	↑Slc2a1 ↓*Slc2a4* ↑Glucose uptake	MEK/ERK, PI3K/AKT	Nies et al. (2022)
Mouse	Adipose	C57BL/J6 *Fgfr1^f/f^aP2^Cre^* (adipocyte-specific Fgfr1 ablation)	NA	↑Phospholipid biosynthesis gene expression ↑Lipogenesis gene expression ↓*Lpl, Cd36, Scd1, P6pd* ↑*Cox2, Acc1, Acly, Hk1, Hk2*	NA	Ye et al. (2016)
Mouse	Adipose	Gonadal WAT explants, SVF-derived adipocytes, 3T3-L1 adipocytes	rFGF1	↓Lipolysis	PI3K/PDE4D	Sancar et al. (2022)
Mouse	Adipose, liver	C57BL/6J Fgf2^−/−^ WAT, BAT and liver	NA	↑mtDNA copy number ↓Lipid droplet size ↑*PGC-1α*, *Ucp1, Pparγ* ↓HFD liver TG content	NA	Li et al. (2021)
Mouse	Fibroblasts	MEFs	Fgfr1/2 ablation	↑*Ldhb* ↓Isoleucine, valine, lactate, acetate ↑Glutamate, succinate ↑OCR	Tet1	Liu et al. (2018)
Mouse	Immune system	BAF3-FGFR1 Pro-B-cells	Constitutively active FGFR1	↑*Slc2a1, Hk2, Pfkl, Ldha* ↑Basal ECAR ↑Glucose-derived lactate production ↑Glucose carbon incorporation into TCA cycle metabolites ↑Serine-glycine biosynthesis pathway genes ↑Basal OCR ↓Glutaminolysis ↑ATP	c-Myc, HIF-1α	Jin et al. (2019)
Mouse	Liver	*db/db* mice	FGF1^ΔHBS^ (heparin-binding site mutant)	↓*Srebf1, Scd1, Fasn* ↑AMPK and ACC phosphorylation	NA	Lin et al. (2021)
Mouse	Liver, adipose	C57BL/J6 *Fgfr1^f/f^aP2^Cre^* (adipocyte-specific Fgfr1 ablation)	NA	↑*Liver Cd36, Pparα, Pparγ, Acc, Fasn, Scd1* *↑Liver Triglycerides* *↑Adipose lipase activity*	NA	Yang et al. (2012)
Mouse	Muscle	C2C12 myoblasts	rbFGF1	↑Mitochondrial fission ↑Mitochondrial membrane potential	ERK/DRP1	Liu et al. (2024)
Mouse	Skeletal muscle	*db/db* mice	rhFGF1 intraperitoneal injection	↑Glut4 expression and plasma membrane translocation ↑AMPK phosphorylation	AMPK	Ying et al. (2021)
Rat	Testis	Primary Sertoli cells	FGF2	↑*Slc2a1, Ldha* ↑Ldha activity ↑Glucose uptake and lactate production	NA	Riera et al. (2002)

1. rFGF1 = recombinant FGF1, rbFGF1 = recombinant bovine FGF1, rhFGF1 = recombinant human FGF1.

2. ↑ indicates and increase, ↓ indicates a decrease.

**Table 8:** The effects of IGF-1 on metabolism in different experimental systems.

Organism	Tissue	Cell line/model	Treatment^1^	Effect on metabolism^2^	Signalling pathway	Reference
Chicken	Muscle	Primary myotubes	rhIGF-1	↑AIB uptake	NA	Duclos et al. (1993)
Human	Bone marrow	RPMI 8226 multiple myeloma cells	IGF-1	↑Lactate production	NA	Freund et al. (1993)
Human	Breast	MCF-7 (with acquired resistance to IGF-1R inhibition)	IGF-1	↓Basal OCR ↑Mitochondrial mass ↑*PGC-1β*	PI3K	Lyons et al. (2017)
Human	Breast	MCF-7 breast epithelial carcinoma cells	IGF-1	↑*PGC-1β*	NA	Chang et al. (2011)
Human	Breast	MCF-7 breast epithelial carcinoma cells	IGF-1	↑AMPK and ACC phosphorylation	NA	Favre et al. (2010)
Human	Breast	MCF-7 breast epithelial carcinoma cells	IGF-1	↑HIF-1α ↑GSK-3 phosphorylation	NA	Riis et al. (2020)
Human	Breast	MCF-7, T-47D, ZR-75-1 breast cancer cells	IGF-1	↑xc(−) expression ↑Glutamate export	NA	Yang and Yee (2014)
Human	Breast	MCF-7L breast epithelial carcinoma cells	IGF-1	↑Basal and maximal ECAR	NA	Rajoria et al. (2023)
Human	Cervix	HeLa cervical cancer cells	IGF-1	↑GLUT4 plasma membrane trafficking	PFKBP3	Trefely et al. (2015)
Human	Colon	HT29 colorectal adenocarcinoma cells	Silenced IGF-1R	↑Maximal OCR	NA	(S. Q. Wang et al., 2019b)
Human	Heart	hES-derived cardiomyocytes	IGF-1	↑Basal and maximal OCR ↑Mitochondrial membrane potential ↓PDH phosphorylation	NA	Sánchez-Aguilera et al. (2023)
Human	Muscle	Primary myotubes	rhIGF-1	↑Glucose uptake	NA	Furling et al. (1999)
Human	Muscle	Primary myotubes	hIGF-1	↑Glucose uptake	NA	Henry et al. (1995)
Human	Muscle	Skeletal muscle explants	IGF-1	↑Glucose uptake	NA	Dohm et al. (1990)
Human	Placenta	BeWo choriocarcinoma cells	hIGF-1	↑AIB uptake	PI3K	Fang et al. (2006)
Human	Placenta	BeWo choriocarcinoma cells	Adenovirus-mediated hIGF-1 overexpression	↑*SNAT1, SNAT2, LAT1* ↑AIB and leucine uptake	NA	Jones et al. (2014)
Human	Placenta	BeWo choriocarcinoma cells, primary syncytial cells, placental explants	IGF-1	↑GLUT1 ↑Glucose uptake	NA	Baumann et al. (2014)
Human	Placenta	Primary trophoblasts	rIGF-1	↑AIB uptake	NA	Guyda (1991)
Human	Placenta	Primary trophoblasts	IGF-1	↑AIB uptake	NA	Karl (1995)
Human	Retina	MIO-M1 Müller cells	hIGF-1	↑GLUT1 plasma membrane trafficking ↑Glucose uptake	PI3K/AKT, MEK/ERK, LRP1	Actis Dato et al. (2021)
Human	RPE	hfRPE cells	rhIGF-1	↑Glucose uptake	NA	Takagi et al. (1994a)
Human	Skin	SEB-1 sebocytes	IGF-1	↑Lipogenesis ↑*SREBP1*	PI3K/AKT	Smith et al. (2008)
Human	Whole body	NA	rhIGF-1 infusion	↑Systemic glucose uptake ↓Hepatic gluconeogenesis ↑Plasma FFA, ketones ↓Whole-body leucine oxidation	NA	Laager et al. (1993)
Human	Whole body	NA	rhIGF-1 infusion	↑Systemic glucose uptake ↓Hepatic gluconeogenesis ↓Plasma FFA ↓Plasma Ile, Leu, Met, Phe, Thr, Val	NA	Boulware et al. (1992)
Human	Whole body	NA	rhIGF-1 infusion	↓Systemic leucine oxidation	NA	Mauras and Beaufrere (1995)
Human	Whole body, plasma	NA	rhIGF-1 infusion	↑Systemic glucose consumption ↓Plasma FFA, TG, ketones ↑Systemic leucine oxidation rate	NA	Turkalj et al. (1992)
Human	Whole body, plasma	NA	rhIGF-1 infusion	↑Systemic glucose consumption ↓Plasma urea, uric acid, creatinine ↑Plasma FFA ↑Systemic lipid oxidation ↓Systemic protein oxidation	NA	Hussain et al. (1993)
Human	Whole body, plasma	NA	rhIGF-1 infusion	↑Systemic glucose uptake ↓Plasma Val, Leu, Ile, Arg, Gly, His, Met, Phe, Tyr, Lys	NA	Elahi et al. (1993)
Human	Whole body, plasma, liver	NA	rhIGF-1 infusion	↑Systemic glucose consumption ↓Hepatic glucose production ↓Plasma FFA, ketones ↓Plasma amino acids	NA	Boulware et al. (1994)
Human, rat, mouse	Liver, muscle, adipose	HepG2 hepatoma cells, C2C12 myoblasts, 3T3-L1 adipocytes	IGF-1	↑*UCP2*	PI3K/AKT/FOXO1	Watamoto et al. (2019)
Mouse	Adipose	3T3-L1 adipocytes	IGF-1	↑GLUT4 plasma membrane trafficking ↑Glucose uptake ↑AMPK phosphorylation	PI3K	Assefa et al. (2017)
Mouse	Brain	Igfr^−/−^ mouse astrocytes	NA	↓Glucose uptake ↓Maximal ECAR ↓Basal and maximal OCR ↑Complex I activity	NA	Logan et al. (2018)
Mouse	Brain	Igfr^−/−^ mouse whole-brain synaptosomes	NA	↓Glucose uptake	NA	Cheng et al. (2000)
Mouse	Breast	Mammary tumour cells	IGF-1	↑Glucose uptake and lactate production	Irs2, PI3K	Landis and Shaw (2014)
Mouse	Fibroblasts	IGF-1R^−/−^ MEFs	IGF-1	↓Mitochondria membrane potential ↑Complex III expression ↓Complex V expression	NA	Riis et al. (2020)
Mouse	Immune system	Primary CD4^+^ T-cells	IGF-1	↑Glucose uptake ↑Basal and maximal OCR ↑ATP	NA	Kiernan et al. (2024)
Mouse	Muscle	C2C12 myotubes	IGF-1	↑mtDNA copy number, ↑*Pgc-1α*	NA	Guan et al. (2022)
Mouse	Muscle	FVB	Muscle-specific IGF-1 overexpression	↑*Pgc-1α* in aged mice	NA	Ascenzi et al. (2019)
Mouse	Serum	C57BL/6 LI-IGF-1^−/−^ (Liver specific IGF-1 knockout)	NA	↑Serum cholesterol	NA	Sjögren et al. (2001)
Rat	Adipose	Primary adipocytes	rhIGF-1	↑Lipogenesis	NA	Frick et al. (2000)
Rat	Liver	Aged Wistar rat isolated liver mitochondria	rhIGF-1	↑Mitochondria membrane potential ↑Complex V activity ↓Basal OCR	NA	Puche et al. (2008)
Rat	Muscle	L6 myotubes	rhIGF-1	↑Glut1 plasma membrane trafficking ↑Glucose uptake	NA	Bilan et al. (1992)
Rat	Muscle	Skeletal muscle explants	IGF-1	↑Glucose uptake and lactate production ↑Hexokinase activity ↑Glycogen synthesis	NA	Dimitriadis et al. (1992)
Rat	Muscle	Skeletal muscle explants	IGF-1	↑Glucose uptake ↑AIB uptake	NA	Dardevet et al. (1994)
Rat	Plasma	Wistar	rhIGF-1 injection	↓Cholesterol	NA	Pascual et al. (1995)
Rat	Spine	Primary dorsal root ganglion cells	IGF-1 expression plasmid	↑Basal and maximal ECAR ↑Maximal OCR	C/EBPβ	Aghanoori et al. (2022)
Rat	Spine	Primary dorsal root ganglion neurons	rhIGF-1	↑AMPK and ACC phosphorylation ↑Maximal OCR ↑Complex IV and V expression ↑mtDNA copy number ↑ATP	AMPK	Aghanoori et al. (2019)
Rat	Whole body, plasma, liver, heart, skeletal muscle	Sprague-Dawley	rhIGF-1 infusion	↑Systemic glucose uptake ↓Plasma Thr, Ser, Gln, Gly, Ala, Val, Ile, Leu, Tyr, Phe ↑Liver, heart, and skeletal muscle glycogen synthesis	NA	Jacob et al. (1989)
Rat	Whole body/liver	Aged Wistar rats	rhIGF-1	↓Serum cholesterol and TG ↑Serum FFA ↑Liver mitochondria membrane potential ↑Liver ATP	NA	García-Fernández et al., (2008)

1. rhIGF-1 = recombinant IGF-1, hIGF-1 = human IGF-1, rIGF-1 = recombinant IGF-1.

2. ↑ indicates and increase, ↓ indicates a decrease.

## Data Availability

Data will be made available on request.
